# Supplementary material for: A reconfigurable photosensitive split-floating-gate memory for neuromorphic computing and nonlinear activation
Source: Nat Commun. 2026 Jan 14;17:1697. doi: 10.1038/s41467-026-68402-7 (PMC12909947; doi:10.1038/s41467-026-68402-7)
Supplement: Supplementary file 1 — Supplementary Information [file 41467_2026_68402_MOESM1_ESM.pdf]

Supplementary Information for

A reconfigurable photosensitive split-floating-gate memory for neuromorphic computing and nonlinear activation

Zhi-Cheng Zhang[1], Yuan Li[1], Jian Yao[2], Zhaolong Chen[3], Fu-Dong Wang[1], Shu-Han Si[4], Yue Ding[1], Hui-Ling Qi[1], Tong-Bu Lu[4], Lixing Kang[2,*], Zhi-Bo Liu[1,5,*], Jian-Guo Tian[1,5,*], Xu-Dong Chen[1,6,*]

[1]The Key Laboratory of Weak Light Nonlinear Photonics, Ministry of Education, School of Physics, Nankai University, Tianjin 300071, China
[2]Division of Advanced Materials, Suzhou Institute of Nano-Tech and Nano-Bionics, Chinese Academy of Sciences, Suzhou 215123, China
[3]School of Advanced Materials, Peking University Shenzhen Graduate School, Peking University, Shenzhen 518055, China
[4]MOE International Joint Laboratory of Materials Microstructure, Institute for New Energy Materials and Low Carbon Technologies, School of Material Science and Engineering, Tianjin University of Technology, Tianjin 300384, China.
[5]State Key Laboratory of Photovoltaic Materials and Cells, Nankai University, Tianjin 300350, China
[6]Academy for Advanced Interdisciplinary Studies, Nankai University, Tianjin 300071, China*E-mail: chenxd@nankai.edu.cn; lxkang2013@sinano.ac.cn; liuzb@nankai.edu.cn; jjtian@nankai.edu.cn.

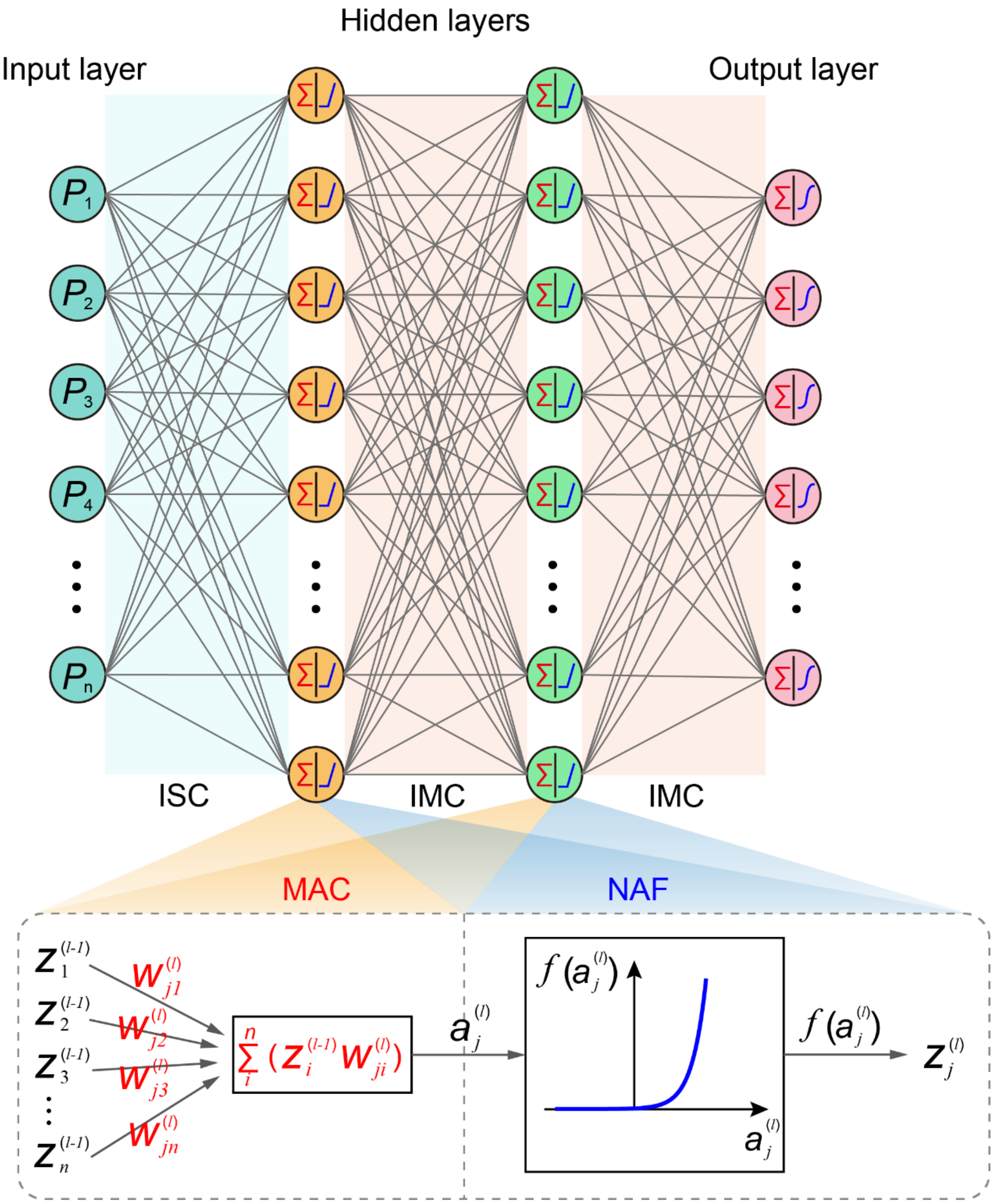

Supplementary Fig. 1| Illustration of a four-layer artificial neural network (ANN). An ANN typically consists of an input layer, multiple hidden layers, and an output layer. Each neuron in the hidden and output layers performs linear multiply-and-accumulate (MAC) operations and then applies nonlinear activation functions (NAFs) to the information from the previous layer. The MAC operation computes the weighted sum of inputs, followed by the application of a NAF, such as ReLU or Sigmoid, to introduce nonlinearity. the output signal is passed as input to the next layer, where it undergoes another round of linear MAC operations and nonlinear activation, continuing through to the output layer. This iterative process enables the neural network to model complex

patterns and perform efficient learning. To prevent gradient vanishing, a ReLU activation function is typically used in the hidden layers, while an exponential-type NAF like Sigmoid is employed in the output layer to amplify differences between output signals. In an NMVS-based ANN, the inputs in the input layer represent the intensity of visual images. The MAC operations between the input layer and the first hidden layer are carried out in ISC hardware, while those in subsequent layers are processed in IMC hardware.

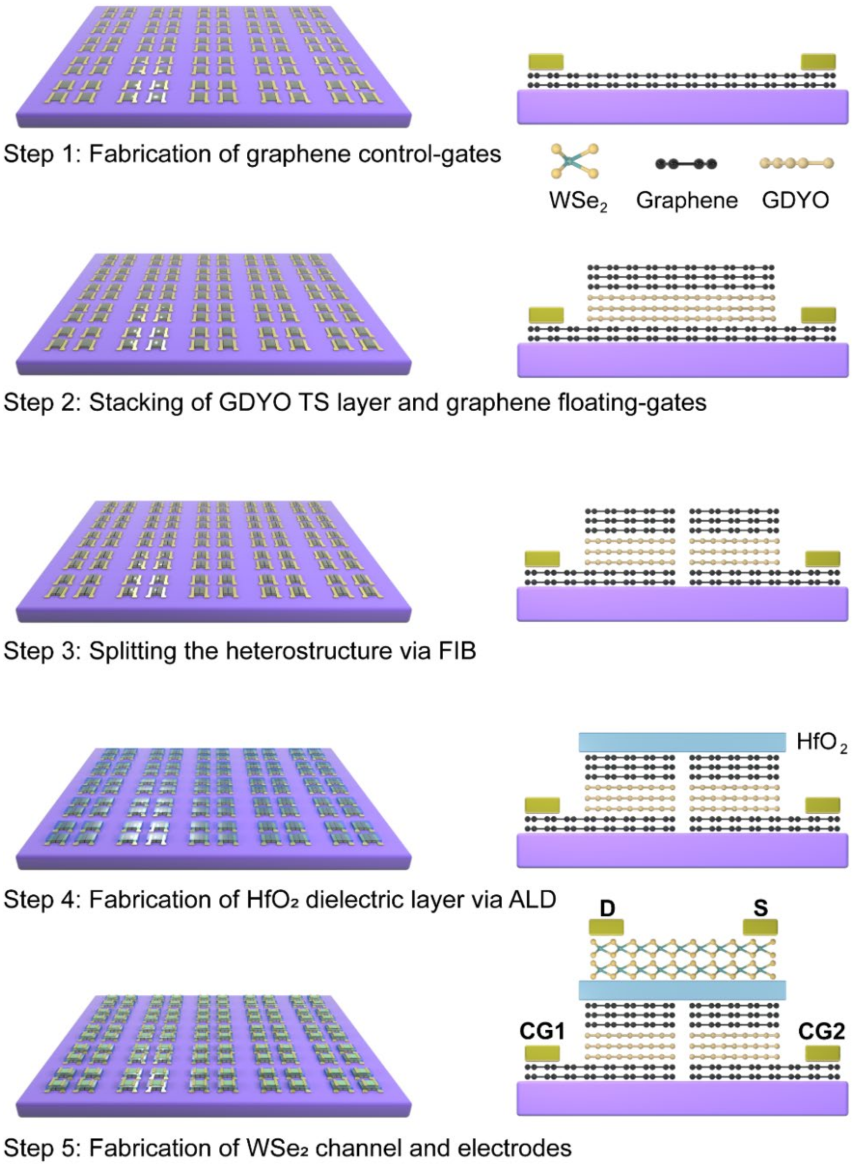

Supplementary Fig. 2| Fabrication process of the MM-SFGM device array. Step 1: Chemical-vapor-deposition (CVD)-grown multilayer graphene film was transferred to a SiO_2/Si substrate, and patterned to square array via the standard photolithography and oxygen plasma etching, acting as the control-gates of the device. Then two Cr/Au (10/50 nm) electrodes were deposited at the left and right edges of each graphene pattern by photolithography, thermal evaporation, and lift-off processes. Step 2: Prepatterned graphdiyne oxide (GDYO) film and multilayer graphene film (MLG) were transferred on top of the graphene control-gate, forming the MLG/GDYO/MLG heterostructure. Step 3: The graphene/GDYO/graphene heterostructure was split by focused ion beam (FIB), with an interval of ~110 nm. Step 4: The HfO_2 dielectric layer with a thickness of 10.5 nm was deposited via atomic layer deposition (ALD). Step 5: CVD-grown multilayer WSe_2 channels (~4.6 nm) were stacked on HfO_2 and the source and drain electrodes (Cr/Au 10/50 nm) were deposited.

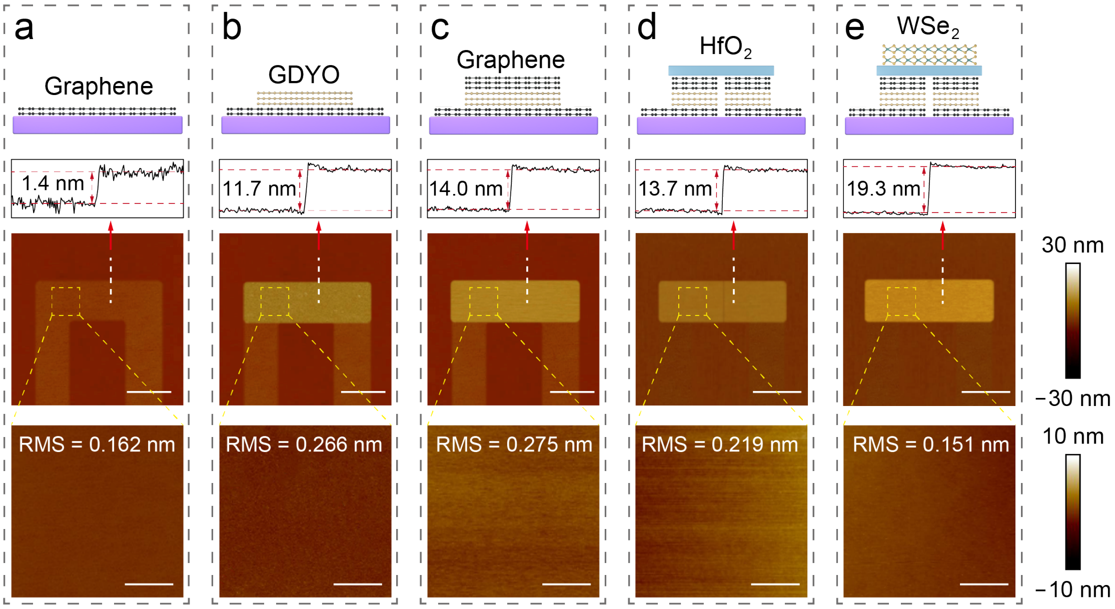

Supplementary Fig. 3| AFM images of the MM-SFGM during fabrication. a–e, AFM images of MLG (**a**), GDYO/MLG (**b**), MLG/GDYO/MLG (**c**), HfO_2/MLG/GDYO/MLG (**d**), and WSe_2/HfO_2/MLG/GDYO/MLG (**e**) heterostructures. The white dashed lines represent the high profiles of the heterostructures, and the yellow boxes mark the surface morphologies of the heterostructures. Scale bars in the middle row, 5 μm. Scale bars in the bottom row, 1 μm.

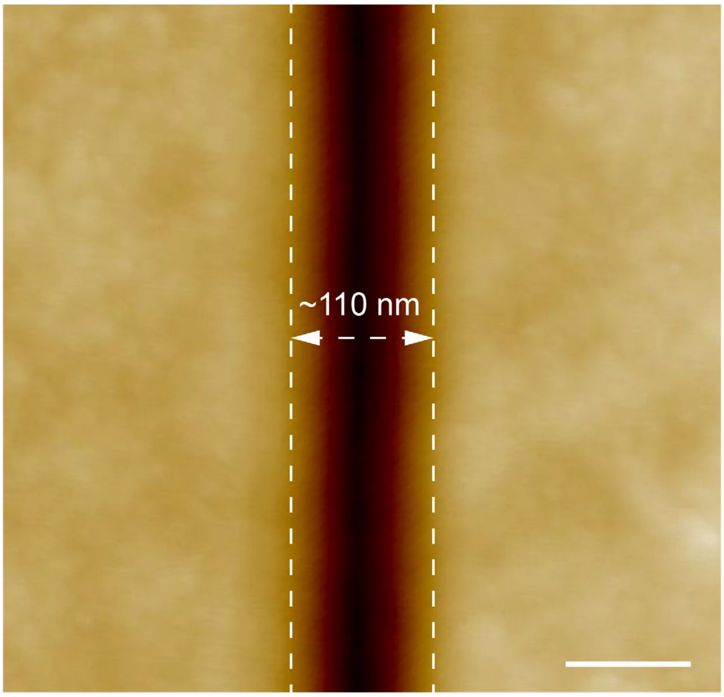

Supplementary Fig. 4| AFM image of the split MLG/GDYO/MLG heterostructure. The interval between the split floating-gates is approximately 110 nm. Scale bar, 100 nm.

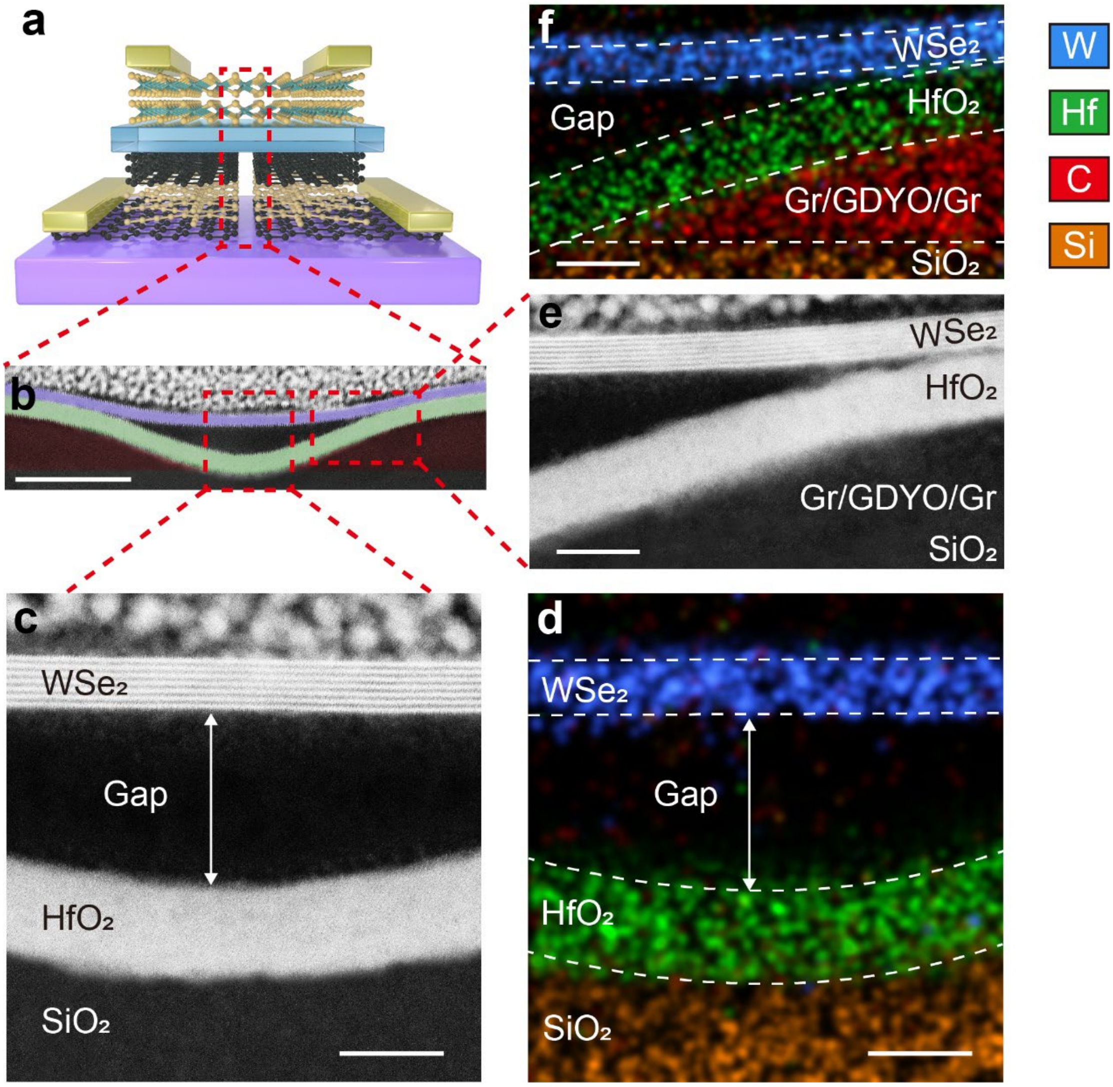

Supplementary Fig. 5| Cross-sectional STEM and EDS images of the MM-SFGM at the split region. a, Illustration of the device structure. **b**, Cross-sectional STEM image of the split region. Scale bar, 50 nm. **c**,**d**, STEM (**c**) and EDS (**d**) images for the central region of the split. Scale bars, 10 nm. **e**,**f**, STEM (**e**) and EDS (**f**) images for the edge region of the split. Scale bars, 10 nm.

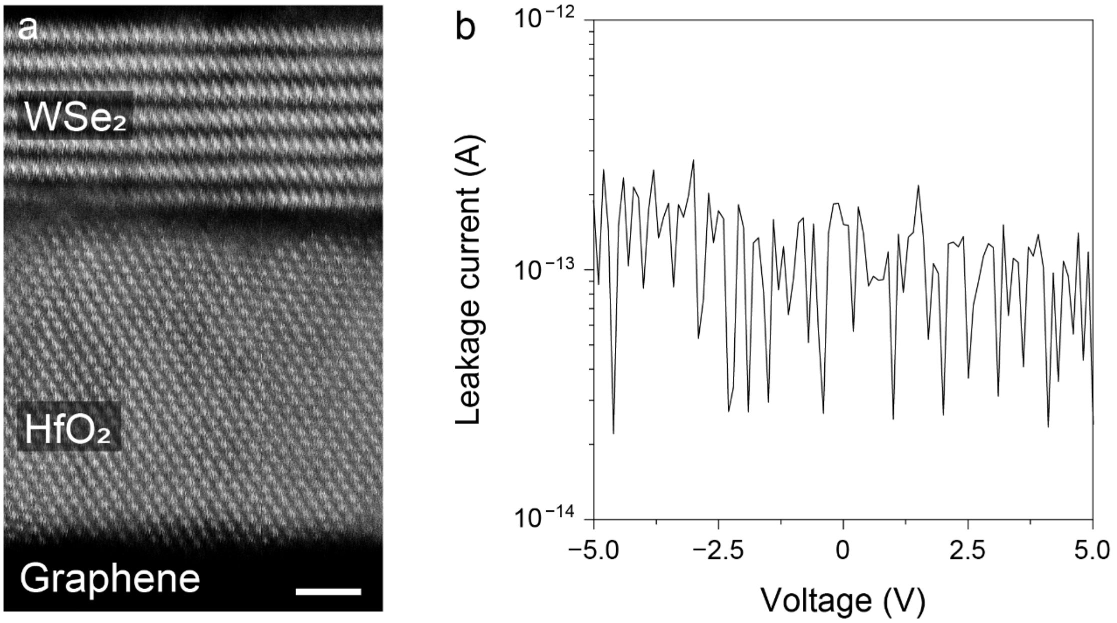

Supplementary Fig. 6| Characterization of the ALD-deposited HfO_2 dielectric layer. a, Dark-field STEM image showing the crystalline structure of the HfO_2 layer. Scale bar, 2 nm. Prior to the deposition of the HfO_2 layer, the underlying MLG was treated with UV-ozone for 10 s to introduce surface dangling bonds. The deposition process was conducted at a constant chamber pressure of 0.15 Torr, utilizing tetrakis(dimethylamido)hafnium (TDMAH) as the Hf precursor and H_2O as the oxygen source, with high-purity nitrogen (14 sccm) serving as the carrier gas. The substrate temperature was maintained at 150°C throughout the deposition process, resulting in a uniform HfO_2 film with a thickness of 10.5 nm. Each ALD cycle consisted of sequential precursor and oxidant pulses followed by purge steps, with pulse/purge durations of 40 ms/90 s for TDMAH and 15 ms/90 s for H_2O. A total of 150 deposition cycles were performed to ensure the formation of a homogeneous and well-controlled thin film with excellent conformality. **b**, Ultralow leakage current for the ALD-deposited HfO_2 dielectric layer on the graphene flake.

Supplementary Note 1: Mechanisms for the high-speed nonvolatile memory with a threshold-switching (TS) layer

1.1 Synthesis of GDYO film

Graphdiyne (GDY) films with controllable thicknesses were first synthesized via an electric-double-layer (EDL)-confined strategy in a routine two-electrode cell (Supplementary Fig. 7a)[1]. Here an Au foil, a Cu foil, and 50 mL acetone were used as the anode, cathode and electrolyte, respectively. Then, 0.25 mL N,N,N',N'-tetramethylethylenediamine (TMEDA) and 0.2 mL HEB monomer solution were introduced into the cell. The voltage between two electrodes was set as 2 V. After 10 h successive reaction in dark environment at 20°C, uniform GDY film was synthesized on Cu foil. Finally, the sample was washed in turn with acetone, dimethylformamide (DMF), ethanol and water, followed by N_2-flow drying. The GDY film on Cu foil was then treated by an UV-ozone cleaner (200 W) for 120 s to form GDYO film. Supplementary Fig. 7b,c show the optical images (OM) of the GDY and GDYO films on a SiO_2/Si substrate, and Supplementary Fig. 7d presents the surface morphology of a 10 nm thick GDYO film, which retains excellent surface flatness with a root mean square (RMS) roughness of 0.266 nm. Supplementary Fig. 8 presents the spectral characterization of GDY and GDYO films. The Raman spectra and Fourier transform infrared spectra (FTIR) reveal the disappearance of the C≡C bonds, confirming the chemical modification of the GDY structure. In addition, FTIR and X-ray photoelectron spectra (XPS) show a significant appearance of C–O, C=O, and O–H bonds. These spectral features indicate that the UV-ozone treatment effectively breaks the alkyne bonds in GDY, introducing a substantial number of oxygen-containing groups into the material. These modifications are essential for the TS behavior and overall performance of the MM-SFGM.

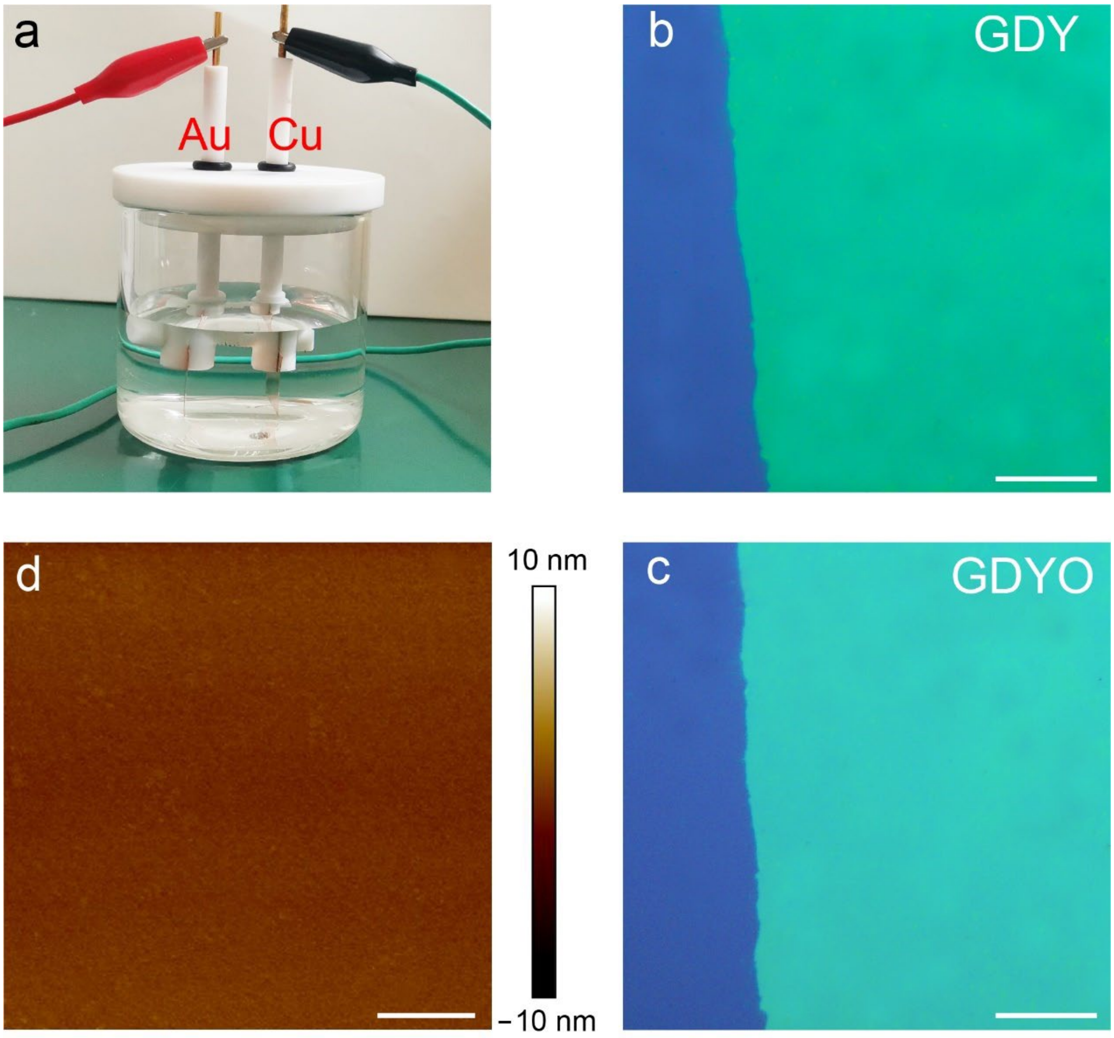

Supplementary Fig. 7| Preparation of GDYO film. a, Experimental setup of the EDL-confined strategy for the synthesis of GDY film. **b**, OM image of the GDY film transferred on a SiO_2/Si substrate. **c**, OM image of the GDYO film prepared by subjecting GDY film to UV-ozone treatment. Scale bars, 50 μm. **d**, AFM image of the GDYO film on SiO_2/Si substrate. Scale bar, 2 μm.

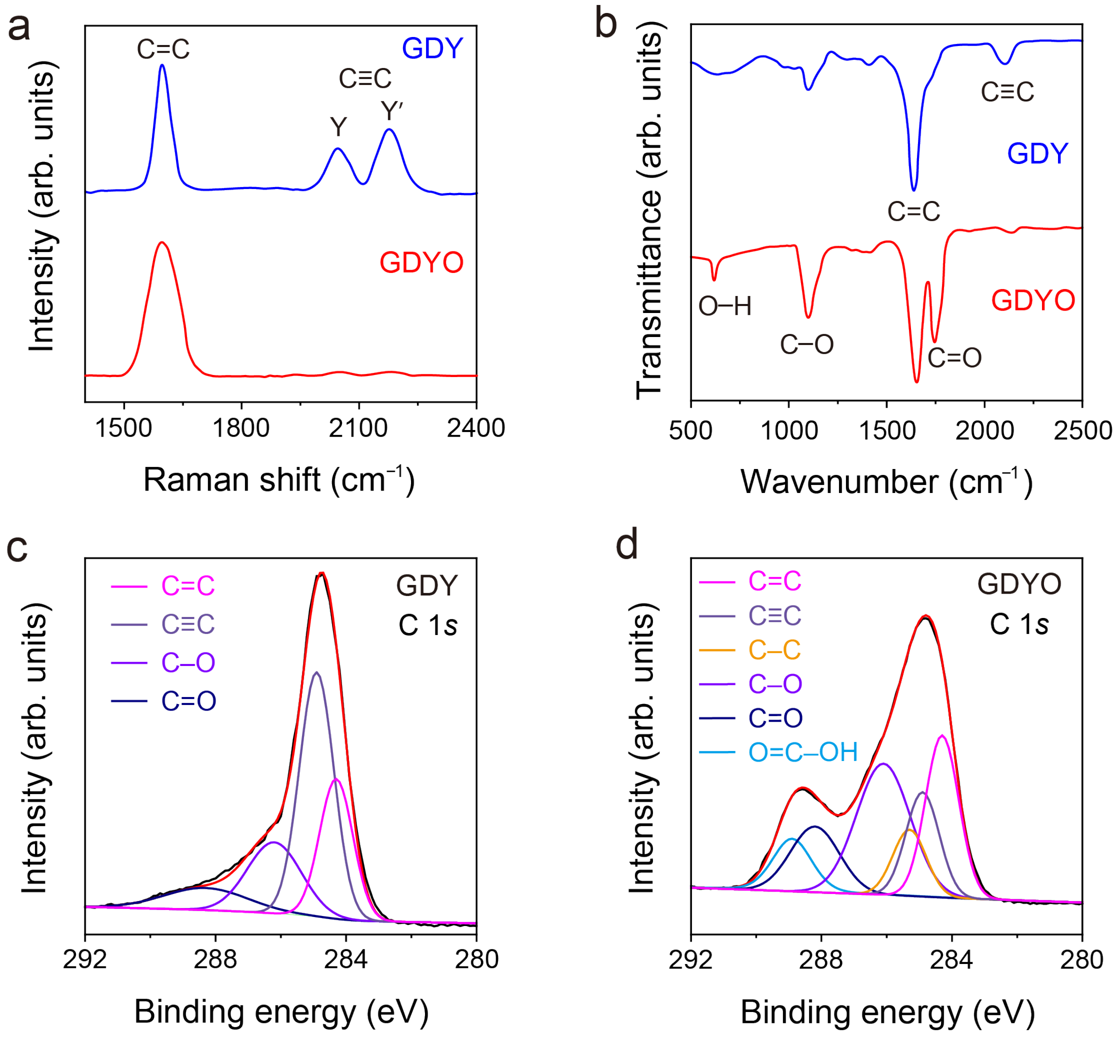

Supplementary Fig. 8| Spectral characterizations of GDY and GDYO. a, Raman spectra of GDY and GDYO films. **b**, FTIR spectra of GDY and GDYO films. **c**,**d**, XPS of C 1s peak for GDY (**c**) and GDYO (**d**) films, respectively. The spectra of GDYO film were measured for the same GDY film after a 120 s UV-ozone treatment.

1.2 Volatile TS characteristics of GDYO layer

The TS performance of the GDYO film was systematically studied by fabricating an MLG/GDYO/MLG vertical heterostructure. The TS characteristics of GDYO films with varying oxidation time were first investigated. As shown in Supplementary Fig. 9, the GDY film without oxidation exhibited good conductivity without resistive-switching behavior. With increasing oxidation time, the GDYO films began to show resistive-switching behaviors, with the on/off ratio improving (as the off-state conductance decreased), but the SET voltage (V_{SET}) also increased. After 300 seconds of UV-ozone treatment, the obtained GDYO became fully insulating and lost its resistive-switching characteristics. Additionally, the thickness of the GDYO layer was also found to significantly influence its resistive-switching properties. Supplementary Fig. 10 presents the resistive-switching characteristics of GDYO layers with different thicknesses. For thin layers (e.g., 5 nm), the SET voltage is low, but the layer fails to completely switch off, leading to high off-state conductance and resulting in charge leakage from the floating gate, which undermines the device's non-volatile storage capabilities. Conversely, thicker GDYO layers (e.g., 15 nm) require a higher SET voltage and longer switching times. To balance the threshold voltage and off-state conductance, a 10 nm thick GDYO layer, treated with UV-ozone for 120 seconds, was selected as the TS layer of the MM-SFGM. Supplementary Fig. 11 demonstrates the resistive-switching characteristics of the 10 nm GDYO layer. Upon applying a ±1 V SET voltage, the GDYO layer switches from the off-state to the on-state within 20 ns. After the voltage pulse ends, the layer spontaneously returns to the off-state, confirming that the GDYO layer exhibits low-voltage, high-speed TS behavior with volatility.

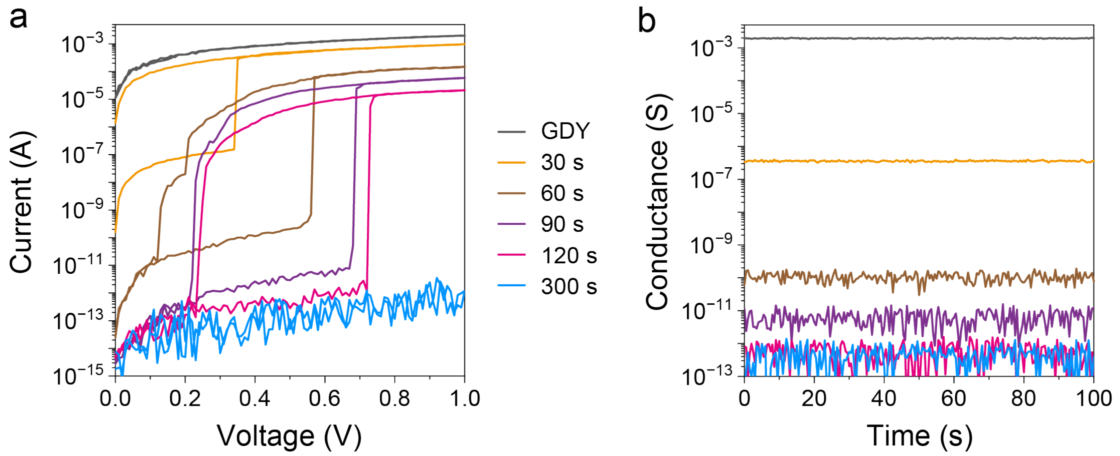

Supplementary Fig. 9| Resistive switching characteristics of the GDYO films with different UV-ozone treatment time. **a**, *I*–*V* curves of the MLG/GDYO/MLG heterostructures with GDYO oxidation time ranging from 30 s to 120 s, using GDY as a reference. **b**, Conductance states of the GDYO films with different oxidation time.

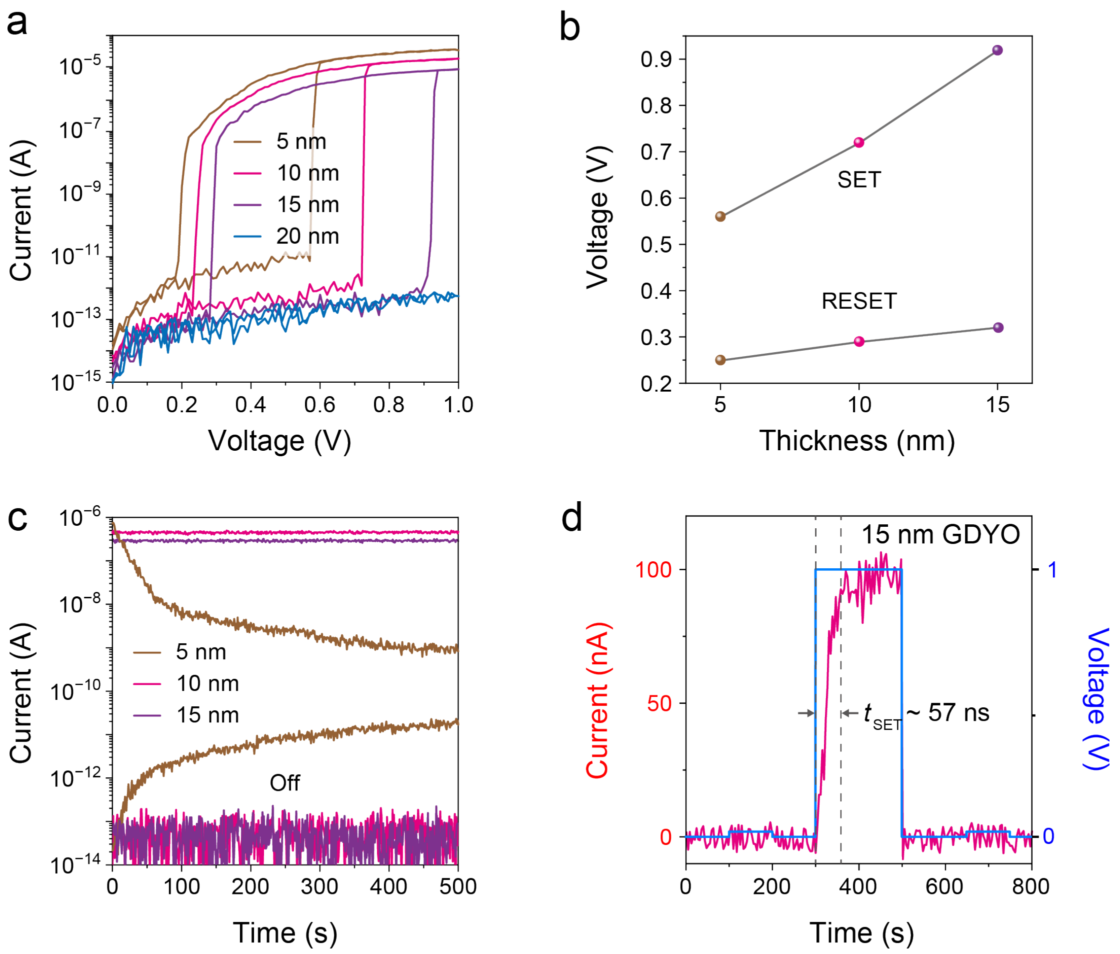

Supplementary Fig. 10| Resistive switching characteristics of the GDYO films with different thicknesses. a, *I*–*V* curves of the MLG/GDYO/MLG heterostructures with GDYO thicknesses ranging from 5 nm to 20 nm. **b**, Distribution of the SET and RESET voltages of the GDYO films with thicknesses of 5 nm, 10 nm, and 15 nm. **c**, Retention characteristics for the on and off states of the GDYO films with thicknesses of 5 nm, 10 nm, and 15 nm. **d**, Transient responses of a 15 nm GDYO film to a +1 V/200 ns voltage pulse. The switching time was measured as 57 ns. +0.1 V/100 ns voltage pulses were applied for read operations before and after the SET operation, demonstrating the volatile behavior.

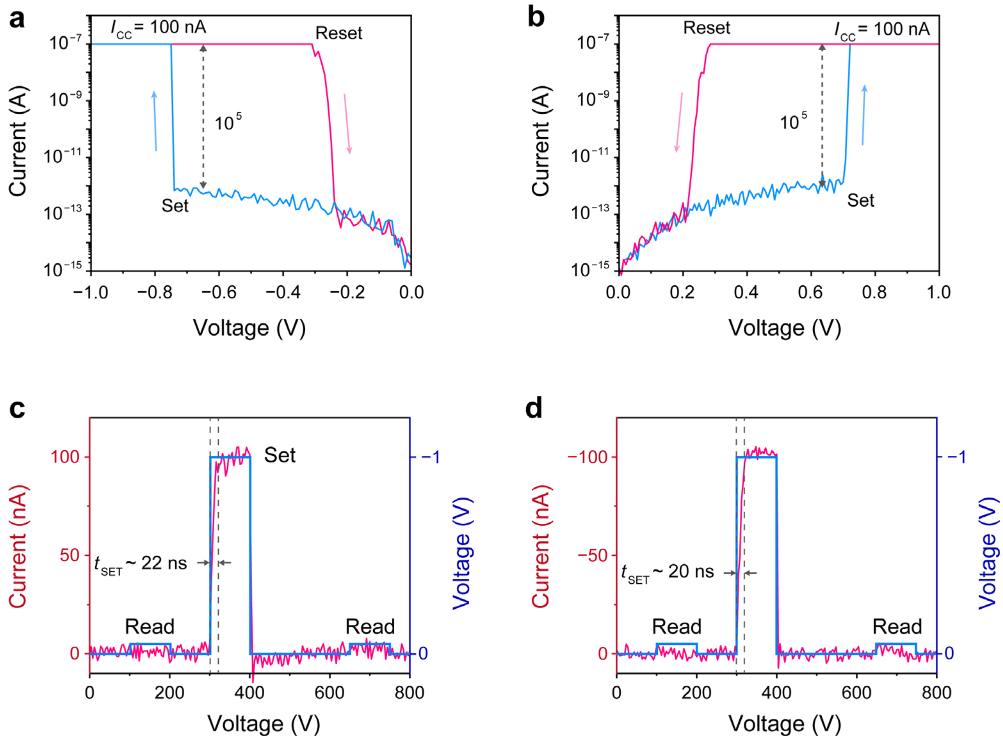

Supplementary Fig. 11| High-speed charge injection through a threshold-switching (TS) layer. a,**b**, Threshold switching characteristics of the Gr/GDYO/Gr heterostructure in negative (**a**) and positive (**b**) sweep voltage regimes. Compliance current (I_{CC}) was set as 100 nA. **c**,**d**, Transient responses of the GDYO TS layer to a –1 V/100 ns (**c**) and +1 V/100 ns (**d**) voltage pulse, respectively. The switching time were measured as 22 ns and 20 ns, respectively. ±0.1 V/100 ns voltage pulses were applied for read operations before and after the SET operation, demonstrating the volatile behaviors.

1.3 Mechanism for the volatile TS behavior of GDYO layer

The volatile threshold-switching (TS) behavior of the GDYO layer is attributed to the migration and spontaneous diffusion of oxygen-containing groups, driven by voltage bias and concentration gradients, respectively. As shown in Supplementary Fig. 8, oxygen-containing groups such as –COOH and –OH, introduced by UV-ozone treatment, readily bind to the active C≡C bonds in GDY, disrupting the GDY structure and causing a loss of conductivity. The porous nature of the GDYO layer facilitates the easy migration of these groups between layers. Simulation results show that the migration barriers for –COOH and –OH groups are low, at just 0.49 eV and 0.65 eV, respectively (Supplementary Fig. 12). Consequently, under voltage bias, these groups migrate, and the reformation of C≡C bonds in specific regions forms GDY conductive filaments (CFs), causing GDYO to switch from a high-resistance state to a low-resistance state. Upon removal of the voltage bias, oxygen-containing groups accumulate in certain regions, creating a concentration gradient. Driven by this gradient, the groups spontaneously diffuse from high-concentration to low-concentration areas and recombine with the C≡C bonds, leading to the rupture of the GDY CFs and the transition of the GDYO layer back from a low-resistance to a high-resistance state.

Supplementary Fig. 13 presents a cross-sectional scanning transmission electron microscope (STEM) image of the MLG/GDYO/MLG heterostructure, revealing some well-crystallized GDY domains. The growth and fracture of these GDY domains determine the switching state of the GDYO layer. Although this process was not directly observed using TEM, it was confirmed through Raman mapping and conductive atomic force microscope (CAFM) measurements. As shown in Supplementary Fig. 14, the Raman spectrum of the GDYO layer exhibits a quite weak C≡C signal prior to the application of a voltage bias. When a 1 V voltage bias is applied, the Y and Y' peaks at 2042 cm^{-1} and 2173 cm^{-1} significantly intensify, indicating the formation of well-crystallized GDY domains with the GDYO. Upon removal of the voltage bias, these peaks diminish and return to their initial intensities, suggesting a reduction in the GDY domains. These observations are further supported by CAFM measurements, as shown

in Supplementary Fig. 15. When a 0.2 V bias (below the SET voltage) is applied, the measured current in GDYO is only a few picoamperes, close to the detection limit of the instrument, confirming that the GDYO is in a high-resistance state. With the application of a 1 V bias, the current increases to approximately 10 nA, demonstrating a transition to a low-resistance state. When the bias is returned to 0.2 V, the current drops back to the picoampere range, indicating that GDYO reverts to its high-resistance state. CAFM measurements further highlighted the localized and controlled conductivity changes within the GDYO material. These results provide clear evidence of the formation and rupture of GDY CFs, driven by the migration of oxygen-containing groups. This process is responsible for the volatile TS behavior of the GDYO layer.

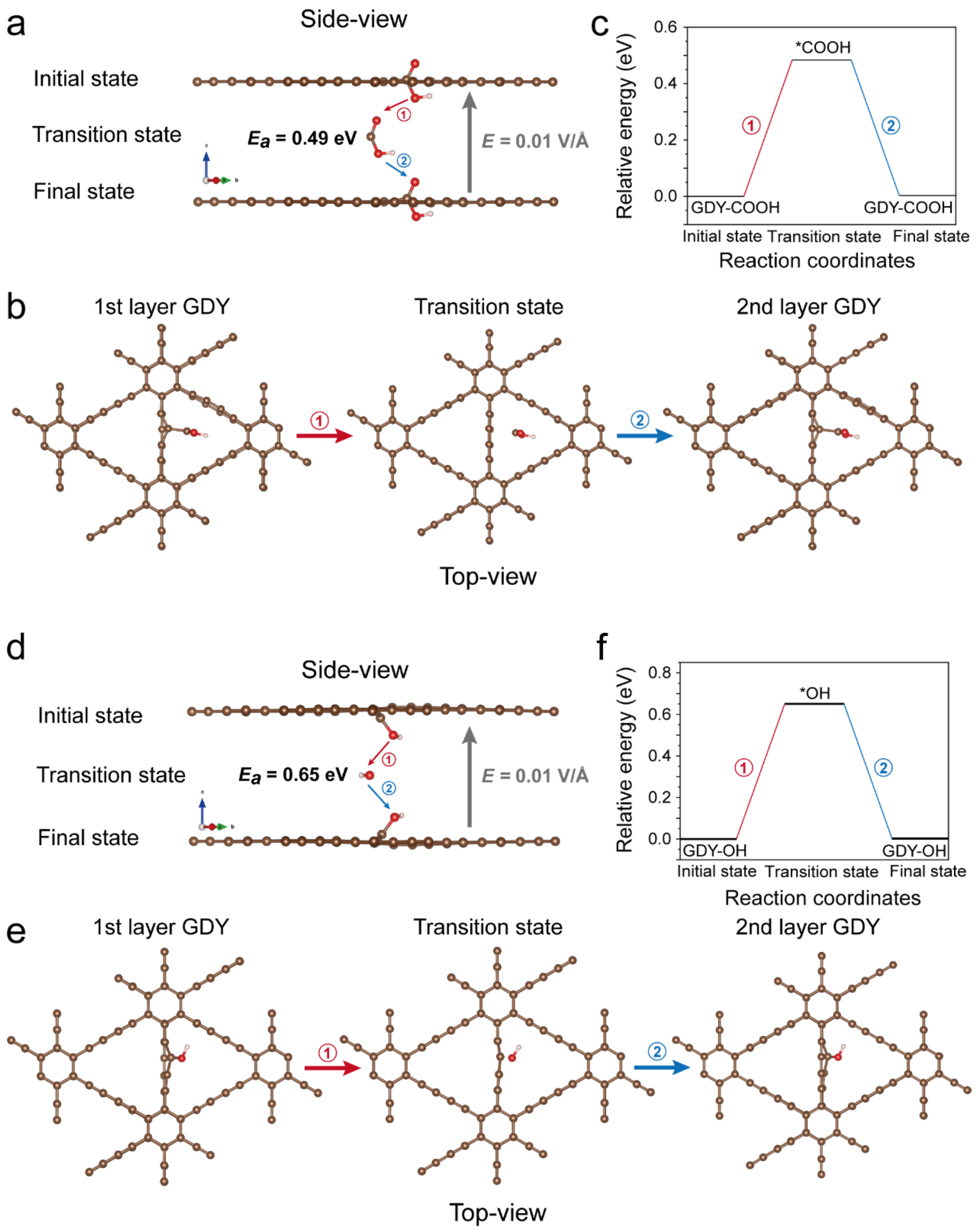

Supplementary Fig. 12| Density functional theory (DFT) calculations for the diffusion barriers of oxygen-containing groups in GDYO. a–c, Side-view (**a**) and top-view (**b**) of a *COOH group diffusion between adjacent GDYO layers and corresponding diffusion energy profile (**c**). **d–f**, Side-view (**d**) and top-view (**e**) of a *OH group diffusion between adjacent GDYO layers and corresponding diffusion energy profile (**f**).

Computational details: We carried out all the DFT calculations in the Vienna *ab initio* simulation (VASP6.3.0) code[2]. The exchange-correlation is simulated with PBE functional and the ion-electron interactions were described by the PAW method[3,4]. The

vdWs interaction was included by using empirical DFT-D3 method[5]. Two layers of GDYO (a = 16.40, b = 19.00 Å, c =20 Å; $\alpha = \beta = \gamma = 90°$) was used to simulate the *COOH and *OH group adsorption and diffusion. All atoms are allowed to move freely during the simulation. The Monkhorst-Pack-grid-mesh-based Brillouin zone k-points are set as 2 × 2 × 1 with the cutoff energy of 450 eV. The convergence criteria are set as 0.02 eV A^{-1} and 10^{-5} eV in force and energy, respectively. The adsorption energy was calculated as $E_{adsorption} = E_{total} - (E_{substrate} + E_{adsorbate})$, where the $E_{substrate}$, $E_{adsorbate}$ and E_{total} were the DFT energy of the catalyst substrate, the adsorbate and the total system, respectively. The transition states activation barrier of *COOH and *OH group diffusion were obtained from climbing-image nudged elastic band (CI-NEB) calculations[6].

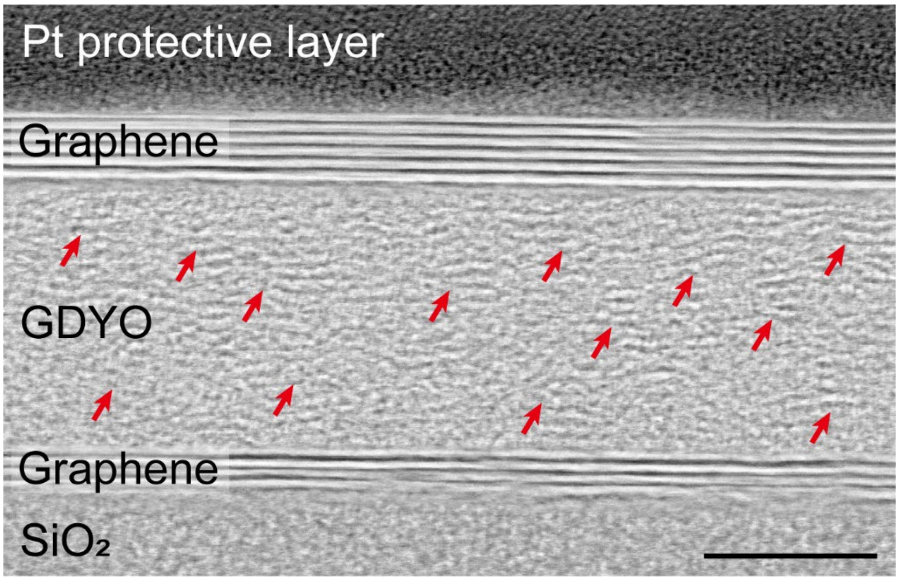

Supplementary Fig. 13| Cross-sectional STEM image of an MLG/GDYO/MLG heterostructure. Red arrows mark well-crystallized GDY domains in GDYO film. Scale bar, 5 nm.

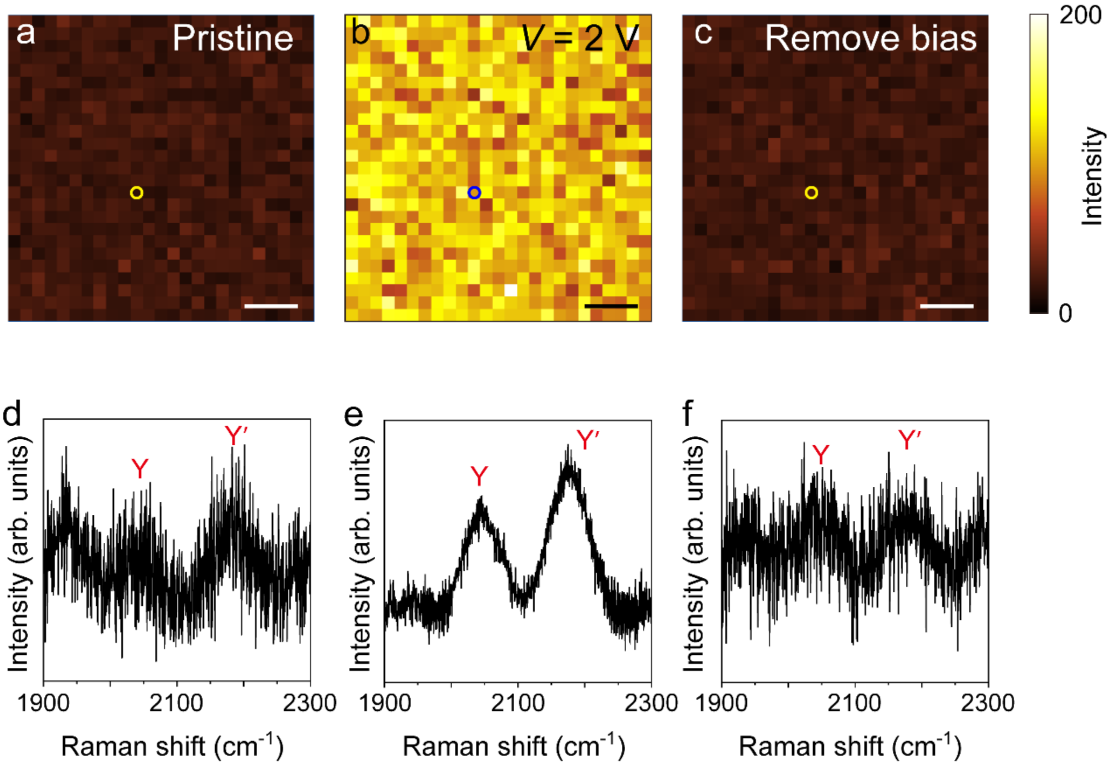

Supplementary Fig. 14| Raman mapping images of a GDYO film. a–c, Raman Y' band maps of a GDYO film at pristine state (**a**), applying a 1 V bias voltage (**b**) and after removing the bias voltage (**c**). Scale bars, 10 µm. **d–f**, Raman profiles of the circular points marked in **a–c**, respectively. Since the main oxidation sites of GDY are the *sp*-hybrid carbon atoms, the Raman Y' band corresponding to C≡C bond has an obvious difference between GDY and GDYO. For the pristine GDYO before applying bias voltage, its Raman Y’ band is quite weak. While applying a 2 V bias voltage, the intensity of Raman Y' band has a significant enhancement, demonstrating the formation of GDY as the conductive filaments. After removing the bias voltage, the intensity of Raman Y' band returns to its initial level, which indicates that the GDY filaments are disappeared.

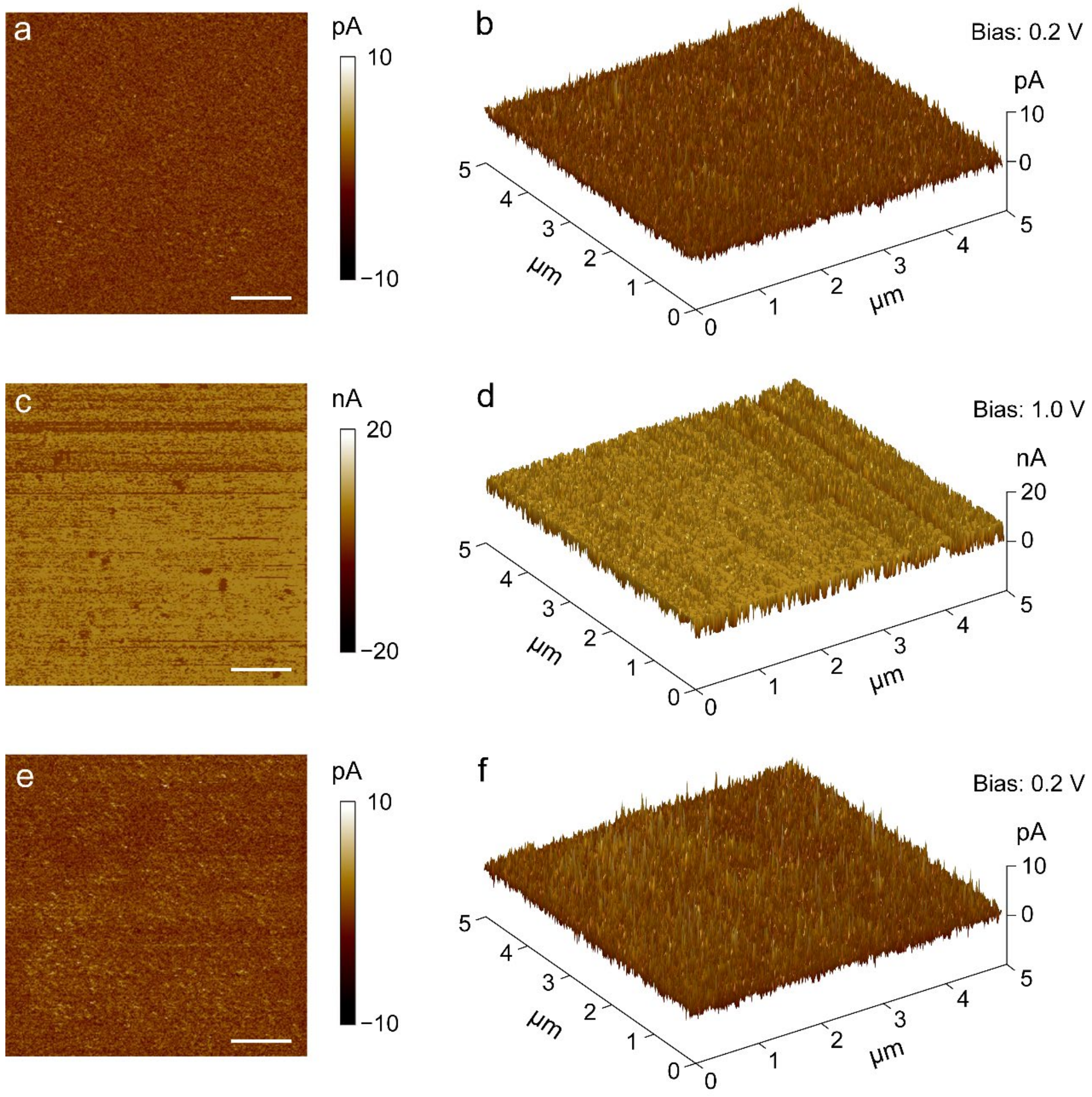

Supplementary Fig. 15| CAFM images of a GDYO film. 2D (**a**,**c**,**e**) and 3D (**b**,**d**,**f**) profiles of the CAFM images for a 10 nm GDYO film. **a**,**b**, While a bias voltage (0.2 V) smaller than V_{SET} (approximately 0.7 V) was applied, the GDYO film remains at HRS with currents of several picoamperes. **c**,**d**, While a bias voltage (1.0 V) exceeding V_{SET} was applied, the GDYO film switched to LRS with currents of approximately 10 nA, demonstrating the formation of GDY CFs. **e**,**f**, Followed by the measurement in **c**, a bias voltage of 0.2 V was applied again, and the GDYO film returned to its HRS with a picoampere current level, which indicates the rupture of GDY CFs after removing the bias voltage. Notably, the picoampere current level in **a** and **e** is the detection limit of the instrument.

1.4 Mechanism for high-speed non-volatile memory with a TS layer

The nanosecond-scale switching time and low SET voltage of the GDYO TS layer enable the MM-SFGM to perform high-speed programming operations at low operation voltages. As illustrated in Supplementary Fig. 16a, when a negative V_{CG} pulse with nanosecond duration is applied to the device, the GDYO TS layer switches from off state to the on state within a few nanoseconds, facilitating the direct injection of electrons from the control gate to the floating gate. After the nanosecond V_{CG} pulse, the GDYO TS layer spontaneously returns to its initial off state, preventing the escape of the injected electrons in the floating gate (Supplementary Fig. 16b). Similarly, a positive V_{CG} pulse enables the direct injection of positively charged holes, which are then confined in the floating gate. Notably, the TS mechanism of our device is fundamentally different from the charge tunneling mechanism used in traditional flash memory. In flash memory, charge injection occurs via quantum tunneling through an insulating barrier, which always requiring a larger programming voltage and longer duration.

The injected electrons and holes generate a local electrostatic field that modulates the doping levels of the channel. By controlling the type of injected charges in the two floating gates, various homojunctions—such as p-n, n-p, n-n, and p-p—can be formed in the ambipolar WSe_2 channel. As shown in Supplementary Fig. 17, when ±2 V/20 ns V_{CG} pulses are applied to CG1 and CG2, the channel conductance exhibits stable retention for over 10^5 s across all four homojunction configurations.

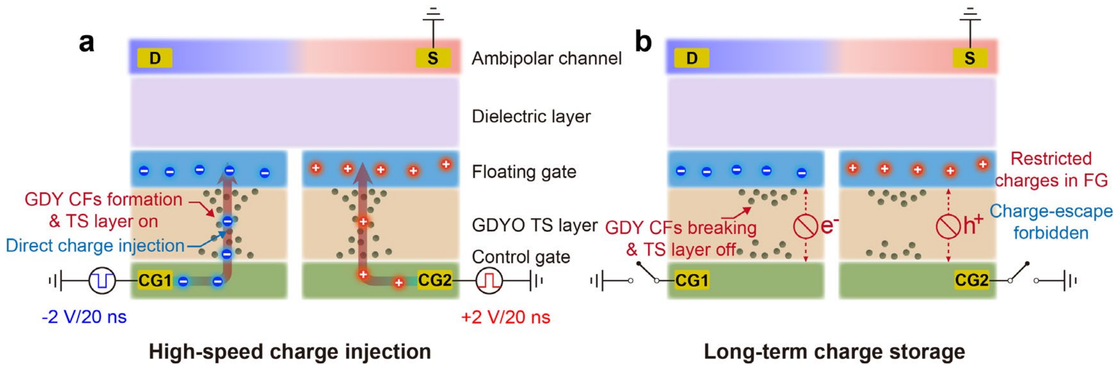

Supplementary Fig. 16| Mechanism for high-speed non-volatile memory with a TS layer. a, Illustration of the high-speed charge injection through a TS layer. While a –2 V/20 ns (+2 V/20 ns) V_G pulse is applied on the CG1 (CG2), the GDYO TS layer switches on, and electrons (holes) are directly injected into the floating gate through the TS layer. **b**, Illustration of the long-term charge storage in the floating gate. After withdrawing the V_G pulse, the GDY CFs break spontaneously and the TS layer switches off, forbidding the escape of the injected electrons and holes and thus resulting in a long-term storage of charges in the floating gate.

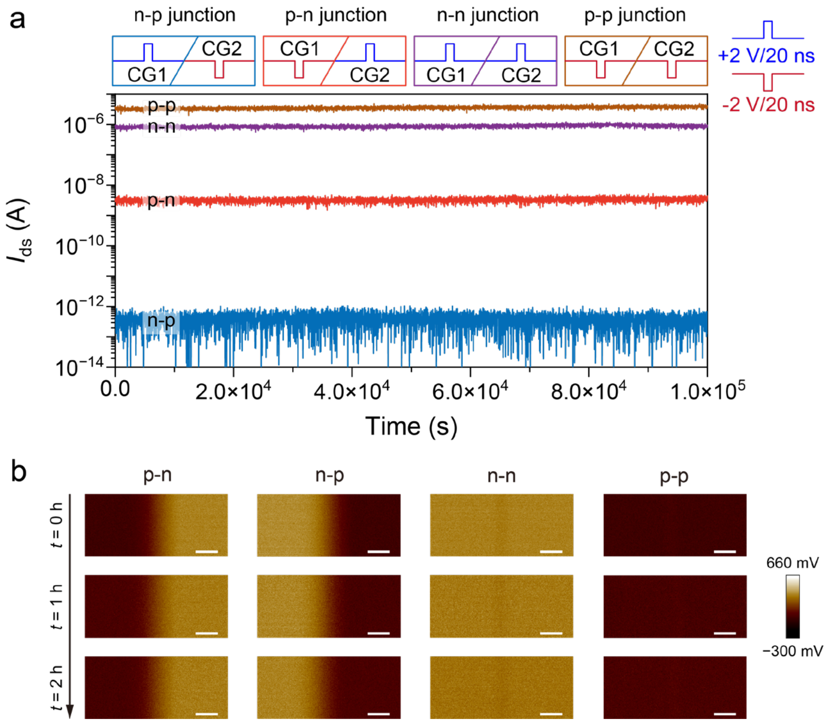

Supplementary Fig. 17| Long-term retention of the MM-SFGM with p-n, n-p, n-n, and p-p homojunctions. a, Retention characteristics of the MM-SFGM with four different states. The upper panel illustrates the configuration of the V_G pulses that applied to CG1 and CG2 for n-p (blue), p-n (red), n-n (green), and p-p (brown) homojunctions, where the red and blue waveforms represent a –2 V/20 ns and a +2 V/20 ns V_G pulse, respectively. After applying corresponding V_G pulses to CG1 and CG2, the channel currents of n-p, p-n, n-n, and p-p homojunctions were monitored via a V_{ds} = 0.5 V. **b**, KPFM images of the homojunction with four different states. KPFM measurements were performed at t = 0 h, 1 h, and 2 h, respectively. The KPFM images demonstrate that the formed homojunctions can maintain for a long time without applying external gate voltage. Scale bars, 500 nm.

1.5 Unified physical mechanism for multifunctional operation

The multifunctional capabilities of the MM-SFGM device—spanning ISC, IMC, and hardware-based NAFs—are all governed by a unified physical model based on TS-assisted charge injection and electrostatic modulation.

As detailed in Supplementary Note 1.2–1.4, the GDYO layer serves as a volatile threshold-switching medium, enabling nanosecond-scale charge injection into two spatially separated floating gates. Under nanosecond voltage pulses (±2 V, 20 ns), the TS layer transiently switches to a conductive state, permitting the injection of electrons or holes into the floating gates depending on pulse polarity. Once the pulse ends, the TS layer spontaneously returns to its high-resistance state, securely trapping the injected charges and ensuring non-volatile functionality.

The key to the MM-SFGM's versatility lies in how these injected charges electrostatically modulate the WSe_2 channel. By selectively configuring the amount and type of stored charges in each gate, the device can dynamically form and modulate different doping profiles and junction states (e.g., p-n, n-p, n-n, p-p). These junctions directly determine the device's electrical behavior:

- In the ISC mode, charges of opposite polarity are injected into the two floating gates, forming an internal p–n or n–p junction. This junction facilitates efficient photocarrier separation under illumination via the photovoltaic effect, leading to tunable photoresponsivity and photocurrent generation.
- In the IMC mode, charges of the same polarity are injected into both gates, uniformly shifting the channel Fermi level and tuning the electrostatic doping level across the entire channel. This allows for quasi-linear, analog conductance modulation used for synaptic weight storage.
- In the NAF mode, the built-in electric field across the p–n junction is modulated by asymmetrically controlling the amount and polarity of stored charges. This enables programmable rectifying *I–V* behavior, allowing the device to emulate nonlinear activation functions such as ReLU and Sigmoid.

This single-material, charge-configuration-based mechanism ensures that ISC, IMC,

and NAFs can all be dynamically accessed via the same device structure, with nanosecond switching speed, sub-10 fJ programming energy, and robust non-volatility. Moreover, as all modes derive from a shared physical origin—electrostatic modulation via charge trapping—this architecture offers a coherent pathway for reconfigurable neuromorphic computing with minimal circuit overhead.

1.6 Advantages of the GDYO TS layer

The key advantages of GDYO that made it the ideal choice for this work are as follows:

i) **Uniform porous structure of GDYO.** GDYO possesses a uniform porous structure that enables the efficient migration of oxide-containing groups such as -COOH and -OH. Theoretical calculations indicate that the migration barrier for these groups is below 0.5 eV (Supplementary Fig. 12), which is crucial for enabling fast switching behavior in the device. This characteristic allows for efficient and low-voltage programming, making GDYO ideal for high-speed applications.

ii) **No Forming Process Required.** Unlike many resistive-switching materials, GDYO does not require a forming process. This eliminates the variability often introduced by the forming step, ensuring improved consistency and reliability across the device array. By avoiding the forming process, we achieve uniform device behavior (Supplementary Note 4), which is critical for scalable systems.

iii) **Compatibility with 2D Materials.** As a 2D material, GDYO offers excellent compatibility with other 2D materials, such as graphene, through van der Waals interactions. This allows for seamless integration without damaging the underlying layers, unlike traditional oxide materials, which can be deposited using methods like magnetron sputtering and potentially harm the underlying layers.

These features make GDYO an excellent choice for low-voltage, high-speed devices with stable and reliable switching performance. As demonstrated in Supplementary Note 4, GDYO-based devices exhibit minimal device-to-device variation (7% for weight updating and 10% for nonlinear activation), which is crucial for maintaining the

high computation accuracy of the neuromorphic systems we have developed.

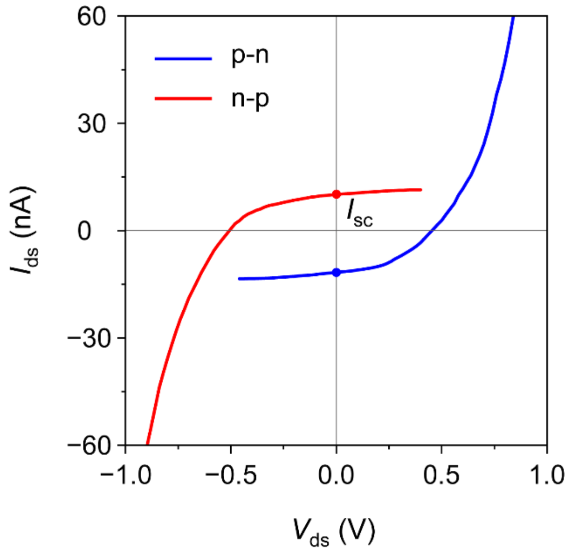

Supplementary Fig. 18| *I*-*V* curves of the p-n (blue) and n-p (red) homojunctions under illumination. The blue and red dots at zero voltage represent the short-current I_{sc} of the p-n and n-p junctions, respectively.

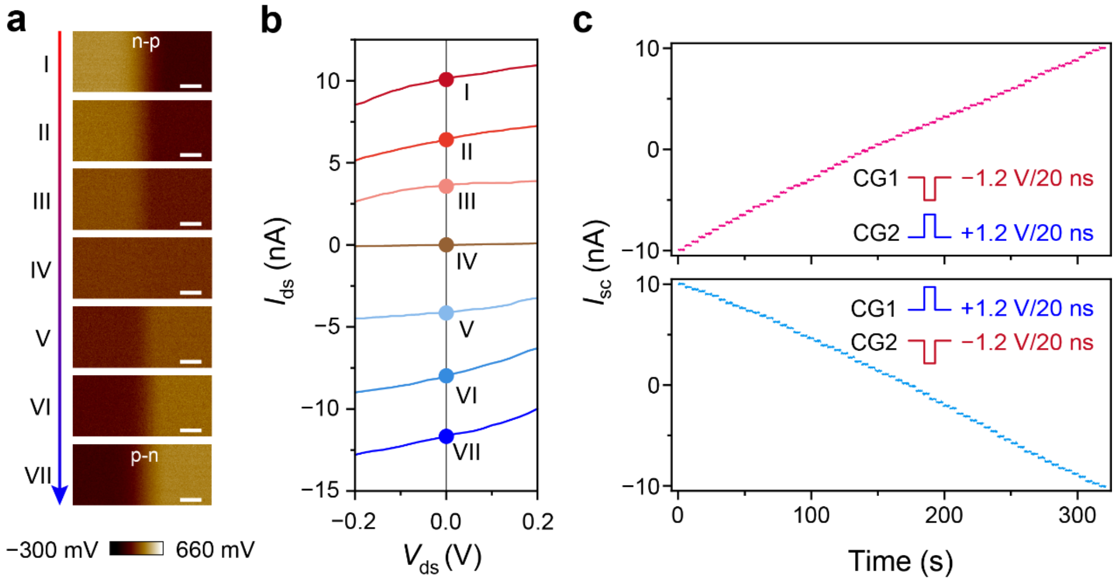

Supplementary Fig. 19| Dynamic modulation of the photoresponsivity between negative and positive regimes. a, KPFM images of the intermediate junction states from n-p junction (state I) to p-n junction (state VII). Scale bars, 500 nm. **b**, *I*-*V* curves of the device at intermediate states under illumination. The dots at zero voltage represent the I_{sc} generated by the intermediate junctions with corresponding serial numbers (I–VII). **c**, 63 levels of cumulative positive and negative photocurrents with uniform increments and non-overlapping. The photocurrents were measured at the short-circuit condition (I_{sc}). While –1.2 V/20 ns and +1.2 V/20 ns V_G pulses are applied on CG1 and CG2, respectively, the I_{sc} gradually changes from negative to positive (pink curves), and an opposite modulation of I_{sc} is realized by applying +1.2 V/20 ns and –1.2 V/20 ns V_G pulses to CG1 and CG2, respectively (blue curves).

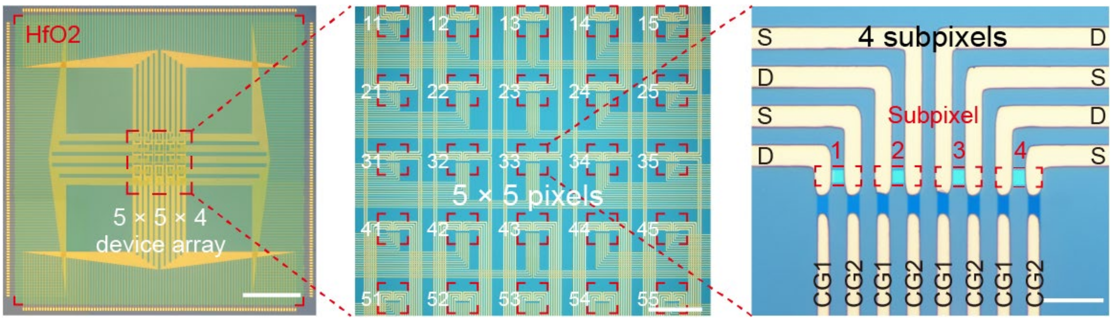

Supplementary Fig. 20| Optical images of a 5 × 5 × 4 device array for ISC and IMC operations. The device array consists of 5 × 5 pixels, and each pixel contains 4 subpixels. Scale bars (from left to right), 1 mm, 150 µm, and 10 µm, respectively.

Supplementary Note 2: Configurations of the MM-SFGM for conductance modulation

2.1 Multiple programming pathways and operational modes

As shown in Supplementary Fig. 21, the conductance of the MM-SFGM can be dynamically modulated through five distinct pathways between high and low conductance states. These pathways are based on different charge configurations stored in the split floating gates, which electrostatically modulate the doping level of the ambipolar WSe_2 channel. Each configuration exhibits unique characteristics in terms of switching ratio, linearity, retention, and operation speed, allowing the system to flexibly adapt to various application requirements such as non-volatile memory, analog computing, or hybrid neuromorphic functions.

To better guide their usage, we define two representative operational modes. Mode-1 emphasizes non-volatile memory behavior, where high on/off ratios, long data retention, fast programming speed, and robust endurance are desired. This mode is suitable for scenarios where discrete, stable states are sufficient, such as digital neural networks or storage tasks. In contrast, Mode-2 targets analog IMC, requiring linear, symmetric, and multi-level conductance updates to support high-accuracy analog in-memory computing, neuromorphic computing, and matrix-vector multiplication tasks. Supplementary Table 1 summarizes the key performance parameters associated with each modulation pathway under these two modes.

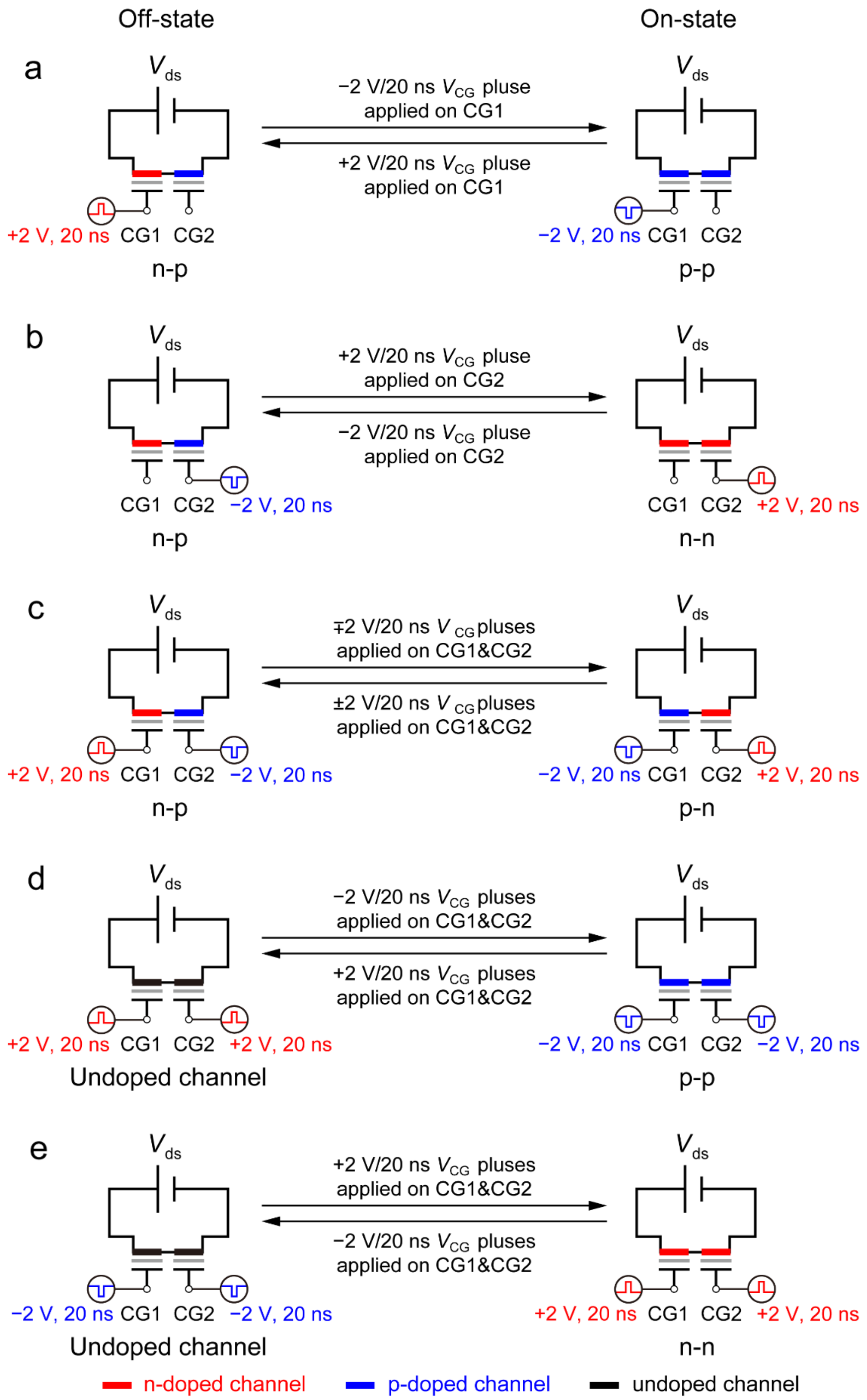

Supplementary Fig. 21| Configurations and pathways of the MM-SFGM for switching between low- and high-conductance states. a, Switching between n-p (off) and p-p (on) states by applying a –2 V/20 ns V_{CG} pulse (n-p to p-p) and a +2 V/20 ns V_{CG} pulse (p-p to n-p) to CG1. **b**, Switching between n-p (off) and n-n (on) states by applying a +2 V/20 ns V_{CG} pulse (n-p to n-n) and a –2 V/20 ns V_{CG} pulse (n-n to n-p)

to CG2. **c**, Switching between n-p (off) and p-n (on) states by applying paired $\mp$2 V/20 ns V_{CG} pulses (n-p to p-n) and paired $\pm$2 V/20 ns V_{CG} pulses (p-n to n-p) to CG1 and CG2. **d**, Switching between intrinsic undoped state (off) and heavily p-doped state (on) by applying paired –2 V/20 ns V_{CG} pulses (undoped to p-doped) and paired +2 V/20 ns V_{CG} pulses (p-doped to undoped) to CG1 and CG2. **e**, Switching between intrinsic undoped state (off) and heavily n-doped state (on) by applying paired +2 V/20 ns V_{CG} pulses (undoped to n-doped) and paired –2 V/20 ns V_{CG} pulses (n-doped to undoped) to CG1 and CG2.

Supplementary Table 1. Key parameters of the device under representative conductance modulation configurations

	Channel states	n-p to p-p	n-p to n-n	n-p to p-n	Undoped to p-p	Undoped to n-n
	Off	n-p	n-p	n-p	Undoped channel	Undoped channel
	On	p-p	n-n	p-n	p-doped channel	n-doped channel
Key parameters for mode-1	Dynamic range (µS)	10^{-6} to 10	10^{-6} to 1	10^{-6} to 10^{-1}	10^{-5} to 10	10^{-5} to 1
	On/off ratio	10^{7}	10^{6}	10^{5}	10^{6}	10^{5}
	Retention	>10^{5} s	>10^{5} s	>10^{5} s	>10^{5} s	>10^{5} s
	Endurance	>10^{6}	>10^{6}	>10^{6}	>10^{6}	>10^{6}
	Read voltage (V_{ds}) [a]	0.1 V	0.1 V	0.5 V	0.1 V	0.1 V
	Program voltage (V_{CG}) [b]	±2 V, 20 ns	±2 V, 20 ns	±2 V, 20 ns	±2 V, 20 ns	±2 V, 20 ns
	Energy consumption [c]	4 fJ	4 fJ	8 fJ	8 fJ	8 fJ
Key parameters for mode-2	Dynamic range (µS)	0.1 to 7	0.03 to 1.7	0.005 to 0.12	0.1 to 7	0.03 to 1.5
	Linearity ($\|R\|^2$)	0.805/0.753	0.822/0.764	0.421/0.387	0.995/0.977	0.990/0.973
	Symmetry	235	218	135	746	532
	Available states	22	19	15	63	63
	CCV	8.4%/9.6%	7.9%9.2%	10.6%/9.8%	3.3%/3.1%	3.5%/3.7%
	Program voltage (V_{CG})	±2 V, 20 ns	±2 V, 20 ns	±2 V, 20 ns	±1.2 V, 20 ns	±1.2 V, 20 ns
	Energy consumption	4 fJ	4 fJ	8 fJ	4.8 fJ	4.8 fJ
	Accuracy [d]	71%	69%	63%	96%	95%

[a] Read voltage refers to the V_{ds} applied during the reading process. A V_{ds} of 0.5 V is required for the p-n junction due to the build-in potential, while a V_{ds} of 0.1 V is applied in other cases.
[b] Program voltage refers to the V_{CG} applied to the control gates to switch the conductance between the on/off states (for memory) or between different conductance levels (for synapses).
[c] The energy consumption is calculated using the equation of $E = V_{CG} \times I_{GS} \times t$, which refers to the energy used for switching the conductance between the on/off states (for memory) or between different conductance levels (for synapses).
[d] The simulated results with the corresponding parameters for MNIST dataset.

2.2 Mode-1: high-retention and discrete-state programming for memory applications

Among the available programming configurations, transitions between n-p and p-n states deliver the highest on/off ratio, exceeding 10^7 (Supplementary Fig. 22), and exhibit excellent long-term data retention (>10^5 seconds) and endurance (>10^6 cycles), as confirmed by measurements in Supplementary Fig. 23. These characteristics make this configuration especially suitable for digital memory applications or digital neural networks that require discrete, non-volatile conductance states. Similarly, switching between n-p and n-n states also yields strong memory characteristics, albeit with a slightly lower on/off ratio (~10^6). These two pathways can be interpreted as the programmable p-type and n-type memory modes of the device, providing robust storage functionality under low-voltage, nanosecond-scale programming conditions.

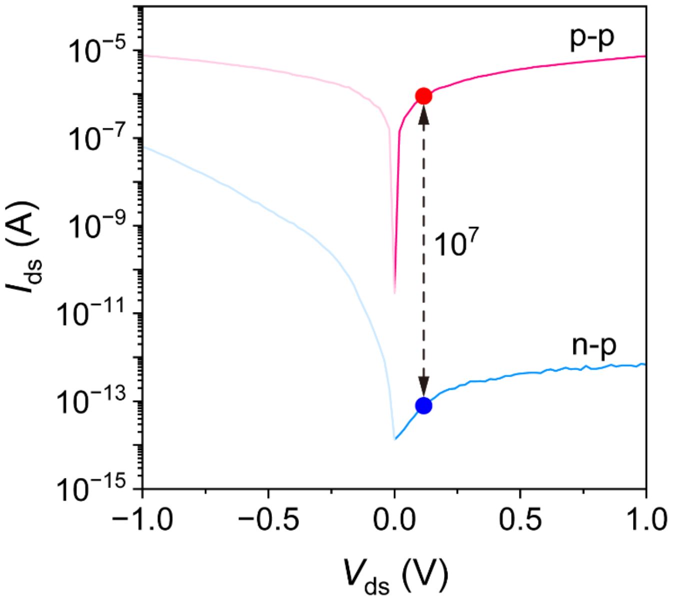

Supplementary Fig. 22| *I-V* curves of the homojunction at n-p (blue) and p-p (pink) states. The dots represent the corresponding currents of the n-p and p-p junctions at V_{ds} = 0.1 V, with an on/off ratio of 10^7.

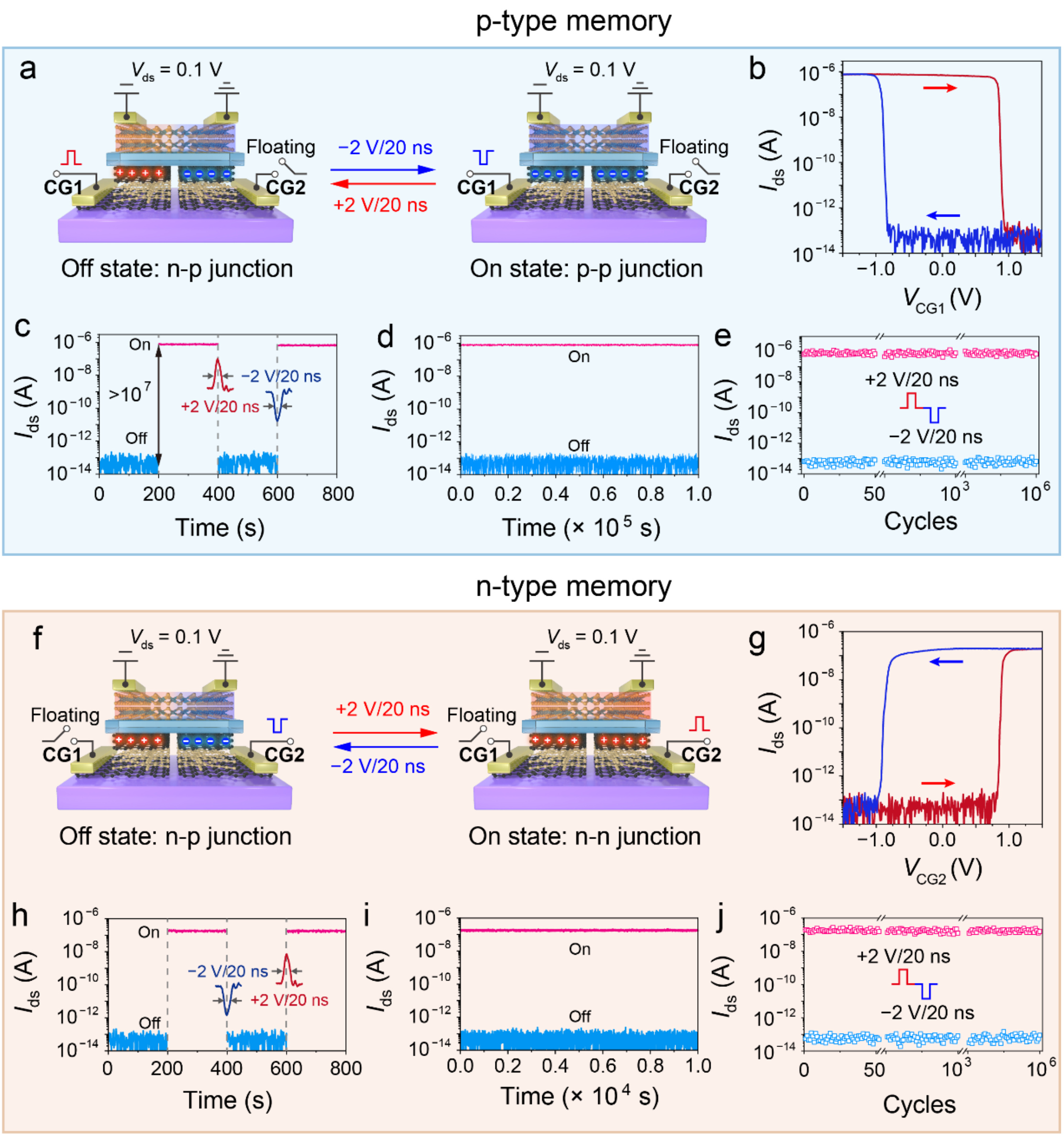

Supplementary Fig. 23| P-type and n-type memory mode of the MM-SFGM. a,**b**,**f**,**g**, illustrations and transfer curves of p-type (**a**,**b**) and n-type (**f**,**g**) memory. For p-type memory, a –2 V/20 ns V_G pulse is first applied to CG2 to inject numerous electrons into the right floating-gate, leading to a heavily p-doped region. After that, the CG2 terminal is floated, and the programming/erasing operation signals are applied to the CG1 terminal. For a positive V_G pulse, holes are injected into the left floating-gate, forming a n-p junction in the WSe_2 channel. The reverse-biased n-p junction leads to an ultralow current (State 0). On the contrary, electrons are injected for a negative V_G pulse, and the whole WSe_2 channel is heavily p-doped, resulting in a high-conductance state (State-1). Similarly, for n-type memory, a +2 V/20 ns V_G pulse is first applied to CG1, and thus the left part of the WSe_2 channel is heavily n-doped. Then the CG1 terminal keeps floating and programming/erasing signals are applied to the CG2 terminal. While applying a negative V_G pulse, the right part of the WSe_2 channel is p-

doped, and the reverse-biased n-p junction results in the ultralow channel current (State-0). On the contrary, a positive V_G pulse will lead to a n-n junction, and thus the device is switched to the high-conductance state (State-1). An obvious memory window with a high on/off ratio is observed in both the p-type and n-type memory, with gate voltage sweeping from –1.5 V to +1.5 V and then back to –1.5 V. **c**,**h**, High-speed programming and erasing operations of the p-type (**c**) and n-type memory (**h**) by applying ±2 V/20 ns V_{CG} pulses to CG1 and CG2, respectively, with an on/off ratio approximately 10^7. **d**,**i**, Retention behaviors of the p-type (**d**) and n-type memory (**i**) at State-0 and State-1, respectively. **e**,**j**, Cyclic endurance of the p-type (**e**) and n-type memory (**j**) showing 10^6 cycles of programming/erasing operations by alternatively applying ±2 V/20 ns V_{CG} pulses.

2.3 Mode-2: Linear and symmetric conductance modulation for analog computing

In applications that demand analog weight updates—such as IMC or neuromorphic computing—transitions from the intrinsic undoped state to either heavily p-doped or n-doped states offer significant advantages. These pathways demonstrate superior linearity and symmetry in conductance modulation, with multiple well-separated intermediate states that enable fine-grained analog representation. As shown in Supplementary Fig. 24, these configurations yield much higher classification accuracy in analog neural network simulations compared to the more binary memory-type states.

The synaptic function of the MM-SFGM is configured by connecting CG1 and CG2 and applying identical programming pulses (±1.2 V, 20 ns) to uniformly update the channel conductance via charge injection into both floating gates (Supplementary Fig. 25). In this operation mode, the entire WSe_2 channel is doped uniformly, and the overall conductance is controlled by the total number of injected charges (Supplementary Fig. 26). Transfer curve measurements (Supplementary Fig. 27) show that the intrinsic, undoped state has an extremely low conductance (~10^{-11} S), while the p- and n-doped states reach ~10^{-5} S and ~10^{-6} S, respectively.

Importantly, conductance states below 0.1 μS are found to be difficult to distinguish due to low signal-to-noise ratio and high nonlinearity, thus limiting their utility in analog computing. As a result, the operational conductance window for IMC is restricted to 0.1–7.0 μS, within which the device shows good linearity and low variability (Supplementary Fig. 28). This range is ideal for representing analog weights in neuromorphic systems, ensuring both accuracy and stability during computation.

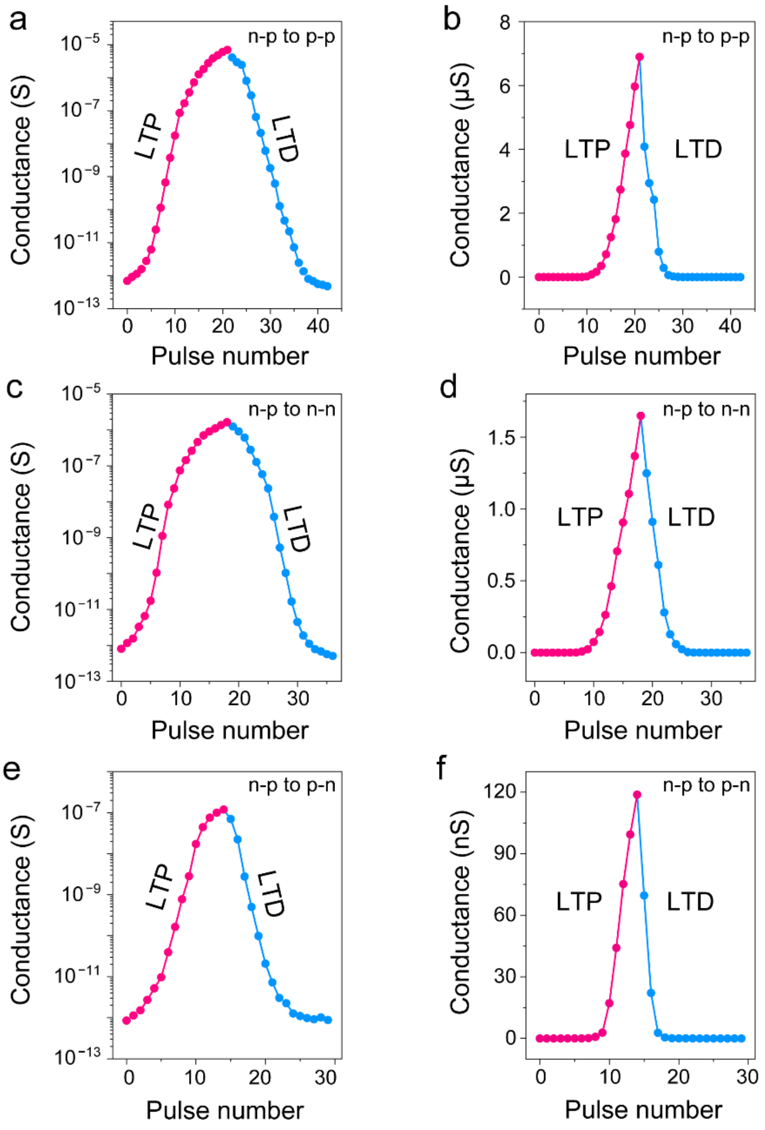

Supplementary Fig. 24| LTP and LTD curves for the device with different conductance updating pathways. a,**b**, LTP (pink) and LTD (blue) curves for the switching pathway between n-p and p-p on a semi-logarithmic scale (**a**) and a linear scale (**b**). **c**,**d**, LTP (pink) and LTD (blue) curves for the switching pathway between n-p and n-n on a semi-logarithmic scale (**c**) and a linear scale (**d**). **e**,**f**, LTP (pink) and LTD (blue) curves for the switching pathway between n-p and p-n on a semi-logarithmic scale (**e**) and a linear scale (**f**).

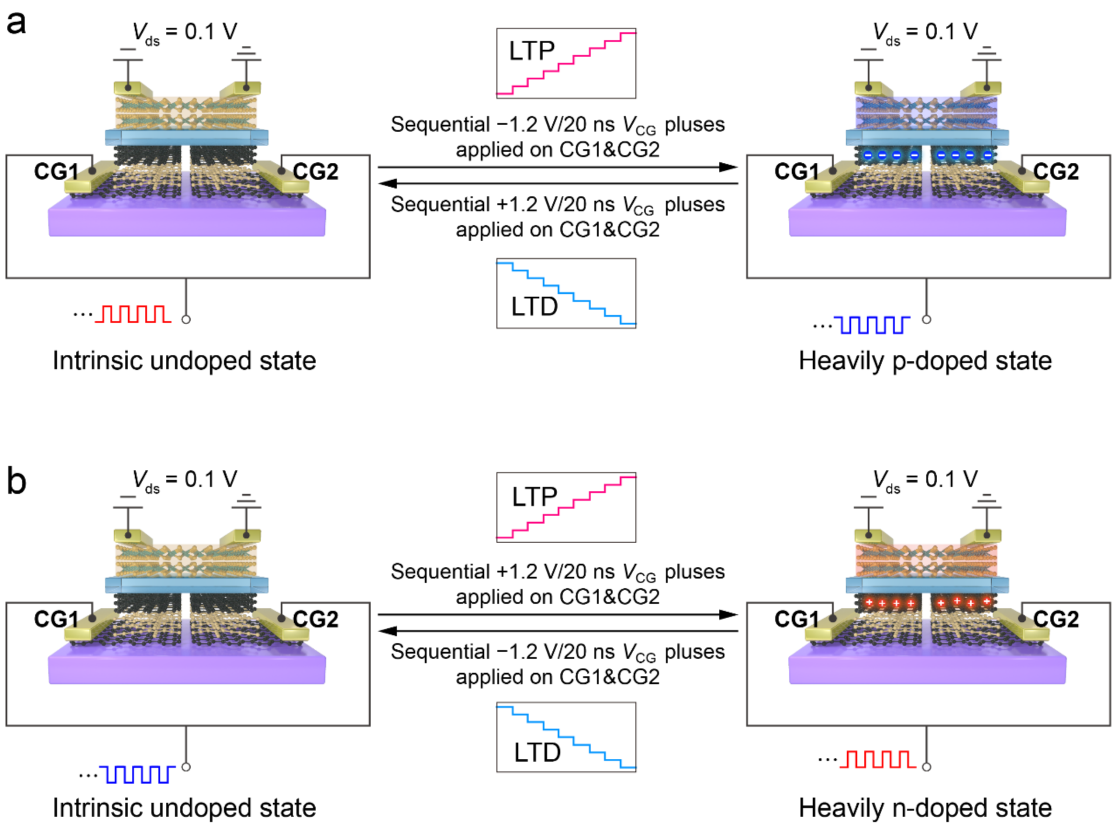

Supplementary Fig. 25| Configurations of the MM-SFGM for synaptic applications. a, Configuration for conductance updating between intrinsic undoped state (low conductance) and heavily p-doped state (high conductance). For LTP, sequential –1.2 V/20 ns V_{CG} pulses are applied simultaneously to both CG1 and CG2. In contrast, sequential +1.2 V/20 ns V_{CG} pulses are applied simultaneously to CG1 and CG2 for LTD. **b**, Configuration for conductance updating between intrinsic undoped state (low conductance) and heavily n-doped state (high conductance). Sequential +1.2 V/20 ns V_{CG} pulses are applied to CG1 and CG2 for LTP, and sequential –1.2 V/20 ns V_{CG} pulses are applied for LTD.

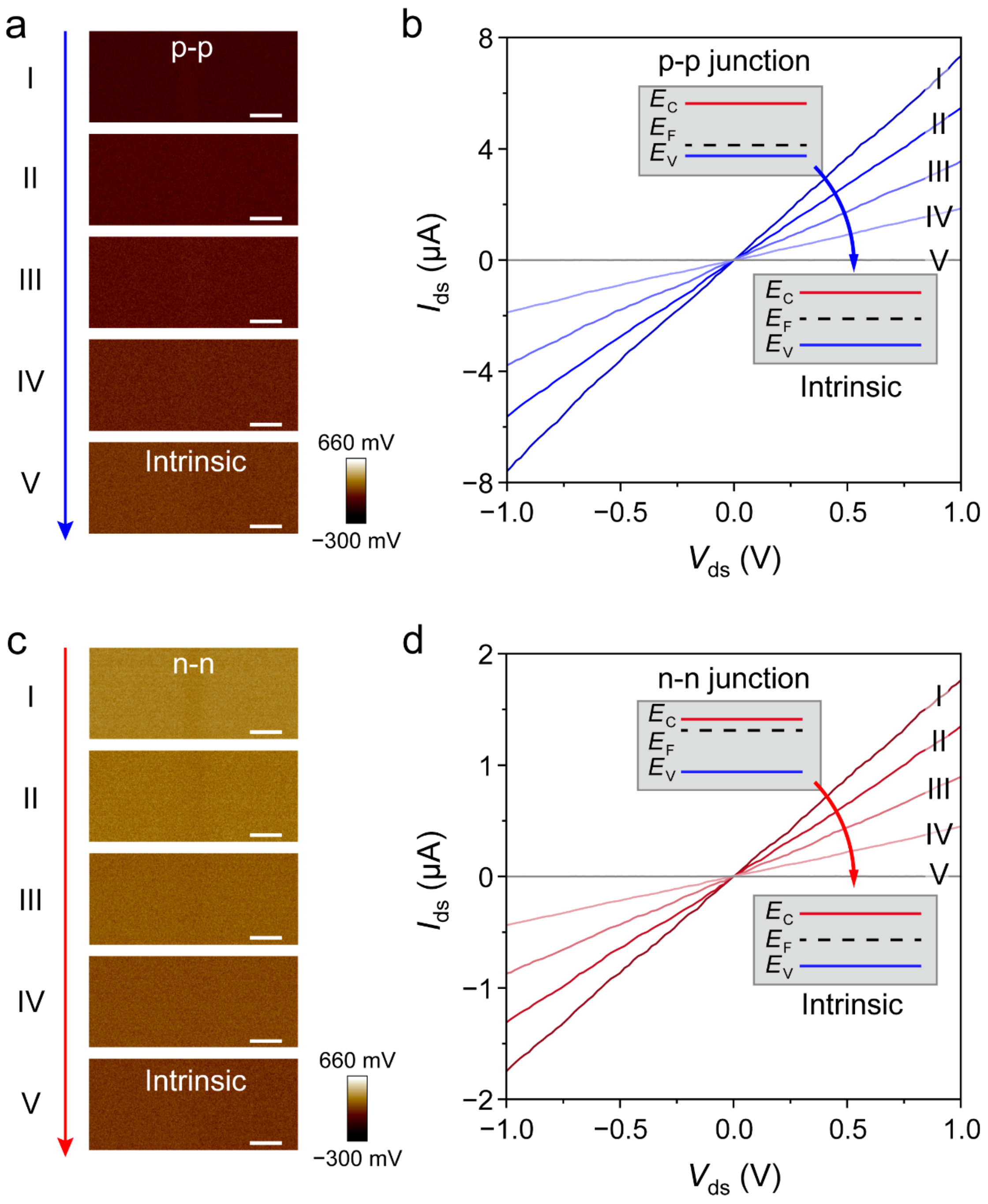

Supplementary Fig. 26| Dynamic modulation of the homojunction between p-p and n-n states and their conductance. a, KPFM images of the intermediate junction states from p-p junction (state I) to intrinsic undoped state (state V). **b**, *I*-*V* curves of the device at intermediate states tunning from p-p state to intrinsic undoped state. **c**, KPFM images of the intermediate junction states from n-n junction (state I) to intrinsic undoped state (state V). **d**, *I*-*V* curves of the device at intermediate states tunning from n-n state to intrinsic undoped state. The conductance of the device can be dynamically modulated in a wide range by controlling the doping levels of the channel.

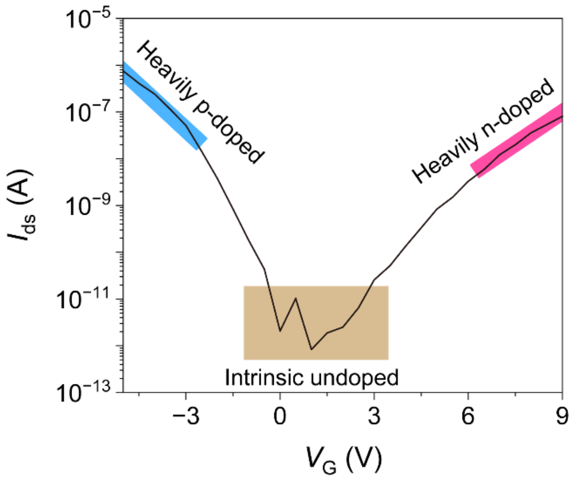

Supplementary Fig. 27| Transfer curve of the ambipolar WSe_2 channel. The curve shows relatively symmetric p-type and n-type branches, enabling the conductance updating between the intrinsic undoped state and either the heavily p-doped or n-doped states. The read voltage (V_{ds}) was set to 0.1 V.

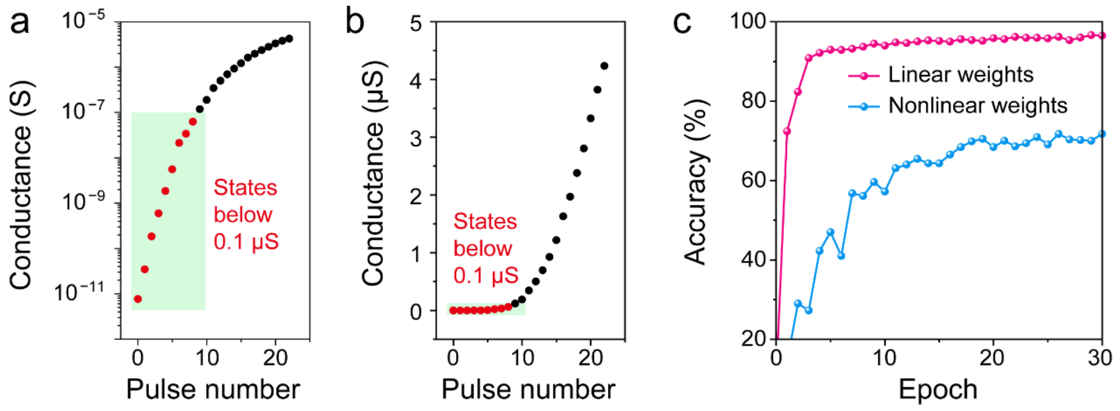

Supplementary Fig. 28| Conductance updating behaviors for the conductance states below 0.1 μS. a,**b**, Conductance states for updating in range of 10^{-11} S to 10^{-5} S on a semi-logarithmic scale (**a**) and a linear scale (**b**). **c**, Classification accuracies of an ANN with linear (pink) and nonlinear (blue) weights for the MNIST dataset.

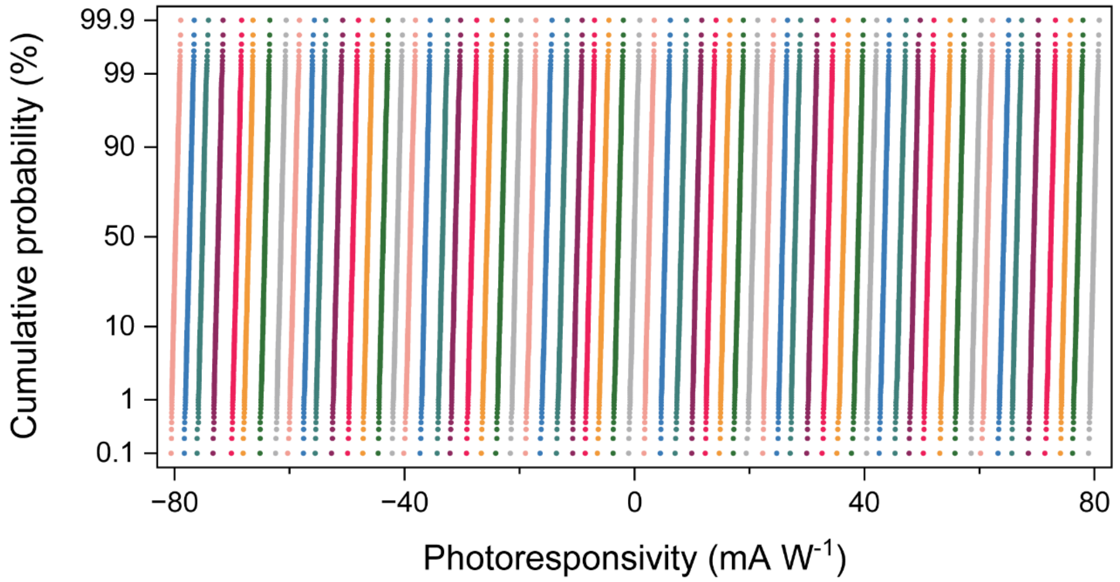

Supplementary Fig. 29| Cumulative probability distribution of the MM-SFGM with respect to 63 discrete photoresponsivity states for 1000 repetitions.

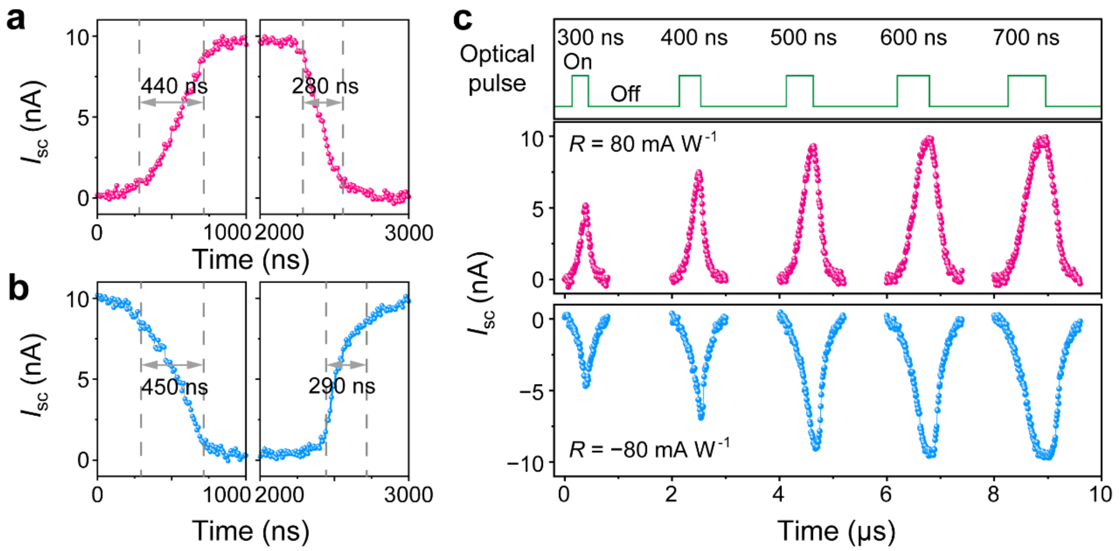

Supplementary Fig. 30| Nanosecond operation speed of the MM-SFGM in sensor mode for high-speed ISC. a,**b**, The rise and fall edges of the device with a positive photoresponsivity (**a**) and a negative photoresponsivity (**b**). The rise and fall time are approximately 450 ns and 290 ns, respectively. **c**, Photoresponse of the MM-SFMG with positive (pink) and negative (blue) photoresponsivity to nanosecond-order optical pulses. Optical pulses with widths of 300 ns, 400 ns, 500 ns, 600 ns, and 700 ns were applied on the device, and the photocurrents generated at the short-circuit condition were measured for the device with photoresponsivity of ±80 mA W^{-1}.

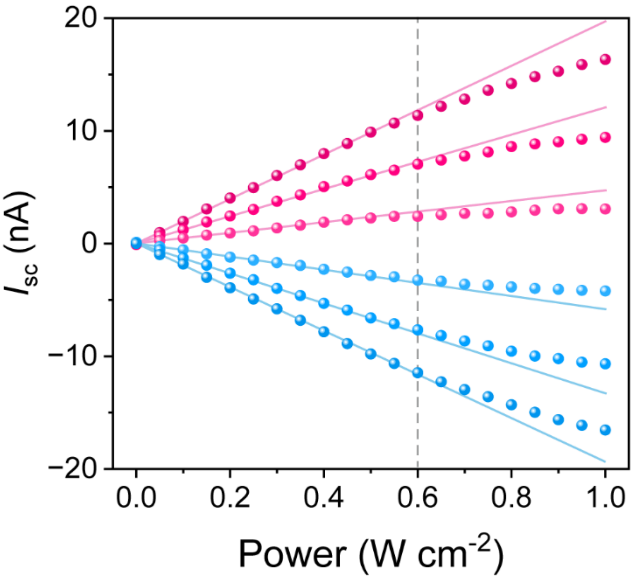

Supplementary Fig. 31| Light-intensity dependence of the photocurrents for 6 photoresponsivity states. The photocurrents exhibit linear dependence on the light intensity in range of 0–0.6 W cm^{-2}, and gradually deviate from linearity as the power exceeds 0.6 W cm^{-2}.

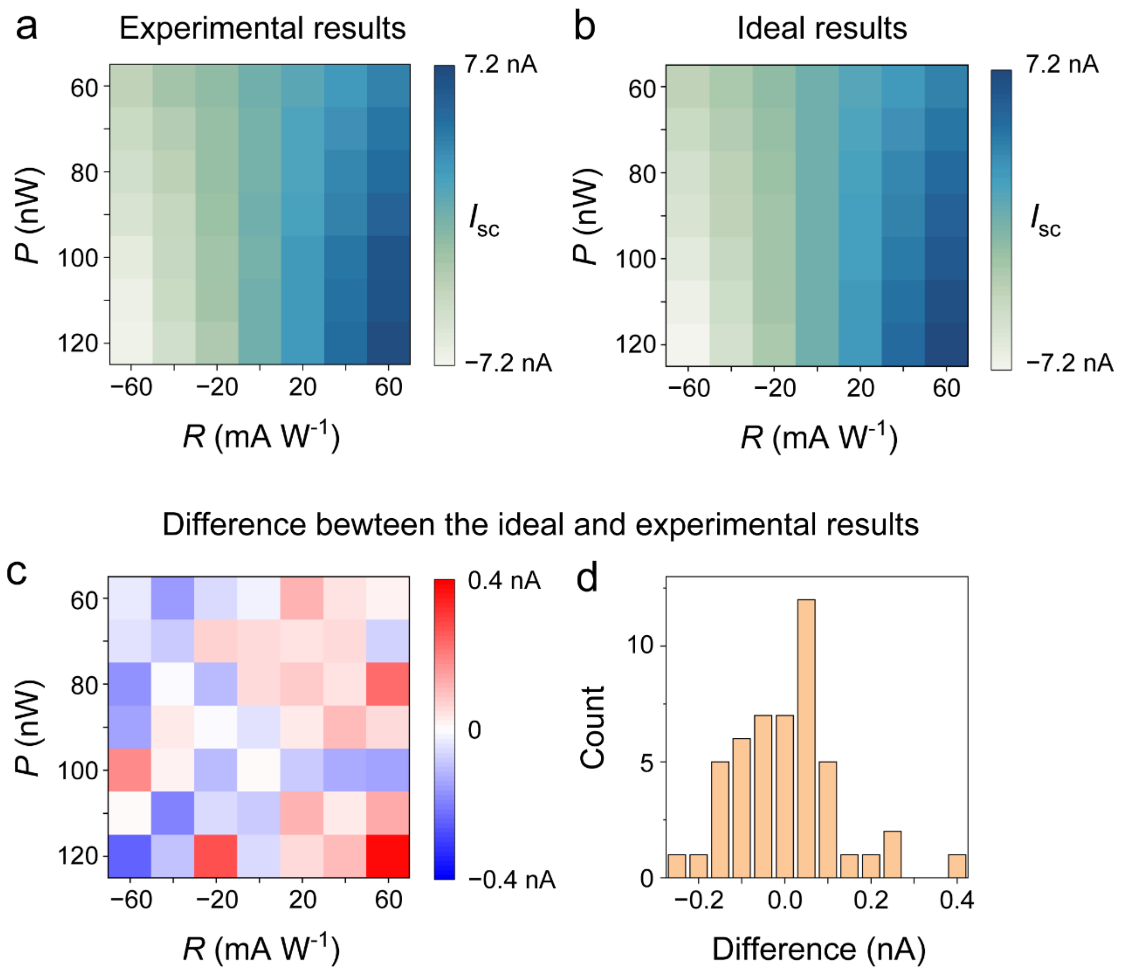

Supplementary Fig. 32| Analog-analog multiplications between different pairs of light intensity and photoresponsivity. a,**b**, Experimental output photocurrents (**a**) and ideal results (**b**) for the analog-analog multiplications. The horizontal and vertical axes correspond to light intensity and photoresponsivity, respectively. **c**, Current differences between the experimental and ideal results for each combination. **d**, Statistical distribution of the current differences as presented in **c**.

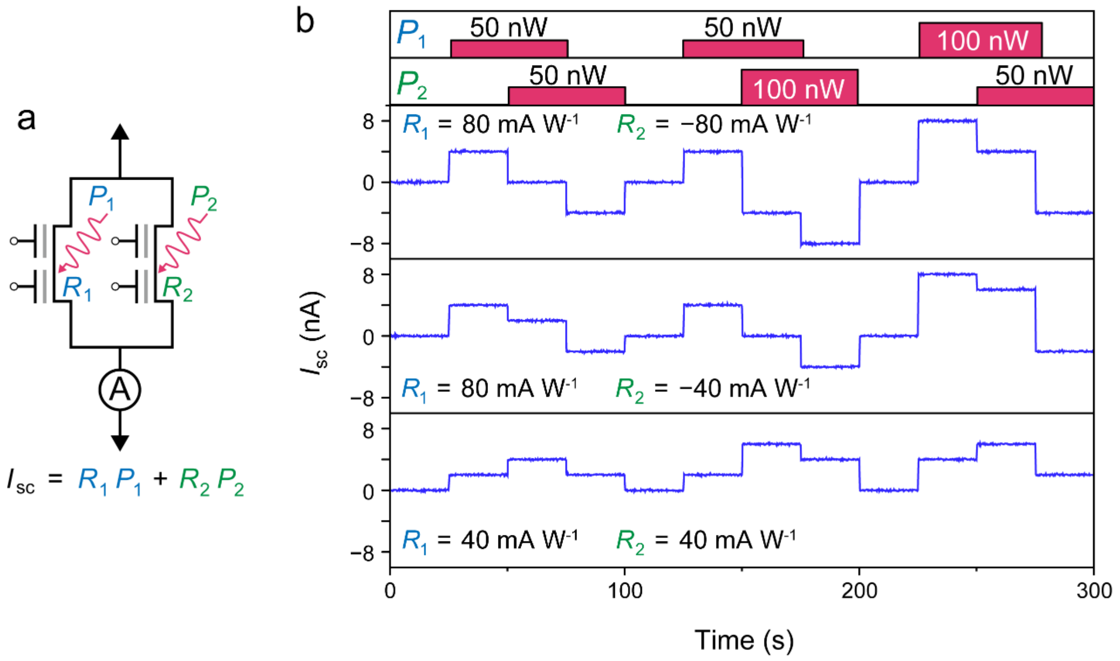

Supplementary Fig. 33| Accumulation of photocurrents generated by two parallel MM-SFGMs. a, Illustration of the circuit for the two parallel sensor-mode MM-SFGMs. Two devices are programmed to p-n or n-p states with photoresponsivity of R_1 and R_2, respectively. The effective light intensities illuminated on the two devices are P_1 and P_2, respectively. The sensor-mode MM-SFGMs operate in the short-circuit condition, and I_{sc} represents the measured total photocurrent generated by the two parallel devices. **b**, The output total photocurrents generated by the two devices while illuminating with different P_1 and P_2 combinations. R_1 and R_2 are set as 80/80/40 mA W^{-1} and –80/–40/40 mA W^{-1}, respectively. The measured I_{sc} follows $I_{sc} = R_1P_1 + R_2P_2$, demonstrating that the generated positive and negative photocurrents can accumulate directly without subtracting dark currents.

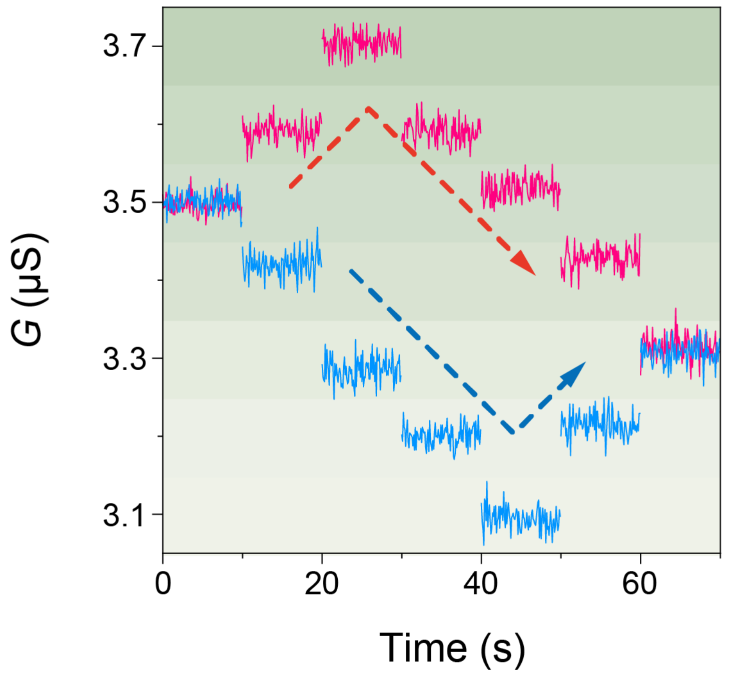

Supplementary Fig. 34| Precise modulation of the conductance between various levels by applying corresponding numbers of paired V_G pulses to the split control-gates.

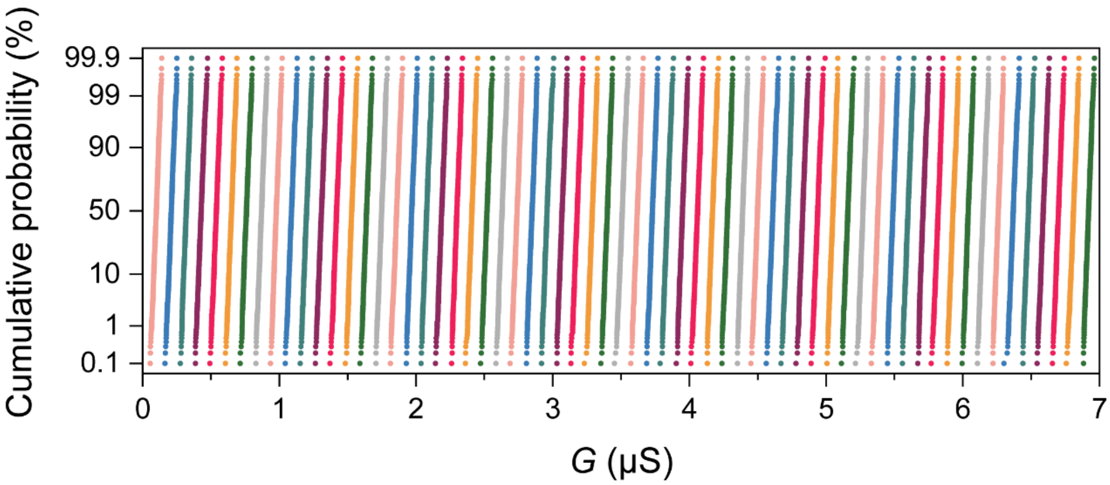

Supplementary Fig. 35| Cumulative probability distribution of the MM-SFGM with respect to 63 discrete conductance states for 1000 repetitions.

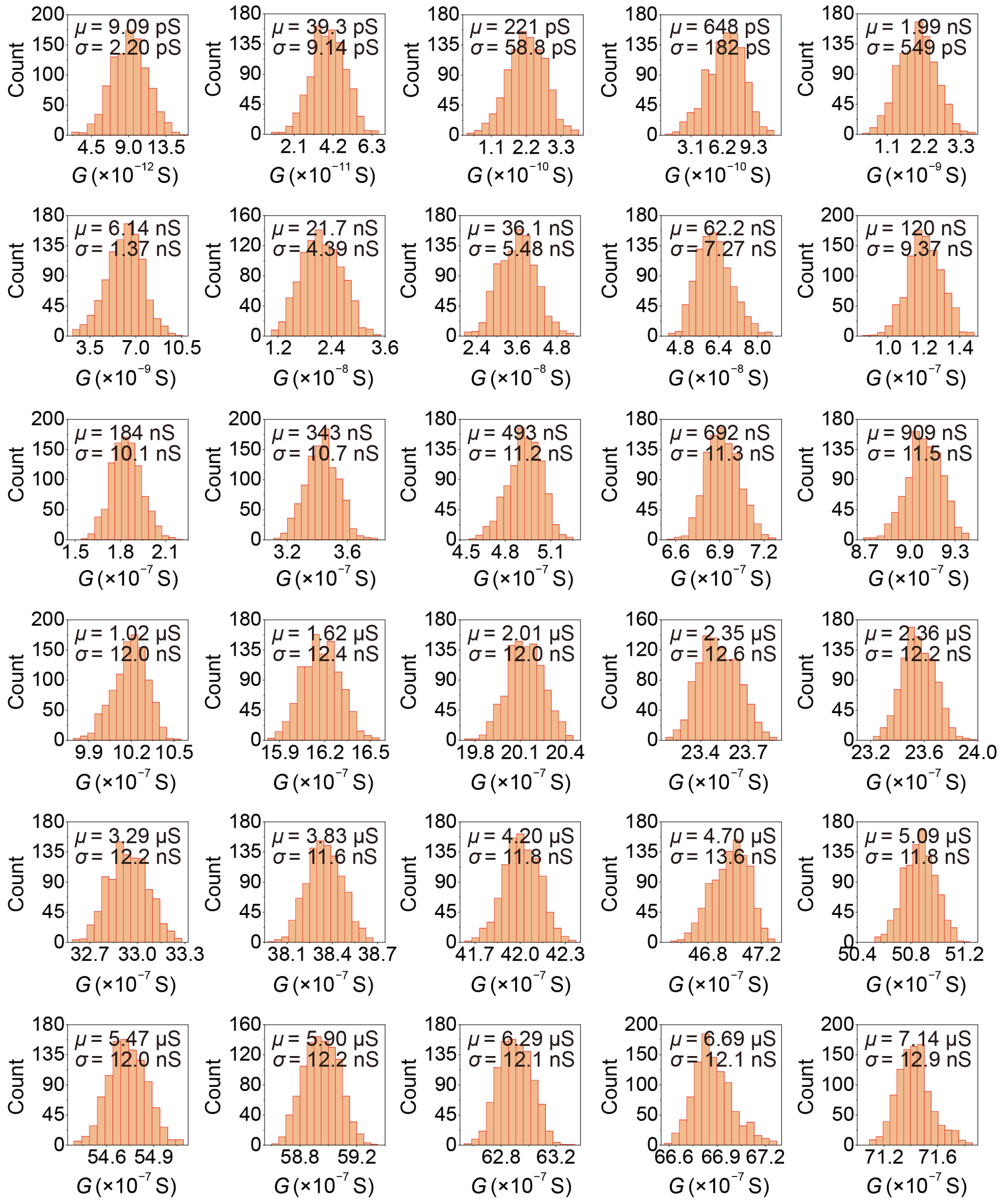

Supplementary Fig. 36| Distribution of measured conductance for 30 individual states. For each programmed conductance state across the full range (10 pS to 7 μS), 10 independent programming cycles were performed. After each programming cycle, the conductance was read 100 consecutive times, resulting in 1000 measured values per state. The histogram shows the distribution of these 1000 readout values for each conductance state. These measurement results serve as the basis for the error analysis presented in **Fig. 3e**.

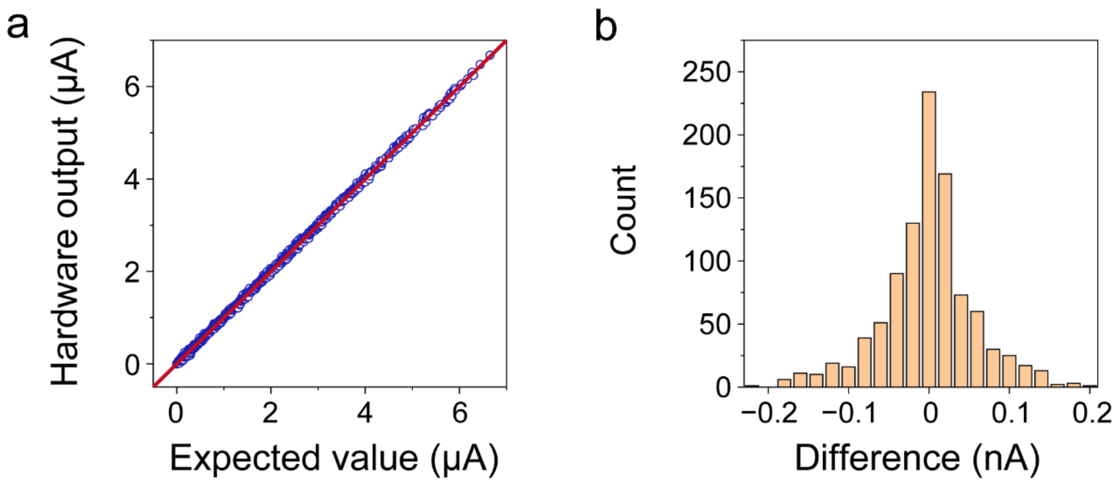

Supplementary Fig. 37| Statistics of the analog-analog multiplications for 1000 randomly generated input voltages and conductance combinations. a, Linear fitting between the hardware output currents and expected values of the analog-analog multiplications. **b**, Statistical distribution of the current differences between experimental and ideal results.

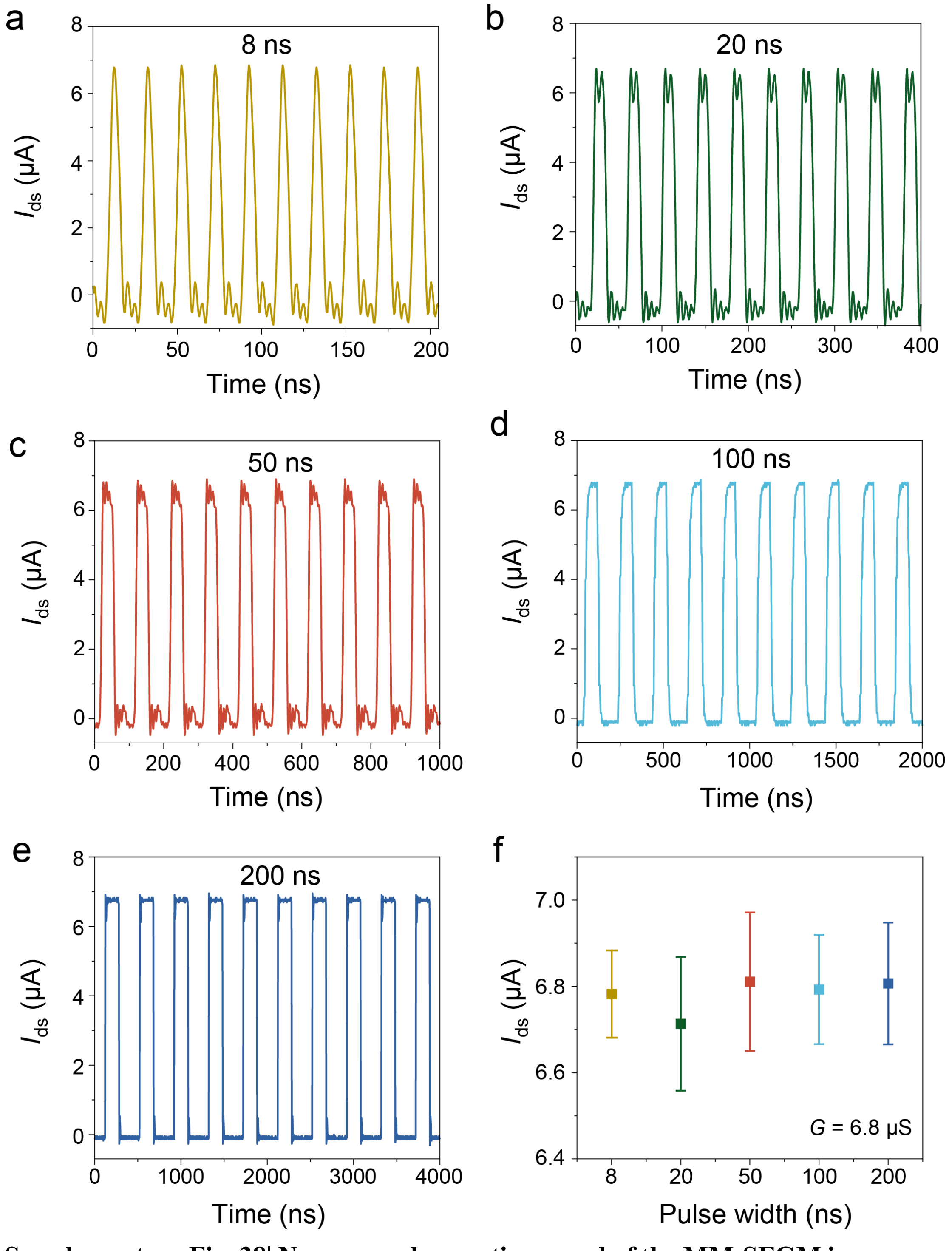

Supplementary Fig. 38| Nanosecond operation speed of the MM-SFGM in synapse mode for high-speed IMC. a–e, Response of the synapse to pulsed input voltage signals with pulse widths of 8 ns (**a**), 20 ns (**b**), 50 ns (**c**), 100 ns (**d**), and 200 ns (**e**), respectively. **f**. Statistics of the output currents of the synapse for different input pulse widths. The conductance is set to 6.8 μS. Error bars denote the standard deviation from 10 measurements.

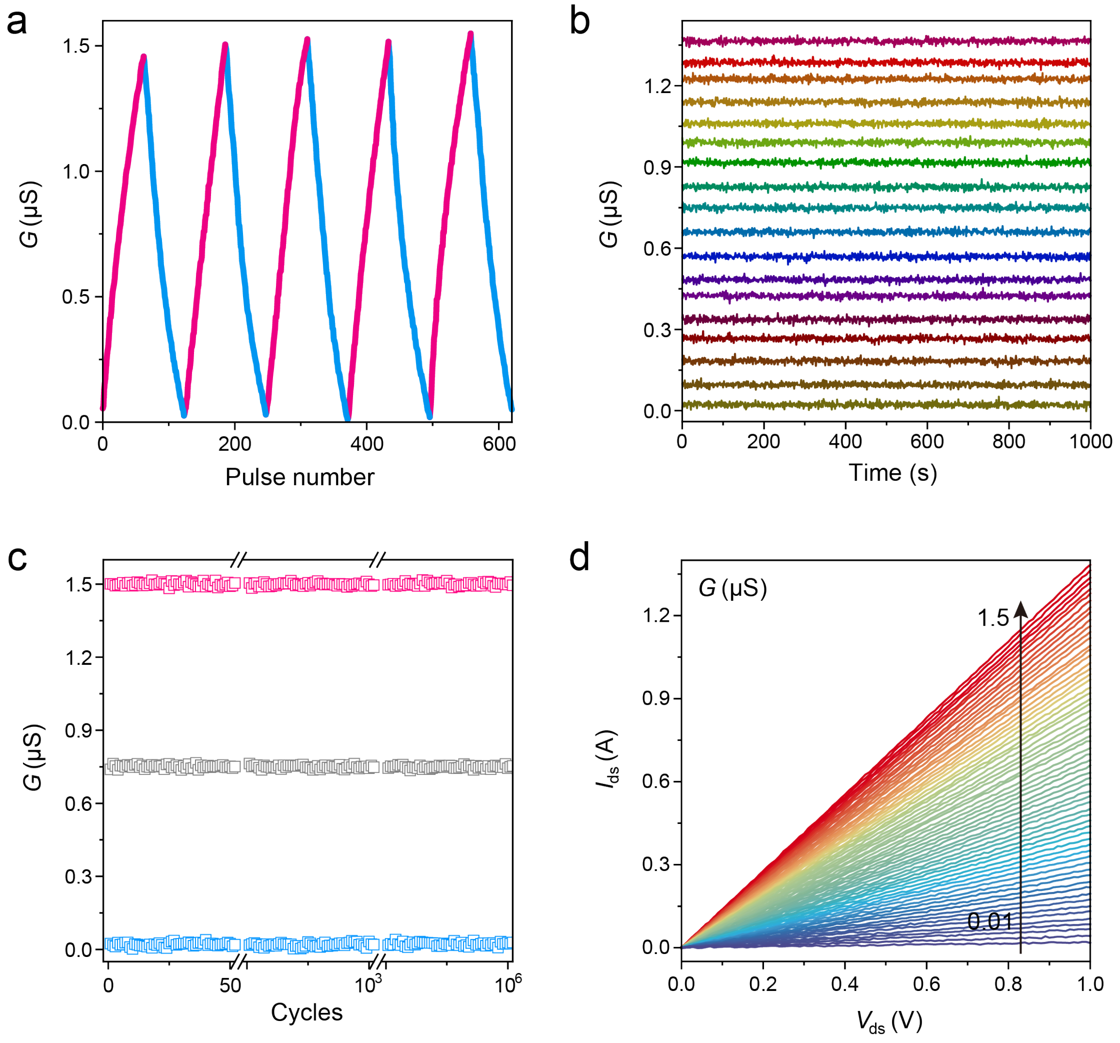

Supplementary Fig. 39| Conductance update behaviors of the MM-SFGM with n-n junction for IMC. a, Conductance update behaviors of the device in range of 0–1.5 μS by successively applying +1 V/20 ns (LTP) and –1 V/ 20 ns (LTD) V_G pulses. **b**, Long-term retention of the conductance at different states. **c**, Cyclic endurance of the device by applying switching V_G pulses for 10^6 times. **d**, *I-V* curves of the device with different conductance states.

Supplementary Note 3. Nonlinear activation functions based on rectification characteristics of MM-SFGM

3.1 Device-level realization of ReLU and Sigmoidal NAFs

The rectification behavior of the MM-SFGM in the p-n state enables the implementation of nonlinear activation functions (NAFs). As illustrated in Supplementary Fig. 40, the device's *I–V* curve can be divided into three distinct regions:

- $V_{ds} < 0$: The output current I_{ds} tends to zero (approximately 10^{-12} A) due to the diode-like blocking of reverse bias.
- $0 < V_{ds} < V_T$: The output current I_{ds} follows an exponential dependence as described by the Shockley equation, marking the nonlinear regime. V_T is defined as the transition voltage from nonlinear to linear regions.
- $V_{ds} > V_T$: The *I–V* curve transitions into a linear region, enabling gradient-preserving operations.

The position of the transition voltage V_T and the slope of the linear region are tunable through independent modulation of the electron and hole densities stored in the split floating gates (Supplementary Fig. 41,42). Low gate charge densities reduce the built-in potential V_{bi}, leading to a lower threshold voltage and broader linear region—ideal for ReLU. In contrast, higher gate charge densities increase V_{bi}, sharpening the rectification behavior and enabling Sigmoid-like responses (see Fig. 4c–d in the main text).

For ReLU implementation, a voltage offset is applied (i.e., $V_{ds} = V_{in} + V_{offset}$) to align the turn-on threshold with zero input. Sigmoid-type activation is implemented by applying a compliance current (I_{CC}) limit to enhance exponential suppression at low input voltages (Supplementary Fig. 43,44). The activation region is confined within ±1 V to prevent floating-gate perturbation.

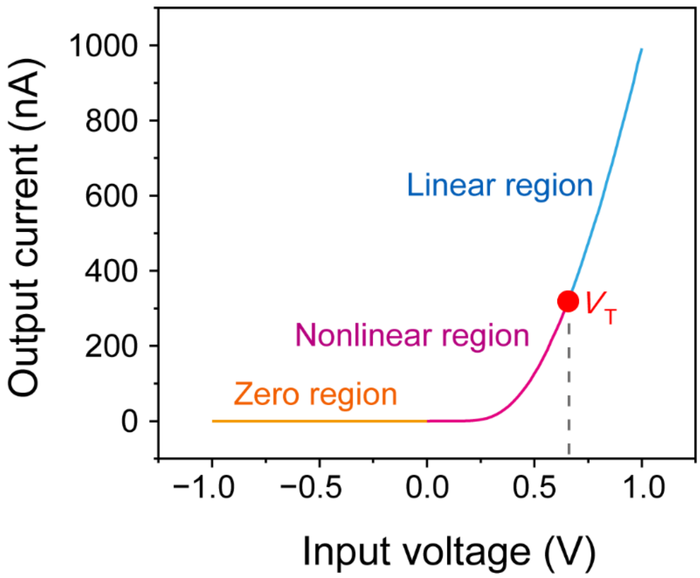

Supplementary Fig. 40| Typical *I*–*V* curve of the MM-SFGM in the p-n state. The rectifying curve can be divided into three distinct regions: zero-current region for $V_{ds} < 0$, nonlinear region for $0 < V_{ds} < V_T$, and linear region for $V_{ds} > V_T$, where V_T represents the voltage that the *I*–*V* curve transition from nonlinear to linear.

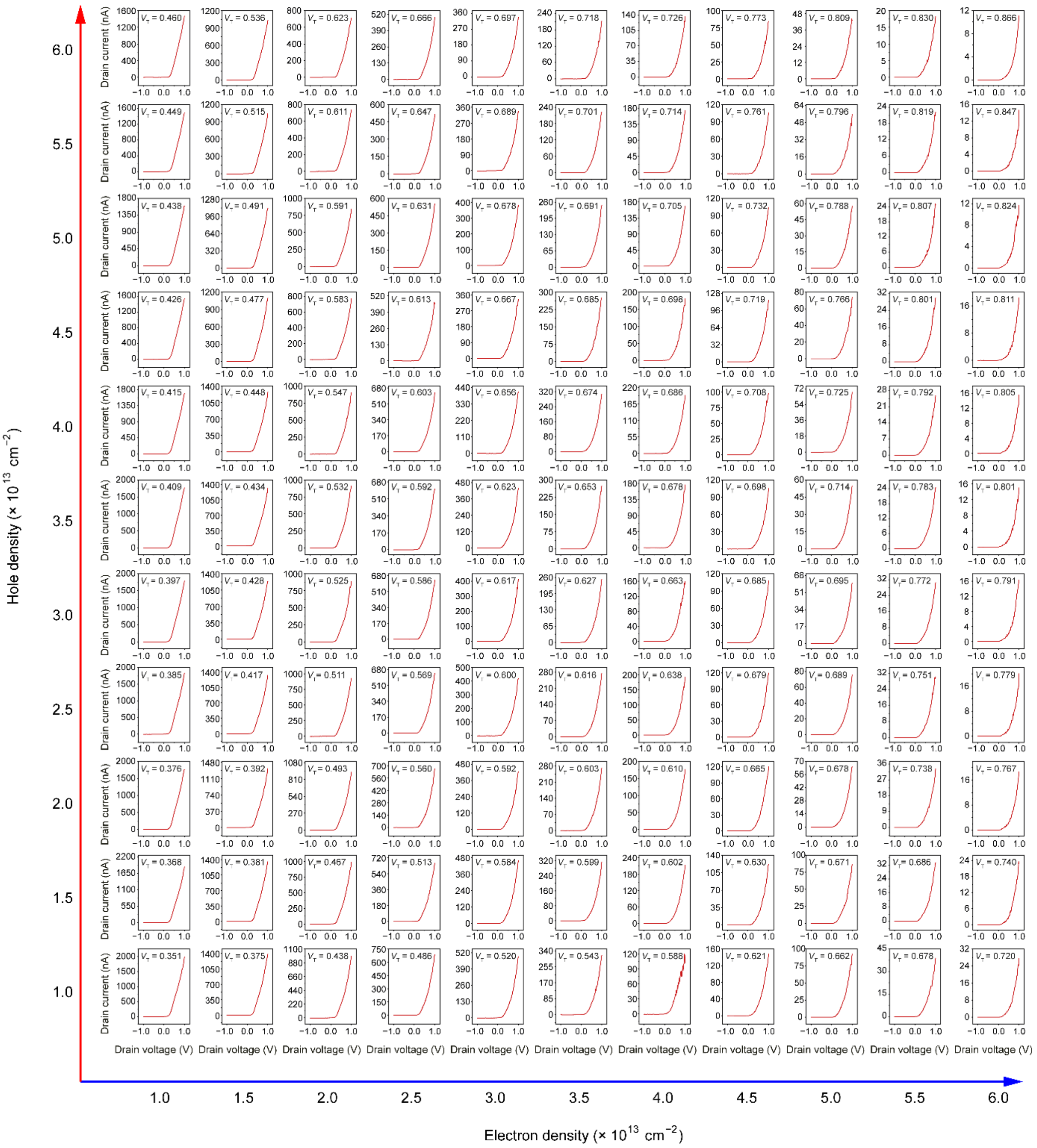

Supplementary Fig. 41| *I*–*V* curves of the MM-SFGM with varying electron and hole densities in the split floating-gates. The horizontal direction represents the modulation of electron density in the left floating-gate, while the vertical direction represents the variation in hole density in the right floating-gate.

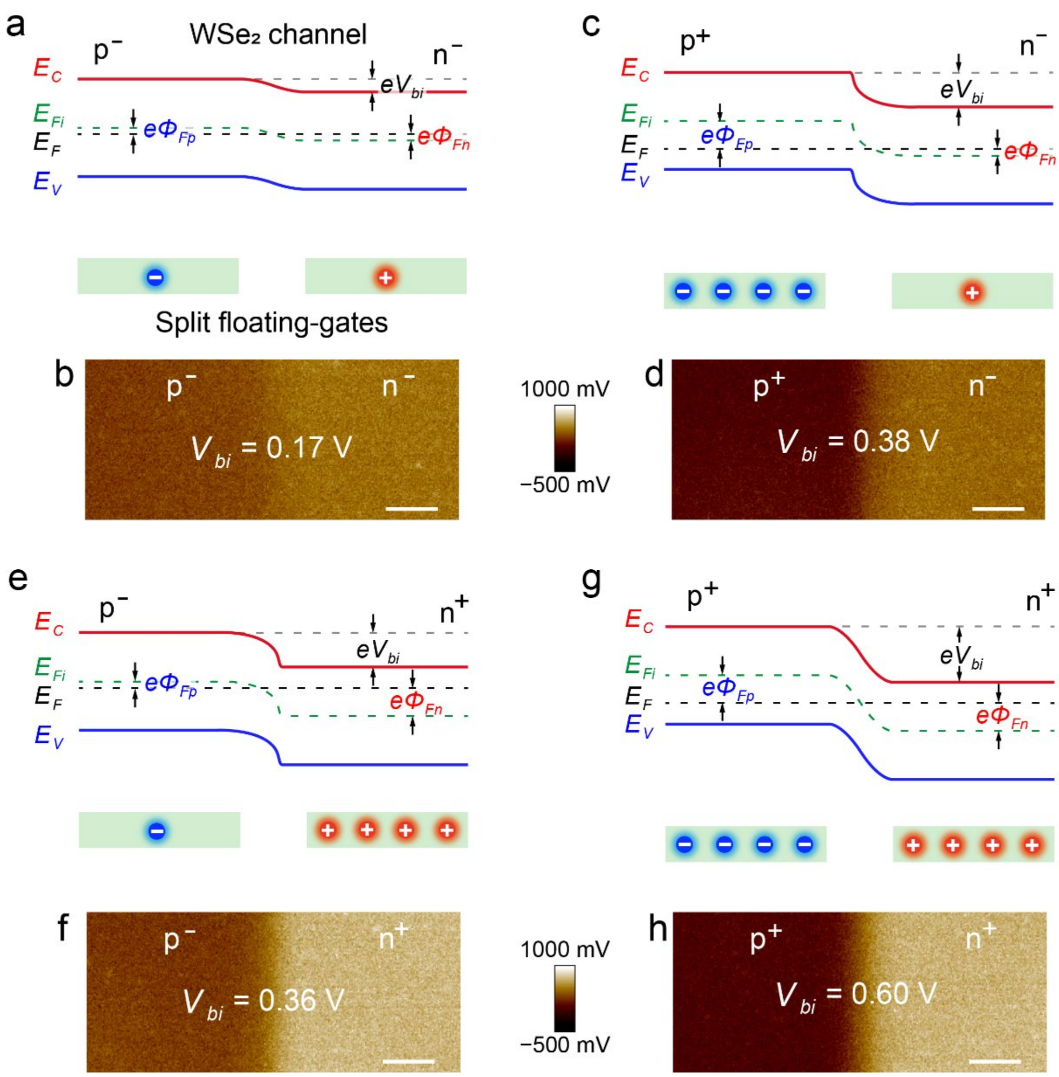

Supplementary Fig. 42| Band structures and KPFM images of the WSe_2 channel with tunable built-in potential. a,**c**,**e**,**g**, Band alignments of the WSe_2 channel in p^--n^- (**a**), p^+-n^- (**c**), p^--n^+ (**e**), and p^+-n^+ (**g**) states, where p^- and p^+ represent slightly and heavily p-doped WSe_2, and n^- and n^+ represent slightly and heavily n-doped WSe_2, respectively. The doping levels of WSe_2 channel are controlled by the concentrations of electrons and holes stored in the corresponding floating-gates. E_C and E_V represent the conduction and valence band energies, respectively, while E_F and E_{Fi} correspond to the Fermi level and intrinsic energy level, respectively. ϕ_{Fp} and ϕ_{Fn} refer to the electric potentials between E_F and E_{Fi} in the p-type and n-type channels, respectively. The built-in potential $V_{bi} = |\phi_{Fp}| + |\phi_{Fn}|$. **b**,**d**,**f**,**h**, KPFM images of the WSe_2 channel in p^--n^- (**b**), p^+-n^- (**d**), p^--n^+ (**f**), and p^+-n^+ (**h**) states. Scale bars, 1 μm.

a

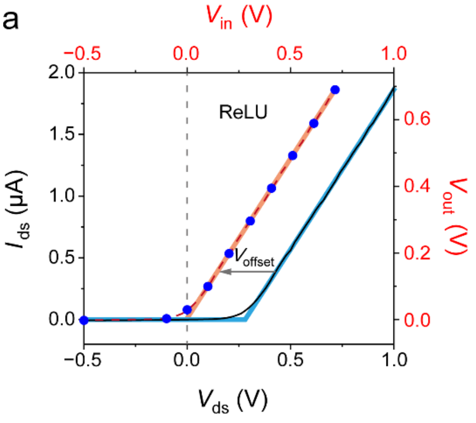

b

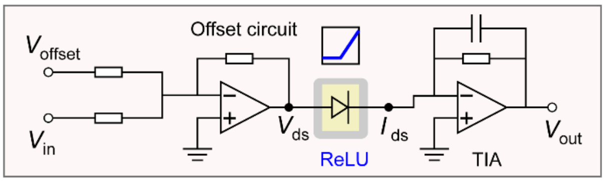

c

V_{in} (V)	−0.5	−0.1	0.0	0.1	0.2	0.3	0.4	0.5	0.6	0.7
V_{offset}	0.3 V									
V_{ds} (V)	−0.2	0.2	0.3	0.4	0.5	0.6	0.7	0.8	0.9	1.0
I_{ds} (μA)	0.00	0.01	0.09	0.30	0.56	0.82	1.07	1.34	1.61	1.88
V_{out} (V)	0.00	0.00	0.03	0.11	0.21	0.30	0.40	0.49	0.59	0.69

Supplementary Fig. 43| Configuration of the MM-SFGM for ReLU. a, *I*–*V* curve of the device showing a large linear region (small V_T) suitable for ReLU. The linear region of the blue curve can be expressed as $I_{ds} = a * V_{in} - b$, where a and b refer to the slope and *y*-axis intercept, respectively. By adding an offset voltage V_{offset} to V_{in}, the linear region can be horizontally shifted to start from 0 V (red curve), resulting in $I_{ds} = a * V_{in}$ with $a * V_{offset} = b$. I_{ds} is then amplified and converted into a voltage signal (V_{out}) by a transimpedance amplifier (TIA). **b**, Schematic of the circuit used for ReLU operation. An offset circuit is employed to add V_{offset} to V_{in}, which serves as the input voltage (V_{ds}) for the ReLU activator, and a TIA circuit is used to amplify and convert I_{ds} to V_{out}. **c**, Experimental data obtained from the ReLU circuit as shown in **b**, demonstrating the successful implementation of the hardware ReLU operation.

a

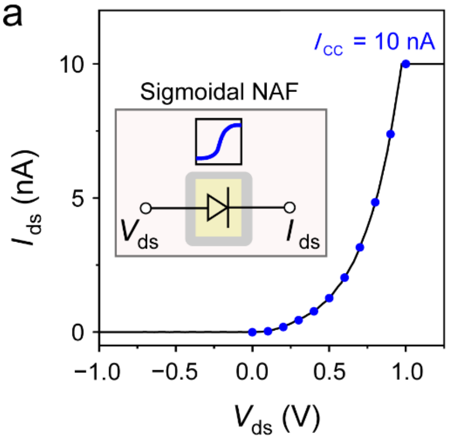

b

V_{in} (V)	V_{ds} (V)	I_{ds} (nA)
0.0	0.0	0.0
0.1	0.1	0.0
0.2	0.2	0.2
0.3	0.3	0.5
0.4	0.4	0.8
0.5	0.5	1.3
0.6	0.6	2.0
0.7	0.7	3.1
0.8	0.8	4.8
0.9	0.9	7.4
1.0	1.0	10.0

Supplementary Fig. 44| Configuration of the MM-SFGM for Sigmoidal NAF. a, *I*–*V* curve of the device showing a large nonlinear region (large V_T) suitable for Sigmoidal NAF. The compliance current (I_{CC}) is set to 10 nA. Inset illustrates the circuit for Sigmoidal NAF. **b**, Experimental data obtained from the Sigmoidal NAF hardware.

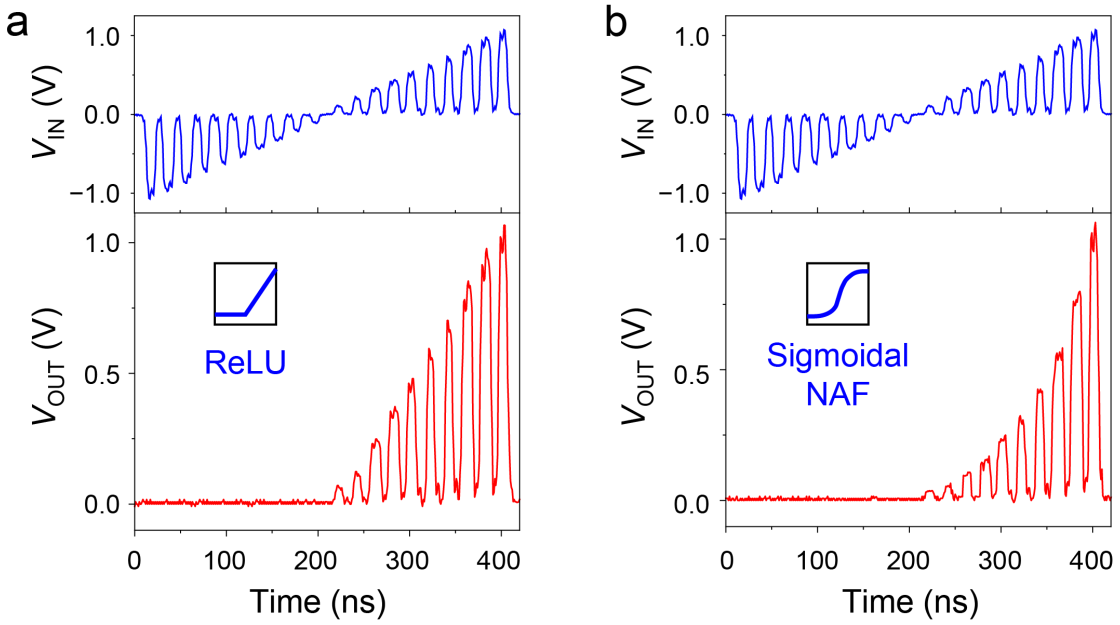

Supplementary Fig. 45| Nanosecond operation speed of the MM-SFGM in neuron mode for high-speed NAFs. a,**b**, Response of a ReLU-type (**a**) and a Sigmoidal-type (**b**) NAF device to 8-ns width pulsed input voltage signals ranging from –1 V to +1 V.

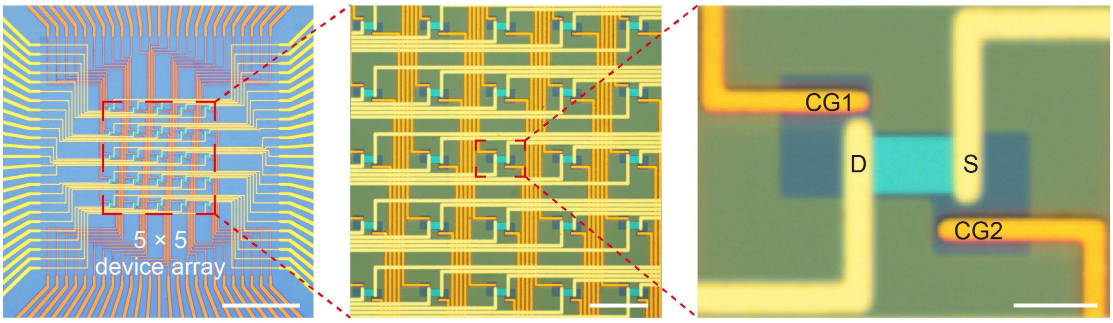

Supplementary Fig. 46| Optical images of a 5 × 5 device array for NAFs. Scale bars (from left to right), 100 μm, 30 μm, and 5 μm, respectively.

3.2 Quantitative evaluation and benchmarking

To rigorously assess the fidelity of MM-SFGM–based NAFs, we quantitatively evaluated how closely the device's *I–V* characteristics match their ideal mathematical forms for ReLU and Sigmoid functions. Two commonly adopted metrics—Root Mean Square Error (RMSE) and Signal-to-Noise Ratio (SNR)—were employed.

As shown in Supplementary Fig. 47, the experimental *I–V* responses of MM-SFGM devices configured for ReLU and Sigmoidal modes were compared to their respective theoretical functions, and the RMSE values were calculated as 0.006 (for ReLU) and 0.03 (for Sigmoid), respectively. These low RMSE values confirm that the device exhibits excellent agreement with the expected activation functions. The SNR values were further calculated as ~35 dB (for ReLU) and ~24 dB (for Sigmoid), using the formula:

$$\mathrm{SNR(dB)} = 10 \bullet \log_{10}\left(\frac{\sum y_{\mathrm{true}}^2}{\sum\left(y_{\exp} - y_{\mathrm{true}}\right)^2}\right),$$

where y_{true} denotes the ideal ReLU or Sigmoid response, and $y_{\exp}$ represents the experimental device output. These SNR values indicate that the signal quality is substantially higher than noise or distortion, suggesting that the device can robustly support accurate activation operations even in the presence of device-level non-idealities.

Additionally, we evaluated the linearity of the ReLU-mode response using the coefficient of determination (R^2). A value of $R^2 = 0.9948$ was obtained, demonstrating near-ideal linear behavior in the post-threshold region. This compares favorably with or surpasses many state-of-the-art analog ReLU circuits based on CMOS, FLASH, or memristive devices (Supplementary Table 2).

In terms of dynamic range, the MM-SFGM exhibits a continuously tunable output current spanning from 11 nA to 1.9 μA (for $V_{\mathrm{ds}} = 1$ V), enabled by precise control of the stored charge in the split floating gates. This wide and adjustable range provides sufficient coverage to emulate both ReLU-type and Sigmoid-type functions. While other platforms such as CMOS or flash-based circuits may utilize voltage or current

outputs with different scaling characteristics, the broad dynamic range of MM-SFGM ensures robust functional coverage across diverse nonlinear activation tasks, making it highly versatile for integration into various neuromorphic computing layers.

Moreover, the energy consumption per activation is exceptionally low: approximately 16 fJ for ReLU and just ~80 aJ for Sigmoid. These values are orders of magnitude lower than those of digital processors, analog CMOS circuits, and optical ReLU implementations.

These results underscore that MM-SFGM–based NAFs combine high linearity, low error, broad dynamic range, and ultralow energy operation, offering a compact and reconfigurable alternative for energy-efficient neuromorphic hardware.

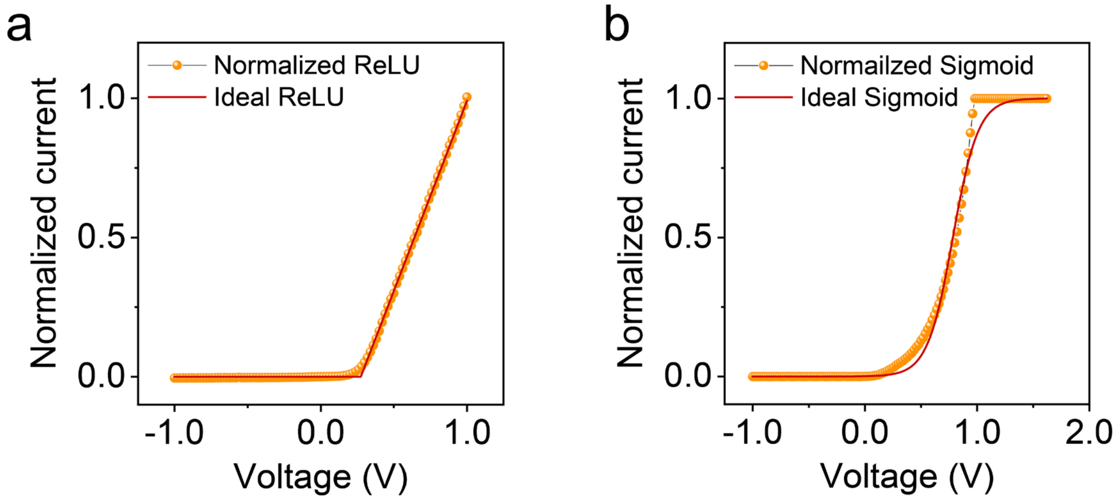

Supplementary Fig. 47| Comparison between measured and ideal nonlinear activation functions. a, Normalized *I–V* characteristics of the MM-SFGM device configured in ReLU mode, overlaid with the ideal ReLU function. **b**, Normalized *I–V* characteristics of the same device configured in Sigmoid mode, overlaid with the ideal Sigmoid function. Offsets were applied to the ideal functions (0.27 V for ReLU and 0.78 V for Sigmoid) to enable shape comparison between the theoretical curves and the experimental response. The strong agreement confirms that the MM-SFGM device can accurately reproduce both ReLU- and Sigmoid-like nonlinear activation behaviors. Quantitative metrics including RMSE and SNR are discussed in Supplementary Note 3.2.

Supplementary Table 2. Comparison of hardware-based ReLU activation implementations across different platforms

Type	Function	Linearity (R^2)	Dynamic range	Power consumption	Ref
Memristor	L-ReLU	0.9887	-200-800 Hz	N/A	7
CMOS	ReLU	0.9958	N/A	N/A	8
CMOS	ReLU	0.9889	-0.1-1.2 V	N/A	9
CMOS	ReLU	0.9988	0-1 V	N/A	10
CMOS	ReLU	0.9966	600-700 mV	14.4 μW	11
MOSFET	ReLU	0.9888	0-0.5 V	10 μW	12
MOSFET	ReLU	0.9860	0-500 nA	N/A	13
FLASH	ReLU	0.9776	0-1.2 V	N/A	14
FLASH	ReLU	0.9913	10 pA-0.1 μA	5.3 pJ	15
All-optical Rectified Linear Unit	ReLU	0.9895	N/A	270 fJ	16
Memory	ReLU	0.9948	11 nA-1.9 μA	4.8 fJ	This work

N/A: Not available.

3.3 Potential for extending beyond ReLU and Sigmoid functions

While the MM-SFGM has been experimentally validated to support ReLU- and Sigmoidal-type NAFs, its physical mechanism and structural configurability suggest broader potential for implementing more complex or task-specific nonlinearities, including Leaky ReLU, ELU, GELU, and even Softmax-like transformations.

At the **device level**, the tunability of the MM-SFGM's rectifying behavior arises from the built-in potential of the programmable p–n junction, which is modulated by independently controlling the electron and hole densities stored in the split floating gates. By further adjusting the asymmetry of the Schottky barriers at the source and drain contacts—or by introducing an additional top-gate terminal to modulate the vertical field distribution—one can engineer a more gradual turn-on or soft-saturation behavior. These features are essential for emulating smooth and non-piecewise functions such as Leaky ReLU, ELU, or GELU.

At the **circuit level**, more complex activation functions that cannot be directly mapped by a single I–V curve—such as Swish or Softmax—can be approximated by cooperative networks of multiple MM-SFGM units. For instance, arrays of devices configured with exponential-like responses can be combined with analog current-mode normalization or multiplication circuits to construct Softmax-like response layers.

It is important to note that while this work focuses on demonstrating two widely used NAFs (ReLU and Sigmoid) with experimental precision, the underlying rectification mechanism is not intrinsically limited to these two functions. Instead, the MM-SFGM platform provides a hardware-reconfigurable, analog-continuous substrate upon which a broader class of nonlinear transformations can potentially be realized, subject to appropriate tuning or architectural extension.

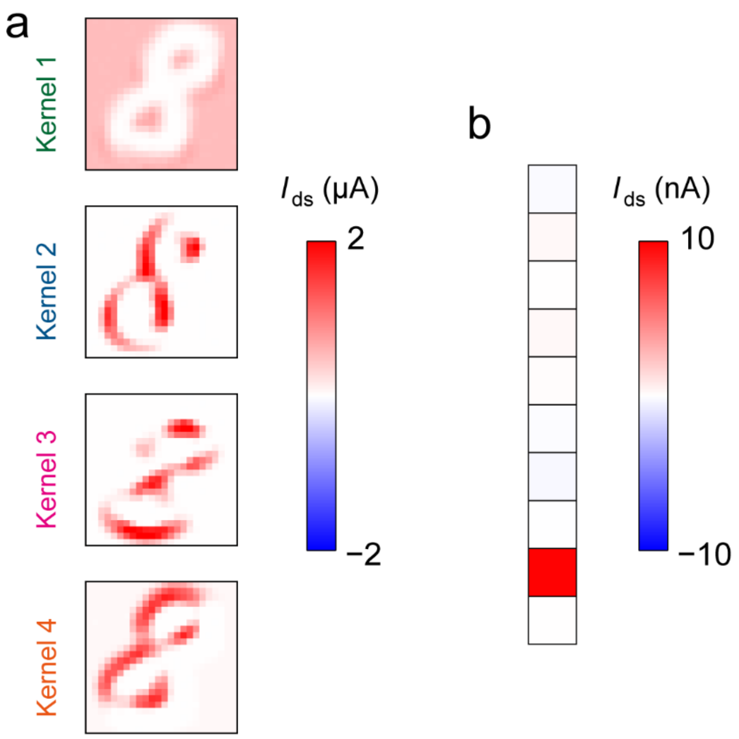

Supplementary Fig. 48| Nonlinear activation results obtained by hardware neurons. a, Output current distributions obtained by the ReLU-type hardware neurons for data from four conventional kernels. **b**, Output currents obtained by the Sigmoidal-type hardware neurons for 10 values from the output layer.

Supplementary Note 4. Uniformity of the MM-SFGM arrays

4.1 Uniformity of the morphological and spectral characterization

The MM-SFGM device arrays were fabricated using large-area 2D materials grown via CVD and EDL-confined strategies. Specifically, WSe_2 was used as the channel, and GDYO served as the TS layer—both of which are essential for the multifunctional behavior of our device.

To evaluate the material uniformity, we first conducted AFM and Raman spectroscopy at 25 randomly selected locations across a centimeter-size film for each material (Supplementary Fig. 49–52). The results confirmed highly uniform film thickness for both WSe_2 and GDYO, along with consistent Raman signatures, indicating excellent wafer-scale homogeneity.

Following this, we performed more detailed device-level characterization within the fabricated 5 × 5 × 4 array (100 devices). Each device contains patterned WSe_2 channels and GDYO switching layers derived from large-area precursor films. AFM scans of all 100 devices confirmed consistent thicknesses of both WSe_2 and GDYO layers (Supplementary Fig. 53 and 55), as well as surface roughness of the WSe_2 channels after full device processing (Supplementary Fig. 57). Raman spectra from the same 100 devices further demonstrated stable vibrational features for both materials (Supplementary Fig. 54 and 56), reaffirming the uniformity of chemical composition and crystallinity across the array.

Moreover, all these 100 GDYO flakes demonstrated volatile TS behaviors across both the positive and negative voltage regimes (within ±1 V), with the SET voltages distributing within a relatively narrow region (Supplementary Fig. 58). These morphological and spectral characterization results demonstrate the uniformity of the large-area materials used in the construction of the device array.

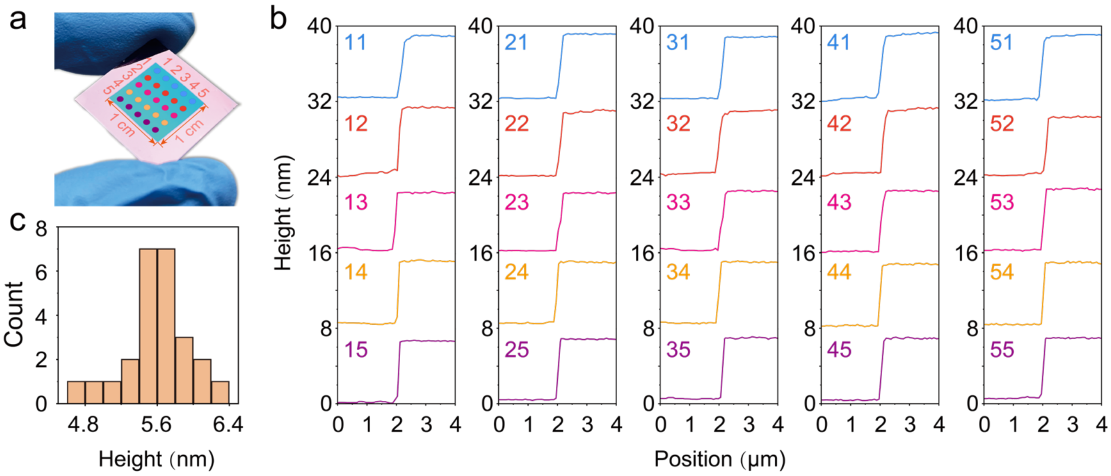

Supplementary Fig. 49| Thickness uniformity characterization of the large-area WSe_2 film. a, Photograph of a 1 cm-scale WSe_2 thin film on SiO_2/Si substrate, showing uniform color contrast. The dots indicate the positions selected for thickness and Raman spectra measurements. **b,** AFM height profiles measured at 25 randomly selected positions across the film, corresponding to the labeled points in **a**. The consistent step heights confirm the uniformity of the WSe_2 layer across the wafer. **c,** Statistical distribution of the measured film thicknesses, with an average value around 5.6 nm and a narrow spread.

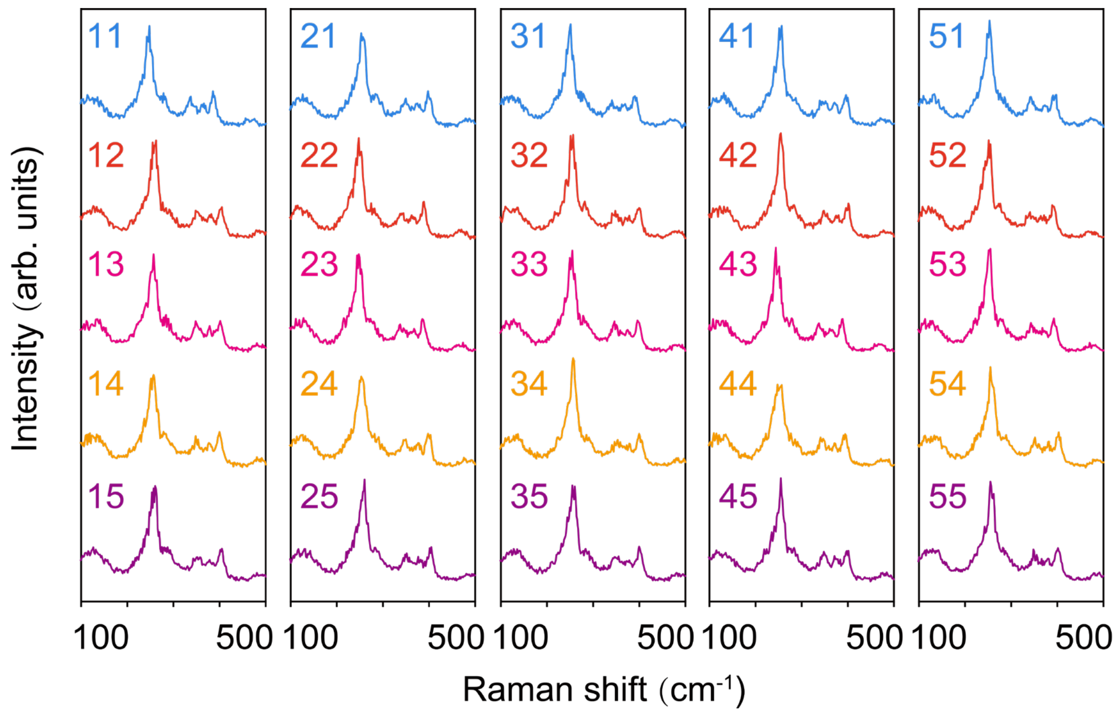

Supplementary Fig. 50| Raman spectra of the WSe_2 film. Each spectrum was measured at the corresponding WSe_2 region, as indicated in Supplementary Fig. 49a.

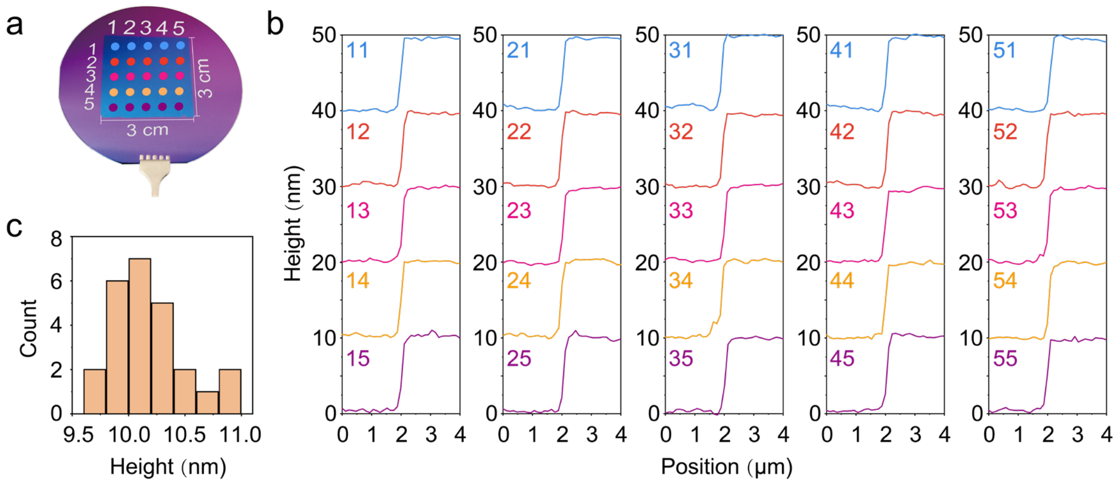

Supplementary Fig. 51| Thickness uniformity characterization of the wafer-scale GDYO film. a, Photograph of a 3 cm GDYO film on a SiO_2/Si wafer. The dots indicate the positions selected for thickness and Raman spectra measurements. **b,** AFM height profiles measured at 25 randomly selected positions across the film, corresponding to the labeled points in **a**. The consistent step heights confirm the uniformity of the GDYO layer across the wafer. **c,** Statistical distribution of the measured film thicknesses, with an average value around 10 nm.

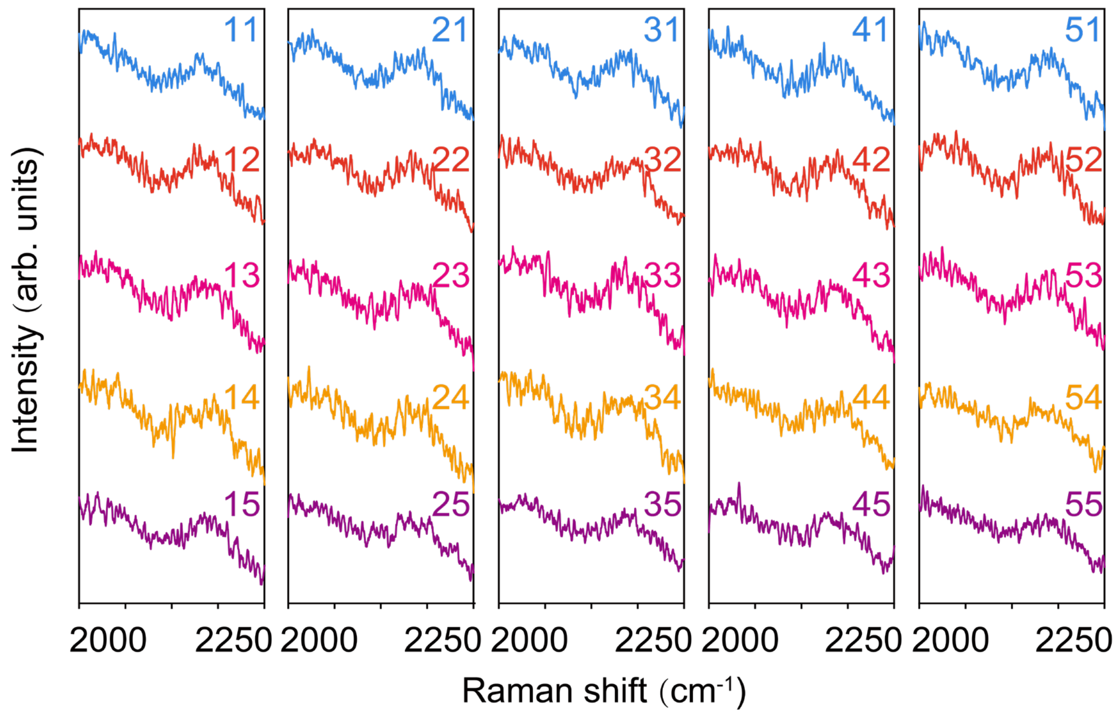

Supplementary Fig. 52| Raman spectra of the wafer-scale GDYO film. Each spectrum was measured at the corresponding GDYO region, as marked in Supplementary Fig. 51a.

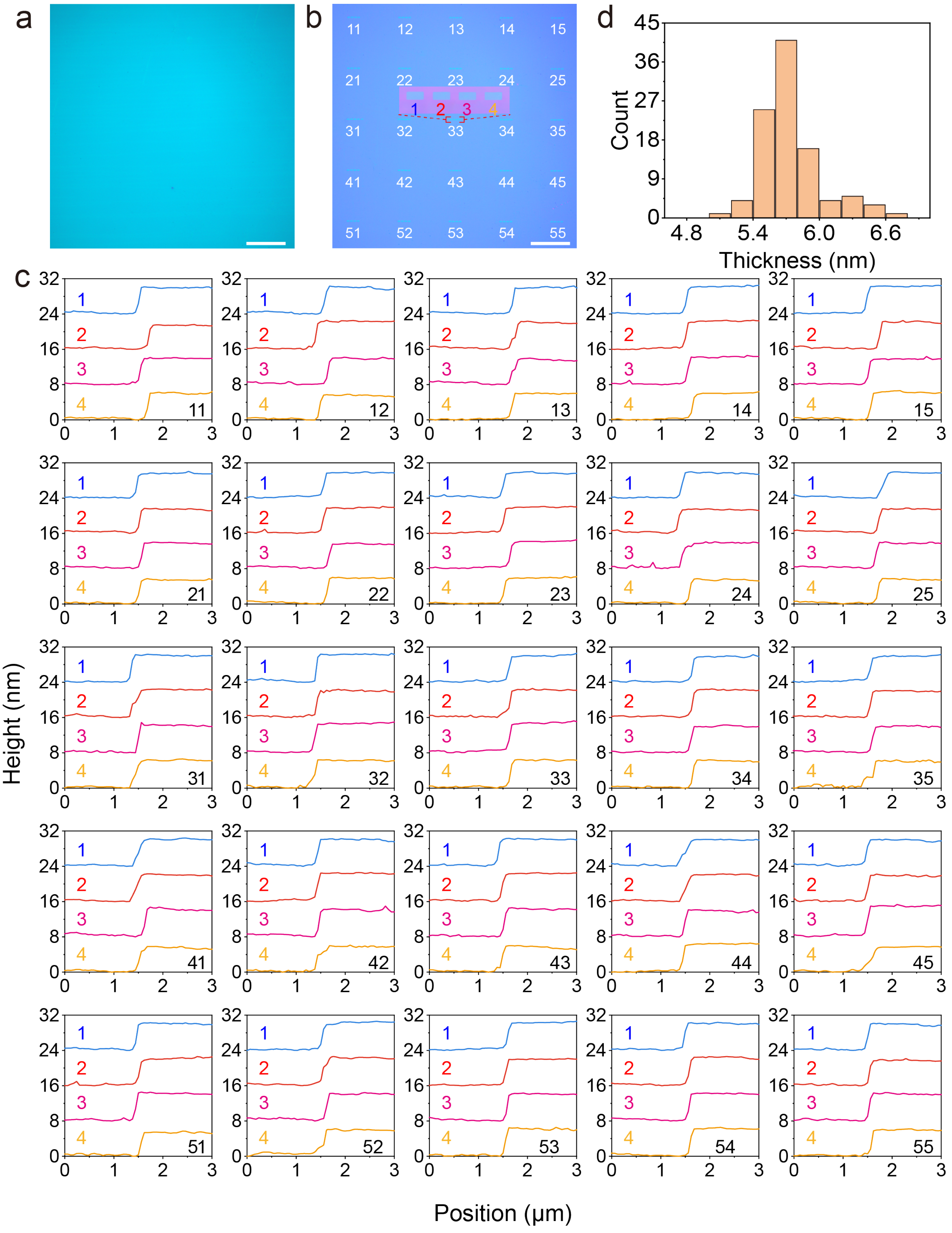

Supplementary Fig. 53| Morphological characterization of the 5 × 5 × 4 WSe_2 array. **a**,**b**, Optical image of the 5 × 5 × 4 WSe_2 array before (**a**) and after fabrication (**b**). Inset shows an enlargement of the pixel 33, which consists of four subpixels. Scale bars, 150 μm. **c**, Height profiles of the 5 × 5 × 4 WSe_2 array. The indices (11 to 55) correspond to the pixels marked in **c**, and the numbers 1 to 4 refer to the four subpixels in each pixel. **d,** Thickness distribution of 5 × 5 × 4 WSe_2 arrays in **c**.

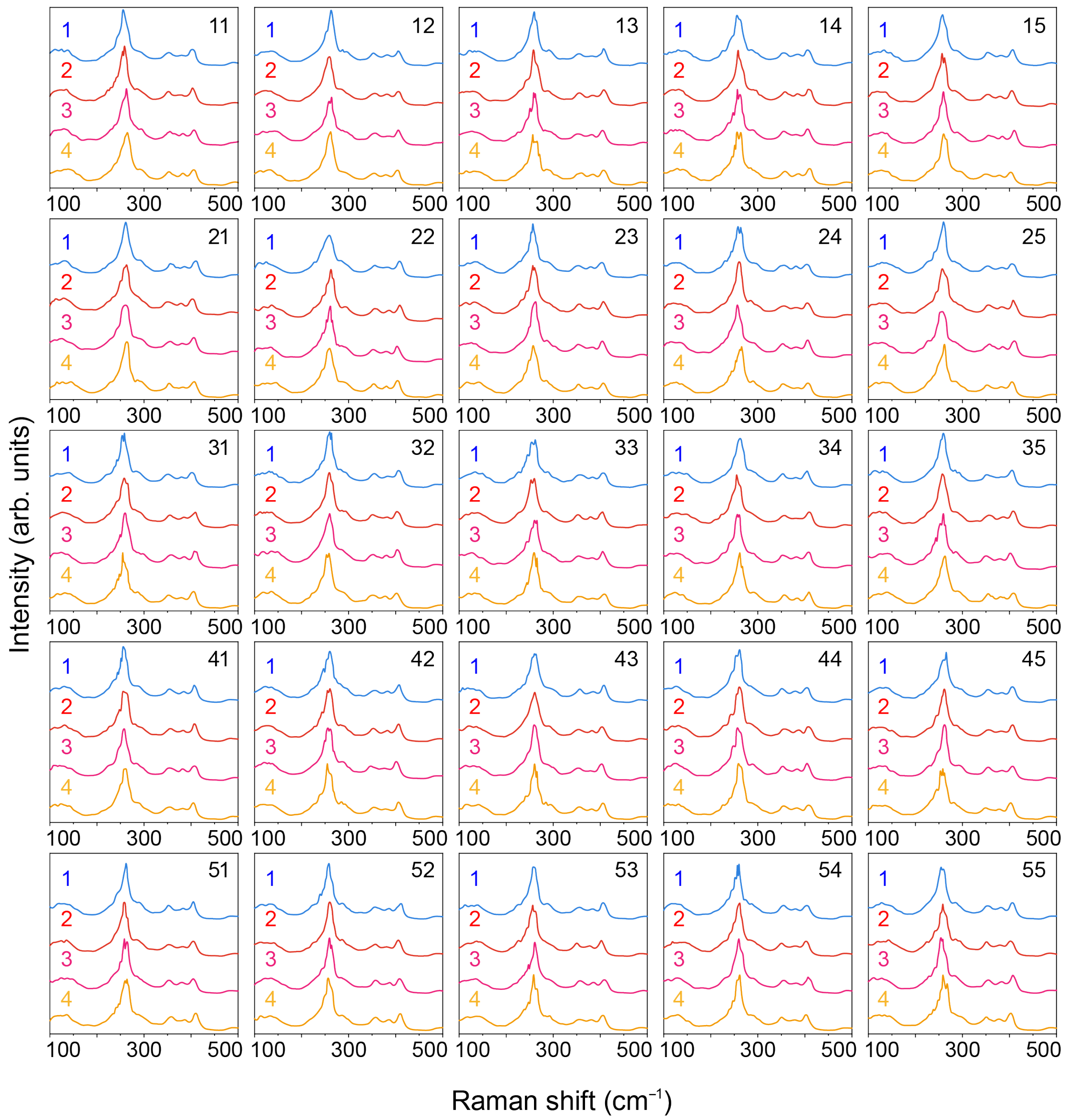

Supplementary Fig. 54| Raman spectra of the 5 × 5 × 4 WSe_2 array. Each spectrum was measured at the corresponding WSe_2 channel, as indicated in Supplementary Fig. 53b.

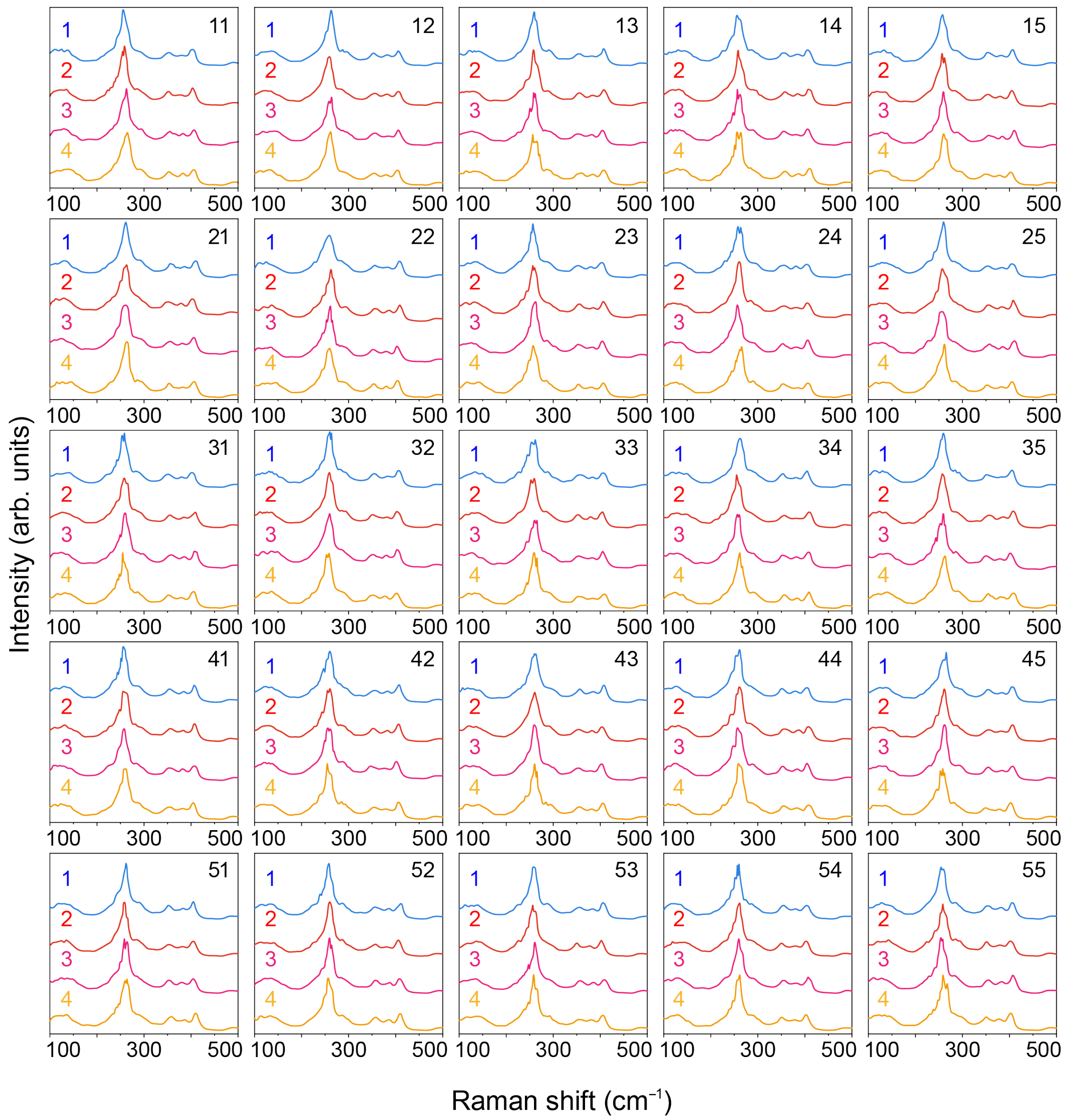

Supplementary Fig. 55| Morphological characterization of the 5 × 5 × 4 GDYO array. a,**b**, Optical image of the 5 × 5 × 4 GDYO array before (**a**) and after fabrication (**b**). Scale bars, 150 μm. **c**, Height profiles of the 5 × 5 × 4 GDYO array. The indices (11 to 55) correspond to the pixels marked in **c**, and the numbers 1 to 4 refer to the four subpixels in each pixel. **d,** Thickness distribution of 5 × 5 × 4 GDYO arrays in **c**.

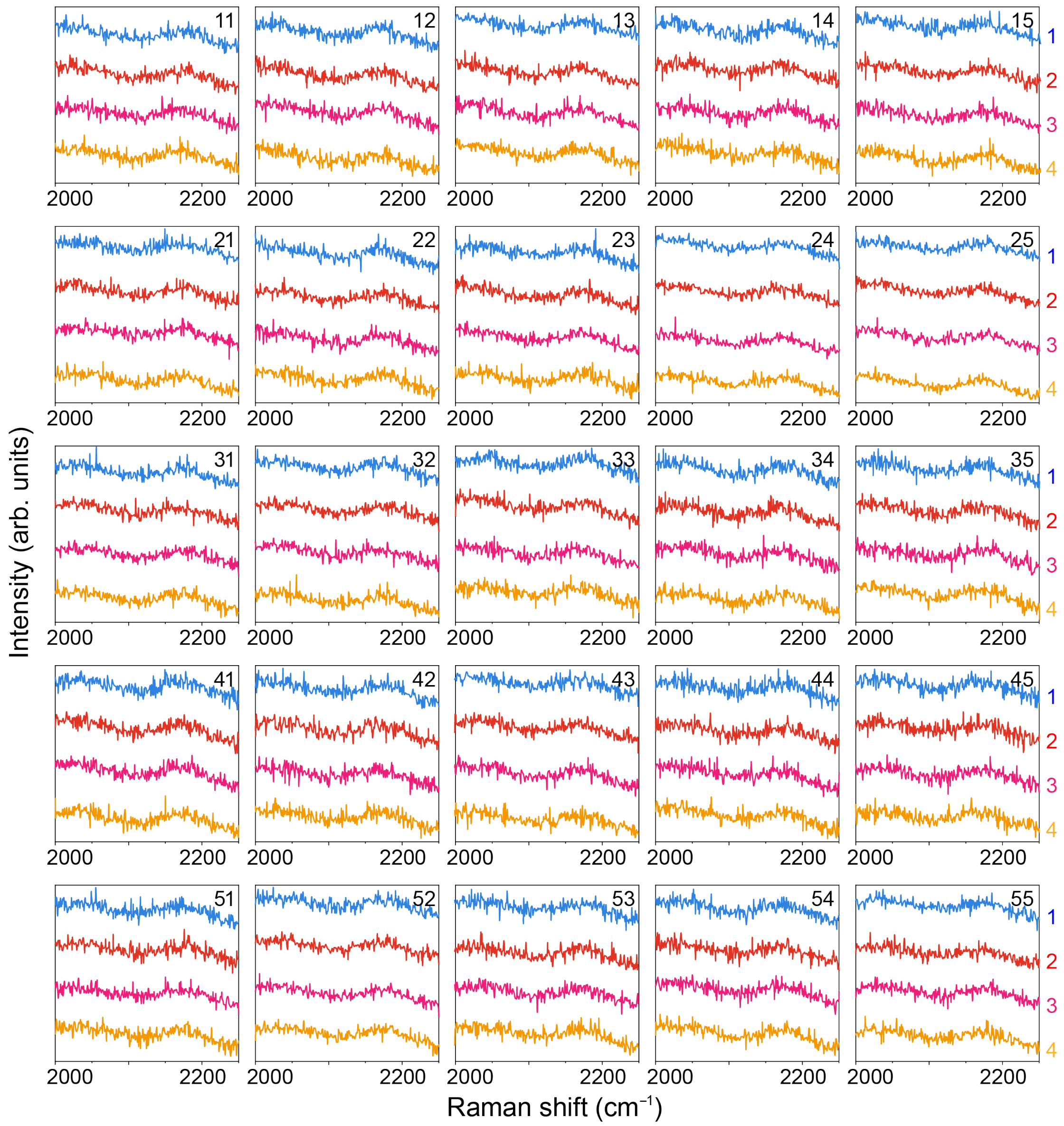

Supplementary Fig. 56| Raman spectra of the 5 × 5 × 4 GDYO array. Each spectrum was measured at the corresponding GDYO region, as indicated in Supplementary Fig. 55b.

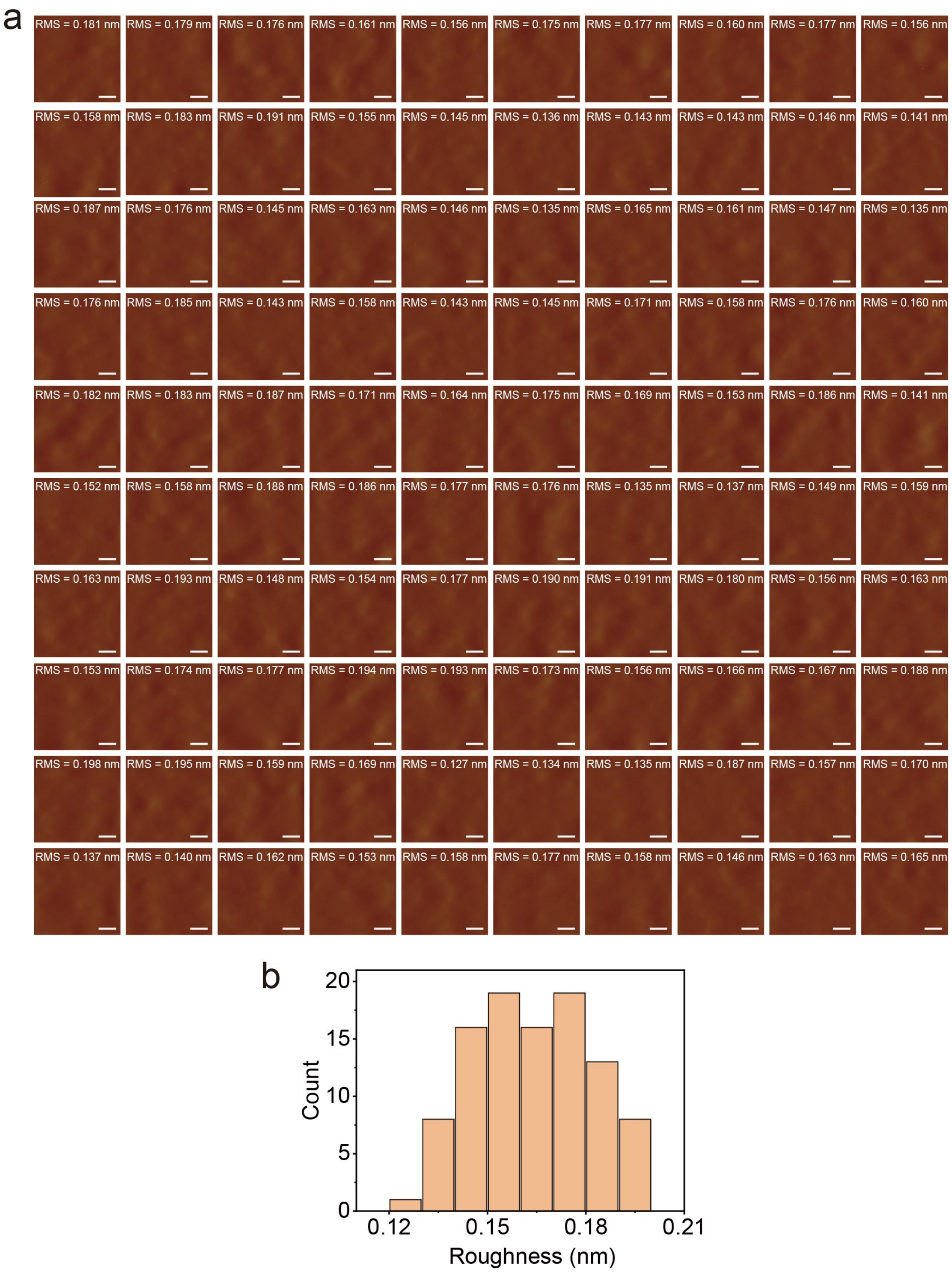

Supplementary Fig. 57| Surface roughness characterization of 100 WSe_2 channels after device fabrication. a, AFM images of the channel regions from 100 fabricated devices. Scale bar, 500 nm. **b,** Statistical distribution of the root-mean-square (RMS) roughness across the 100 devices, showing an average surface roughness around 0.16 nm and a narrow spread, indicating high uniformity of the WSe_2 channel surfaces.

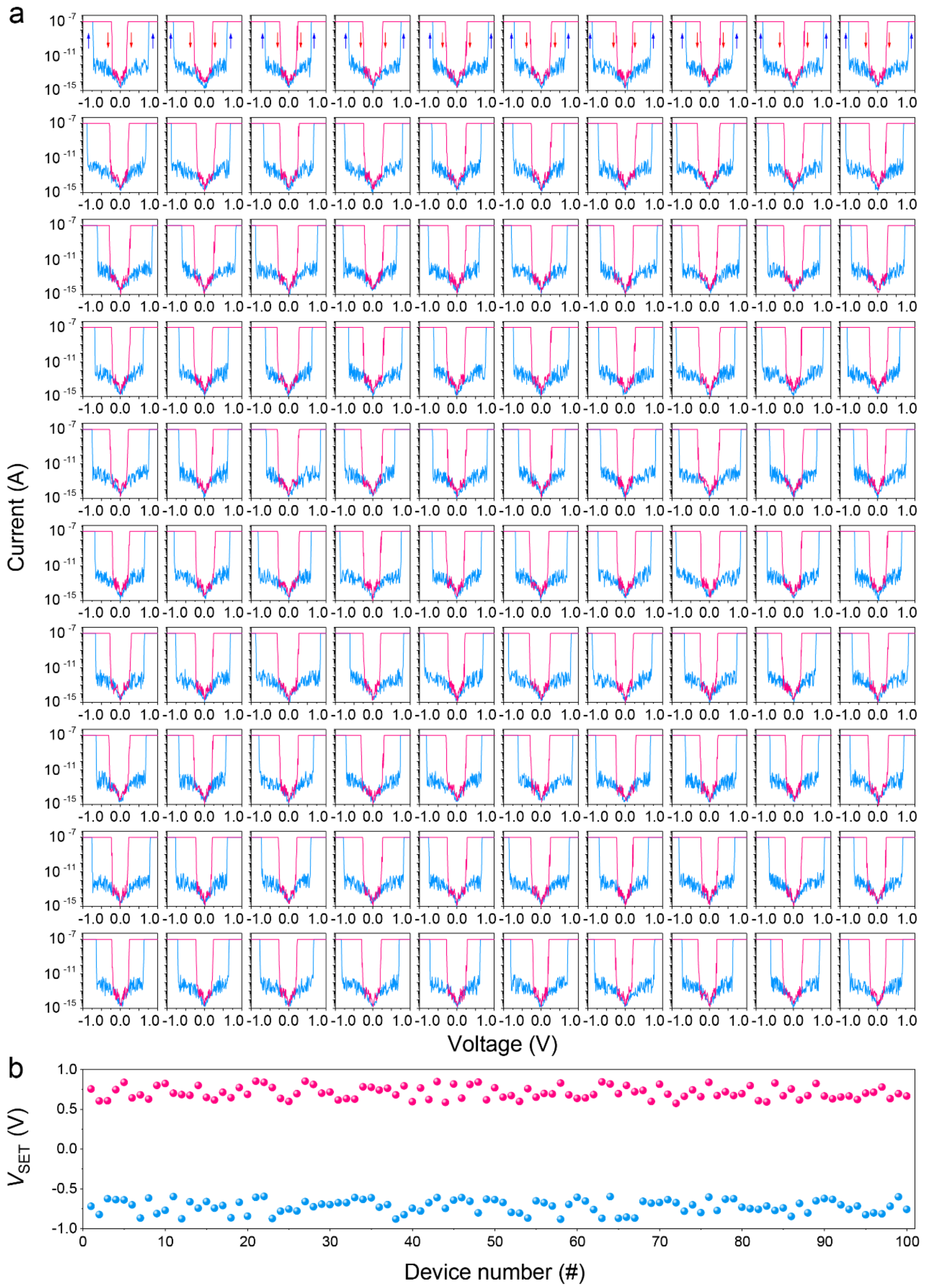

Supplementary Fig. 58| TS behaviors of the 5 × 5 × 4 GDYO array. a, *I–V* curves of the 100 GDYO flakes, measured by sweeping the voltage 0 V → 1 V → 0 V → –1 V → 0 V. **b**, Distribution of SET voltages for the 100 GDYO TS layers in positive (pink) and negative (blue) regimes.

4.2 Uniformity of the weight updates for ISC and IMC

The MM-SFGM utilizes photoresponsivity (*R*) and conductance (*G*) as the weights in neural networks, enabling the execution of MAC operations for optical and electrical input signals in ISC and IMC, respectively. Ideally, all devices that constitute the neural network should have identical performance to ensure the highest computational accuracy. However, due to factors such as material uniformity and fabrication processes, device-to-device variation (DDV) is inevitable, leading to discrepancies in the computation results and potentially affecting the accuracy. Supplementary Fig. 59 shows the distributions of the conductance levels for 100 devices in p-p, n-n, p-n, and n-p states, demonstrating a high uniformity.

To further assess the impact of DDV on ISC and IMC, the weight update performance of all 100 devices in the 5 × 5 × 4 array were investigated. Supplementary Fig. 60 and 61 show the LTP and LTD processes for all 100 devices using *R* as the weight. The DDVs for LTP and LTD are calculated as 6.5% and 7.2%, respectively (Supplementary Note 4). The small DDV enables the devices to be set to similar weight states, producing closely matched computational outputs. Supplementary Fig. 62 illustrates the output photocurrent distributions from the 5 × 5 × 4 array under a 0.5 W cm^{-2} light illumination, where the devices are programmed to five different photoresponsivity states (±80 mA W^{-1}, ±40 mA W^{-1}, and 0 mA W^{-1}). The DDVs for these outputs are all below 7%.

Similarly, the conductance update processes of these 100 devices were also measured. As shown in Supplementary Fig. 63 and 64, their LTP and LTD characteristics were found to be very similar, with DDVs of 6.9% and 6.4%, respectively. Supplementary Fig. 65 shows the output results of these devices for a 0.5 V electrical input, with the conductance states programmed to 0, 2, 4, 6, and 8 μS, respectively. The DDVs for all the output currents are below 6.7%. These results demonstrate that the 100 devices used for ISC and IMC operations exhibit a small DDV (~7%), confirming their suitability for consistent and accurate computational tasks.

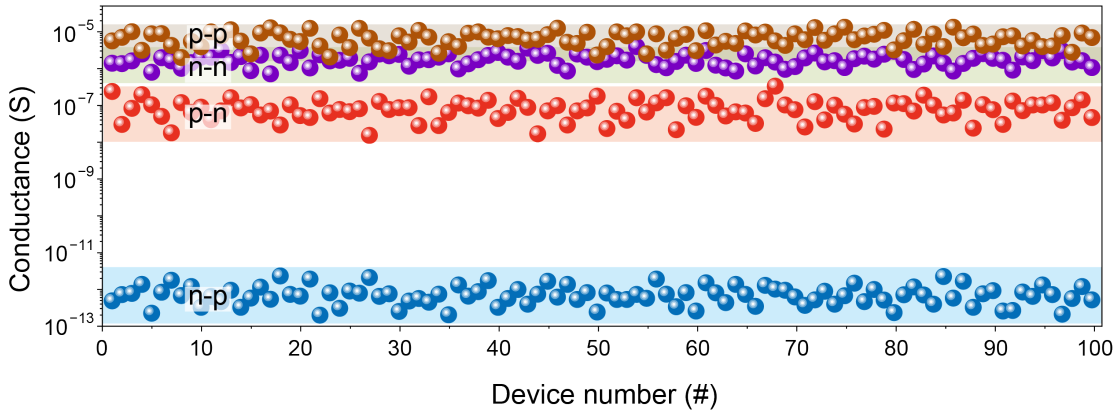

Supplementary Fig. 59| Conductance distribution of 100 MM-SFGMs. The as-fabricated 100 devices were programmed to n-p, p-n, n-n, and p-p states and their conductance values were measured by applying a 0.5 V read voltage. The narrow distribution of these conductance states indicates that all these 100 devices can switch between the four homojunction states to perform various tasks.

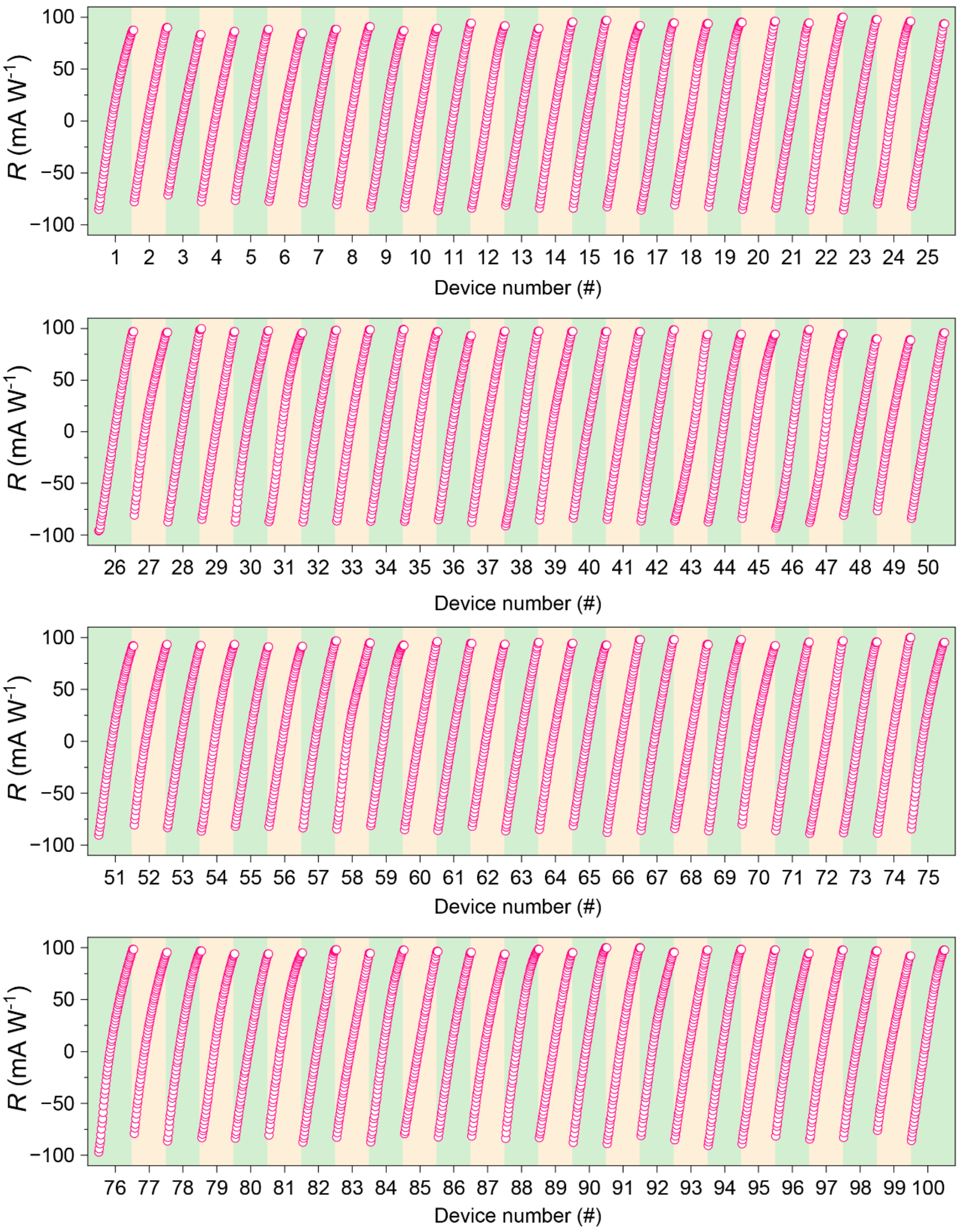

Supplementary Fig. 60| LTP photoresponsivity update behaviors of the 100 devices for ISC.

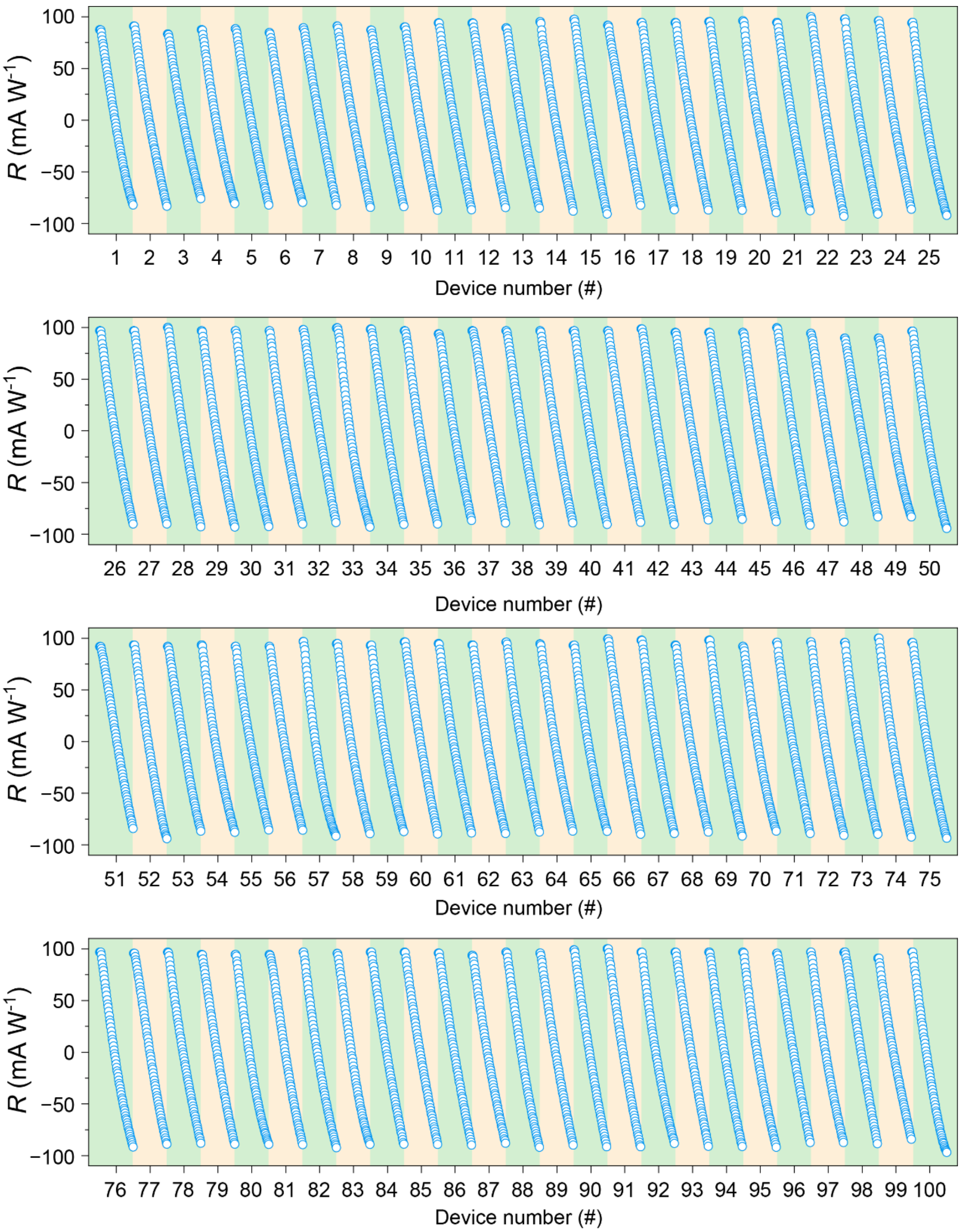

Supplementary Fig. 61| LTD photoresponsivity update behaviors of the 100 devices for ISC.

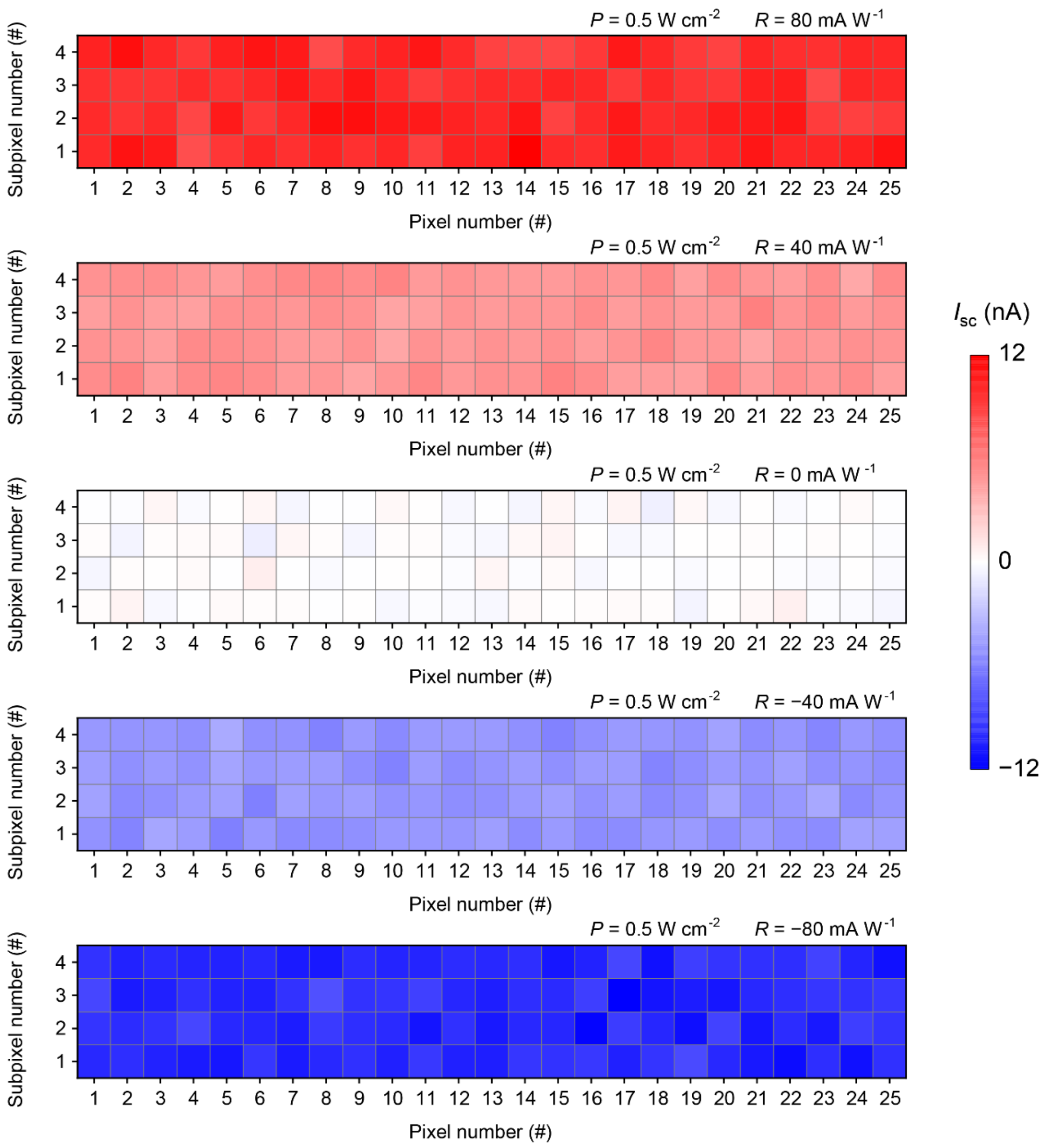

Supplementary Fig. 62| Output photocurrent distributions of the 5 × 5 × 4 device array. All 100 devices were programmed to the same photoresponsivity states (±80, ±40, and 0 mA W^{-1}) and illuminated with a light of 0.5 W cm^{-2}.

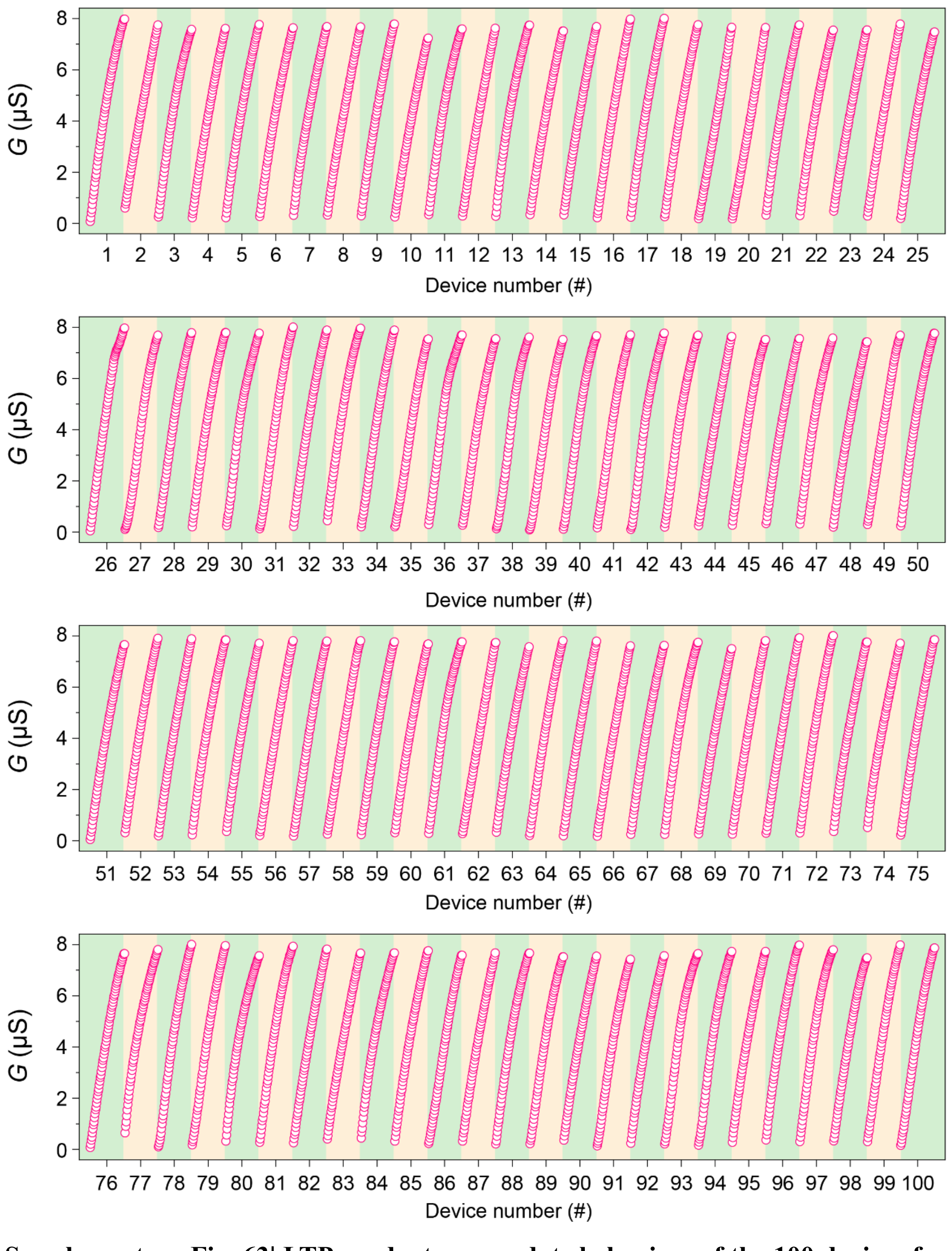

Supplementary Fig. 63| LTP conductance update behaviors of the 100 devices for IMC.

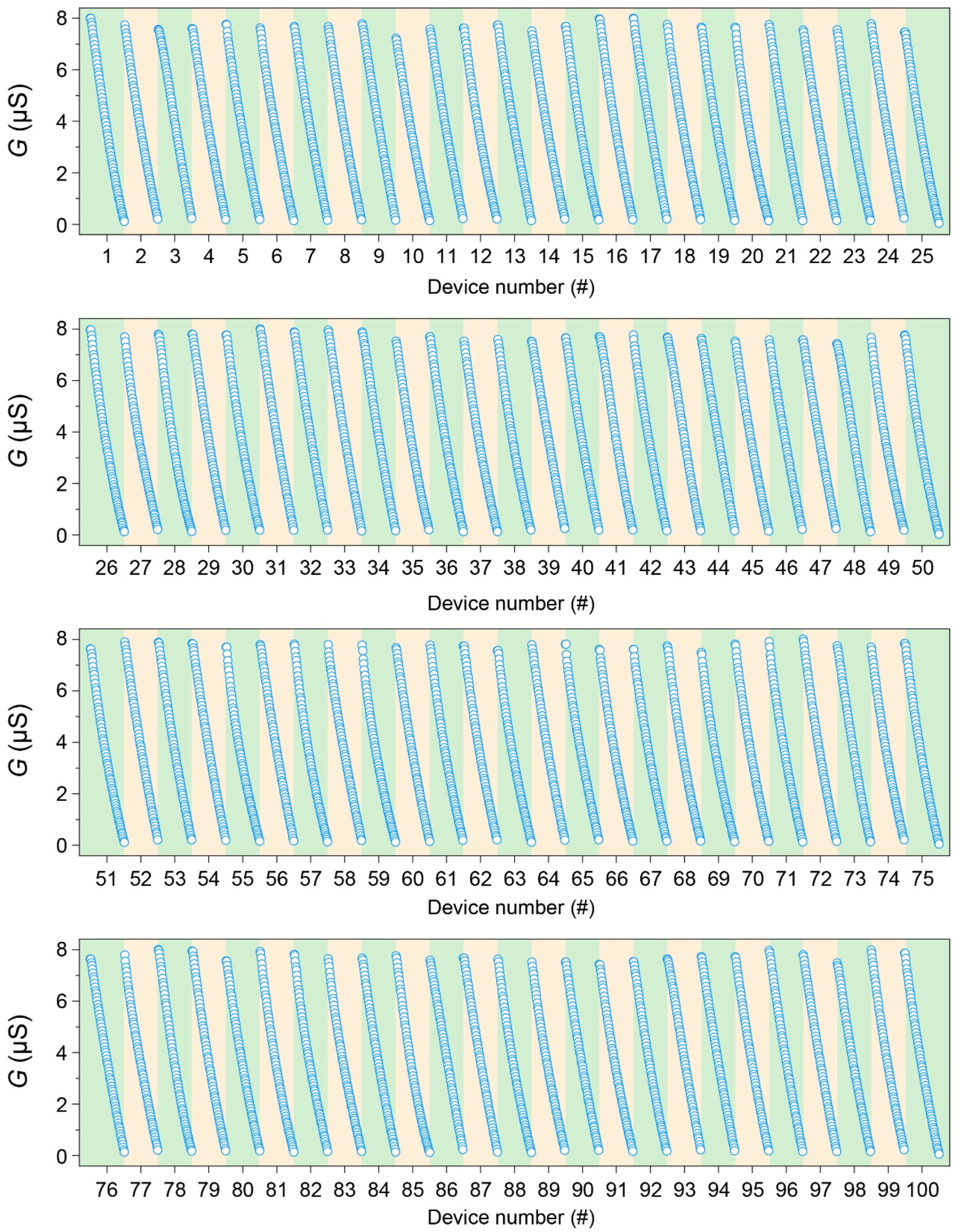

Supplementary Fig. 64| LTD conductance update behaviors of the 100 devices for IMC.

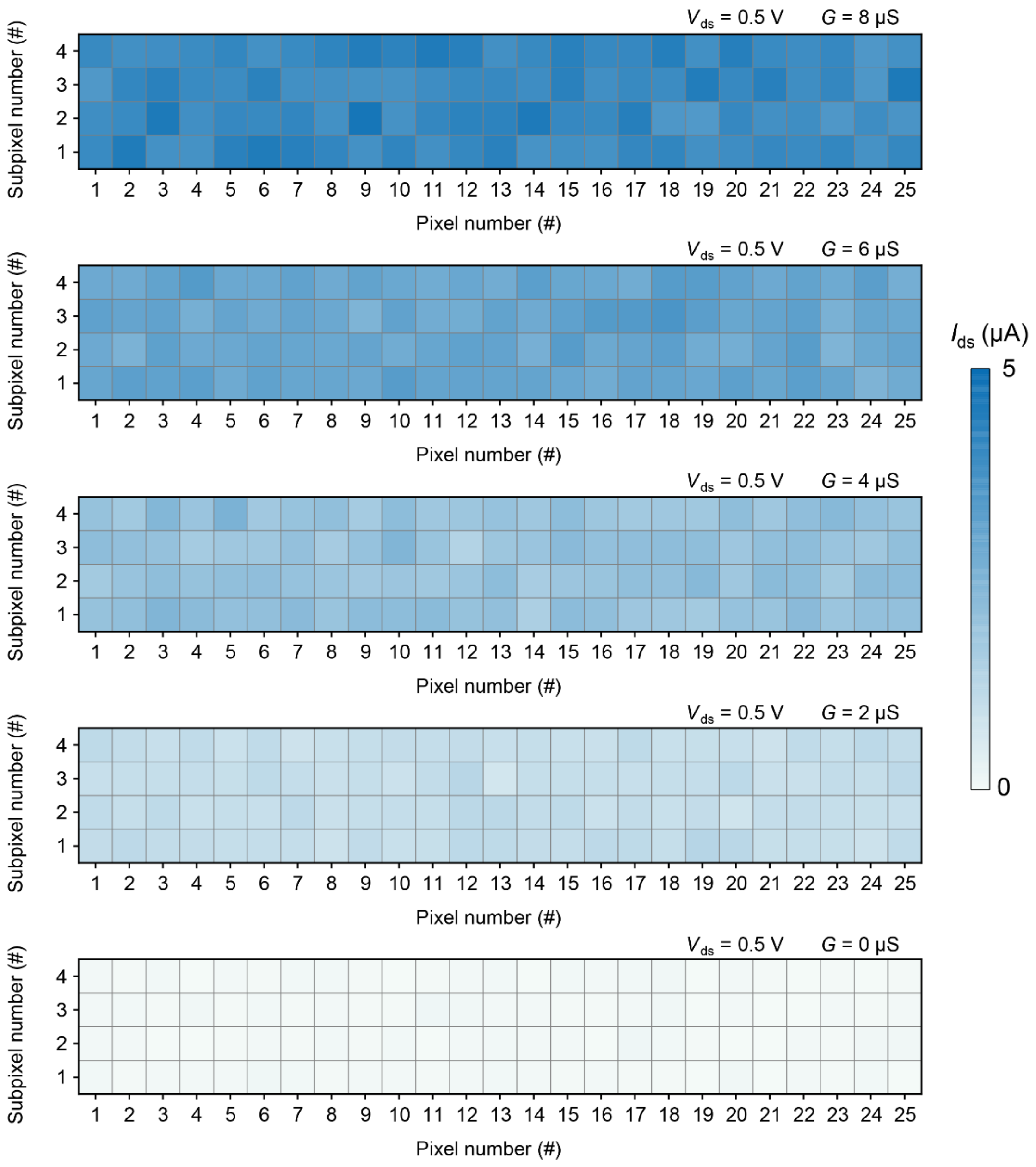

Supplementary Fig. 65| Output current distributions of the 5 × 5 × 4 device array. All 100 devices were programmed to the same conductance states (0, 2, 4, 6, and 8 μS) and driven by a 0.5 V input voltage.

4.3 Uniformity of the rectification characteristics for NAFs

In this work, we utilize the tunable rectification characteristics of the MM-SFGM to achieve ReLU and Sigmoidal NAFs. A 5 × 5 device array was specifically fabricated to perform nonlinear activation tasks in neural networks. To assess the uniformity of the devices for NAFs, the rectification behaviors of all 25 devices were investigated under low V_T conditions (for ReLU) and high V_T conditions (for Sigmoidal NAF). Supplementary Fig. 66 presents the rectification characteristics of the devices in a lightly doped state (p^--n^-), along with the distribution of their corresponding V_T. We also statistically analyzed the nonlinear activation results for these devices under different input signals, finding that the DDV for all outputs was less than 10%. Supplementary Fig. 67 shows the rectification behaviors and V_T distribution of the devices in a highly doped state (p^+-n^+). The DDV for the devices performing Sigmoidal NAF was similarly found to be under 10%. These results demonstrate that the device array used for nonlinear activation tasks can effectively switch between different rectifying states, with the DDV remaining below 10% across all conditions.

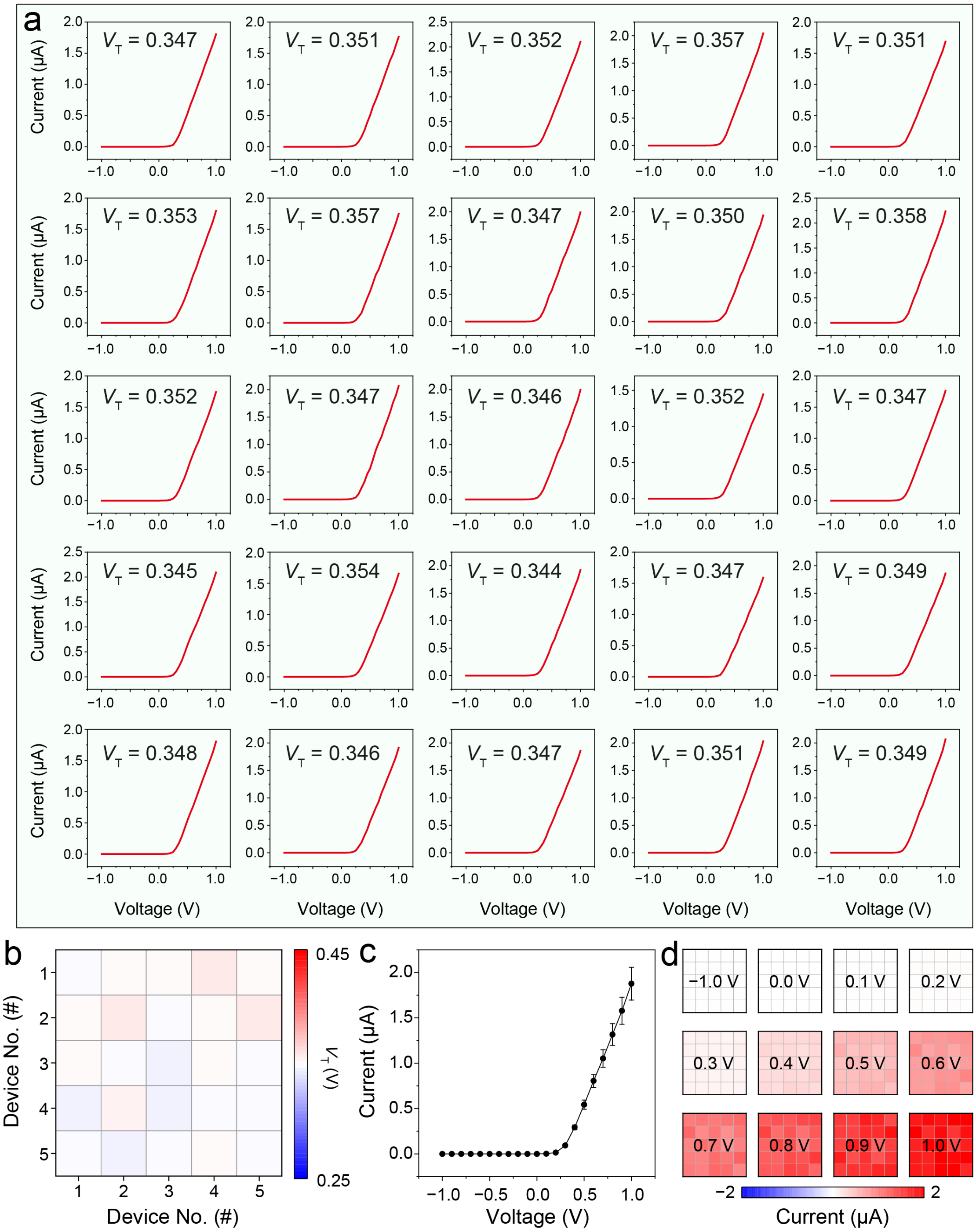

Supplementary Fig. 66| Performance of 25 neuron-mode MM-SFGM for ReLU operations. **a**, I–V curves of 25 neuron-mode devices with low V_T. **b**, distribution of V_T for the 5 × 5 device array. **c**, Statistics of the 25 devices' output currents for ReLU. **d**, Output current distributions of the 5 × 5 device array for different input voltages.

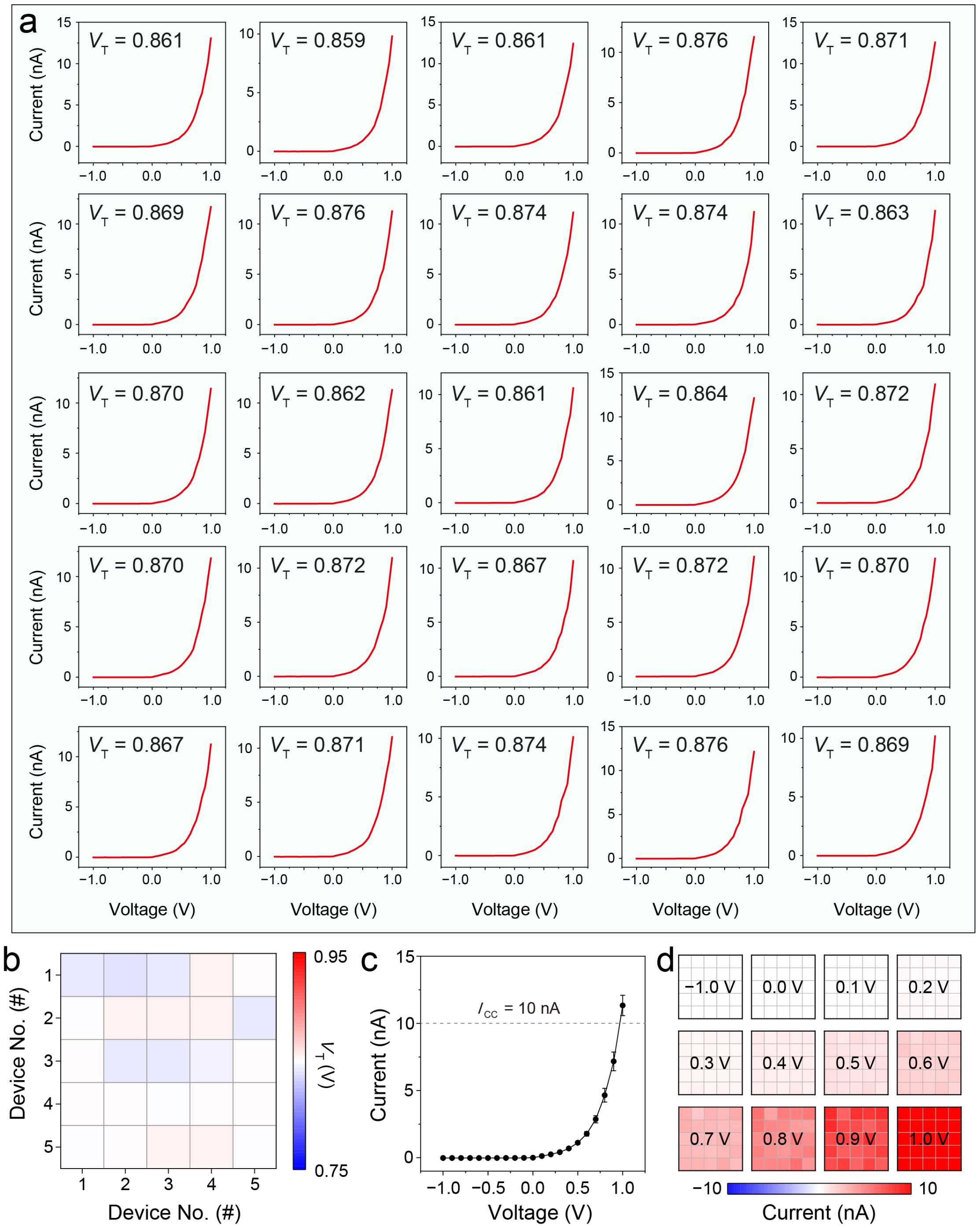

Supplementary Fig. 67| Performance of 25 neuron-mode MM-SFGM for Sigmoidal operations. a, *I*–*V* curves of 25 neuron-mode devices with high V_T. **b**, distribution of V_T for the 5 × 5 device array. **c**, Statistics of the 25 devices' output currents for Sigmoidal NAF. **d**, Output current distributions of the 5 × 5 device array for different input voltages.

4.4 Influence of the DDV on neural network performance

DDV is a critical factor affecting the reliability and accuracy of neuromorphic hardware systems. In our MM-SFGM platform, the experimentally measured DDV for both conductance (used in IMC) and photoresponsivity (used in ISC) is approximately 7%, while the DDV for NAFs is around 10%. Although this level of variation is higher than that of commercial CMOS neuromorphic platforms (e.g., <1% DDV in Intel Loihi), it reflects the current limitations of laboratory-scale fabrication using research-grade CVD and ALD tools. Importantly, we show that the observed DDV remains well within acceptable margins for analog neural network computation.

To quantitatively evaluate the impact of DDV on recognition accuracy, we performed simulations using the same CNN structure implemented in hardware (Fig. 4f). In this architecture, four 5 × 5 convolutional kernels perform in-sensor convolution on 28 × 28 grayscale MNIST images, followed by ReLU NAFs, and then a 2304 × 10 fully connected IMC layer. Sigmoidal NAFs complete the inference pipeline by producing class probabilities. We independently varied the DDV in ISC/IMC weights ($DDV_{weights}$) and in NAF characteristics (DDV_{NAFs}) from 0% to 30%. As shown in Supplementary Fig. 68, the CNN maintains >90% accuracy when $DDV_{weights}$ < 10% and DDV_{NAFs} < 25%. Under our measured values ($DDV_{weights}$ ≈ 7%, DDV_{NAFs} ≈ 10%), the network achieves 94% recognition accuracy. Notably, the experimentally obtained accuracy of 95.2% (using hardware NAFs and software-based ISC/IMC) is closely aligned with the simulated result under 0% weight variation and 10% NAF variation (95.4%), thereby validating the accuracy and reliability of the simulation framework.

We further validated the hardware's functional accuracy by implementing convolutional kernels using 36 MM-SFGM devices in a 5 × 5 × 4 array. As shown in Supplementary Fig. 69, these kernels—programmed with varying photoresponsivity and conductance profiles—performed accurate edge detection and sharpening operations on grayscale images, with output maps closely matching simulation results.

To explore performance under larger networks and more complex tasks, we conducted additional simulations using deeper CNN models for the MNIST and

CIFAR-10 datasets (Supplementary Fig. 70). With ideal device parameters, the CNN models achieve ~98% accuracy on MNIST and ~90% on CIFAR-10. Simulating the same models using our experimentally extracted DDVs results in a minimal accuracy drop, yielding ~97% on MNIST and ~88% on CIFAR-10, demonstrating robustness even in more complex tasks.

Finally, we note that DDV-induced accuracy degradation can be further mitigated using device-aware training strategies. By incorporating measured conductance and responsivity values into the training process, the network learns to compensate for non-idealities, a technique widely adopted in memristor-based systems to enhance inference robustness.

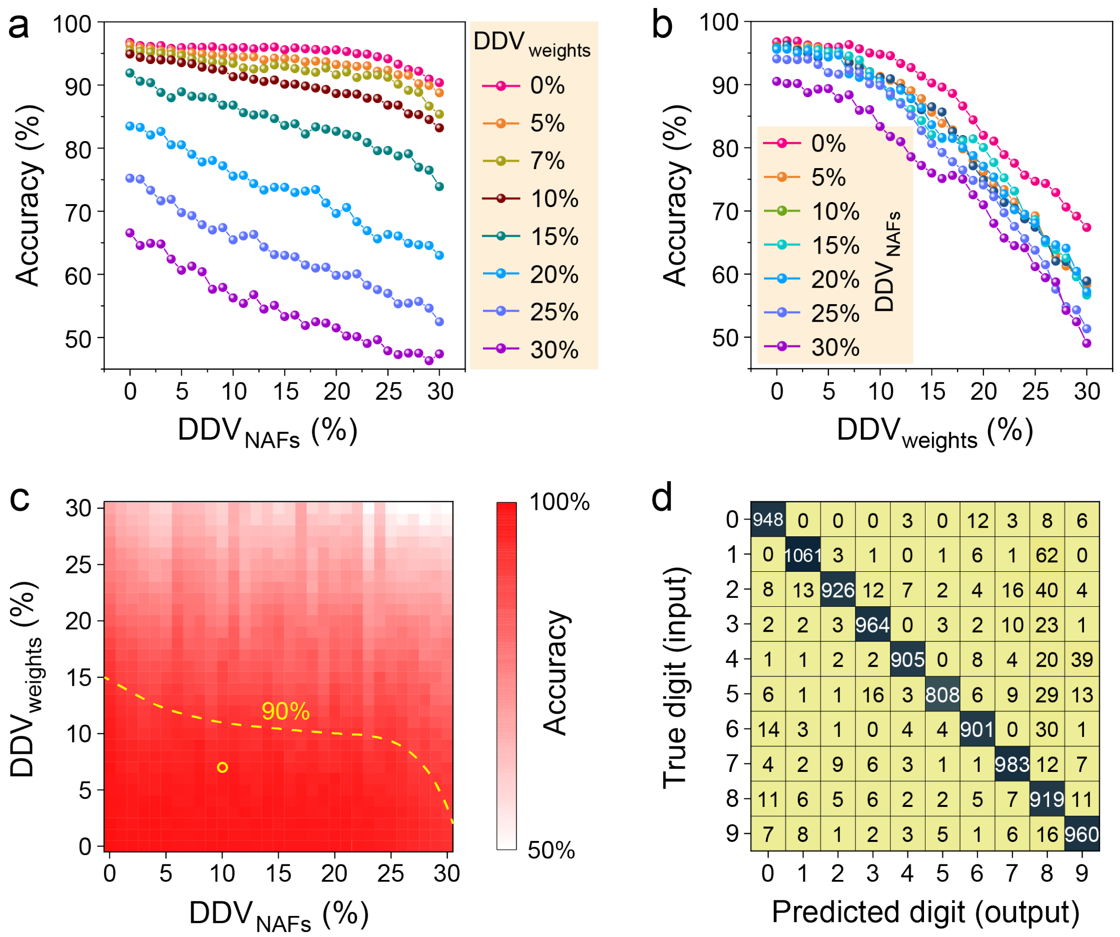

Supplementary Fig. 68| Simulation results for a CNN with different DDV levels. **a**,**b**, Evolutions of recognition accuracy as a function of $DDV_{weights}$ (**a**) and DDV_{NAFs} (**b**). **c**, Distribution of recognition accuracy for the CNN with different $DDV_{weights}$ and DDV_{NAFs} parameters. The yellow dashed line represents the boundary of 90% accuracy, and the yellow circle refers to the parameters of $DDV_{weights}$ = 7% and DDV_{NAFs} = 10%.

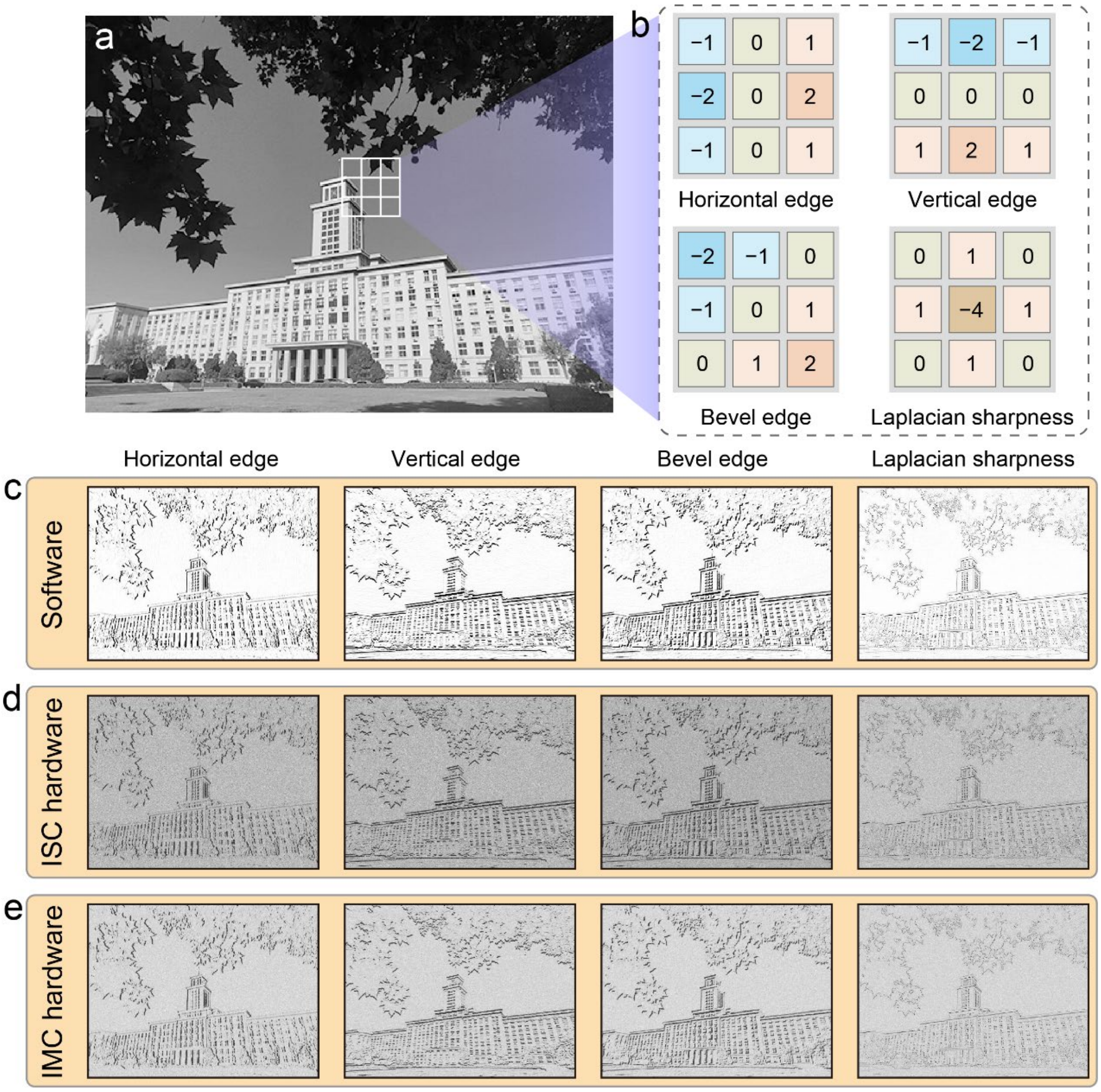

Supplementary Fig. 69| Convolutional operations executed via ISC and IMC hardware. a, Original gray-scale image for convolutional operations. **b**, Four convolutional kernels (3 × 3) for edge detection and sharpness. **c**, Software simulation results for the convolution of the original image. **d**,**e**, Experimental results using the sensor-mode (ISC hardware) and synapse-mode (IMC hardware) device arrays to execute convolutional operations.

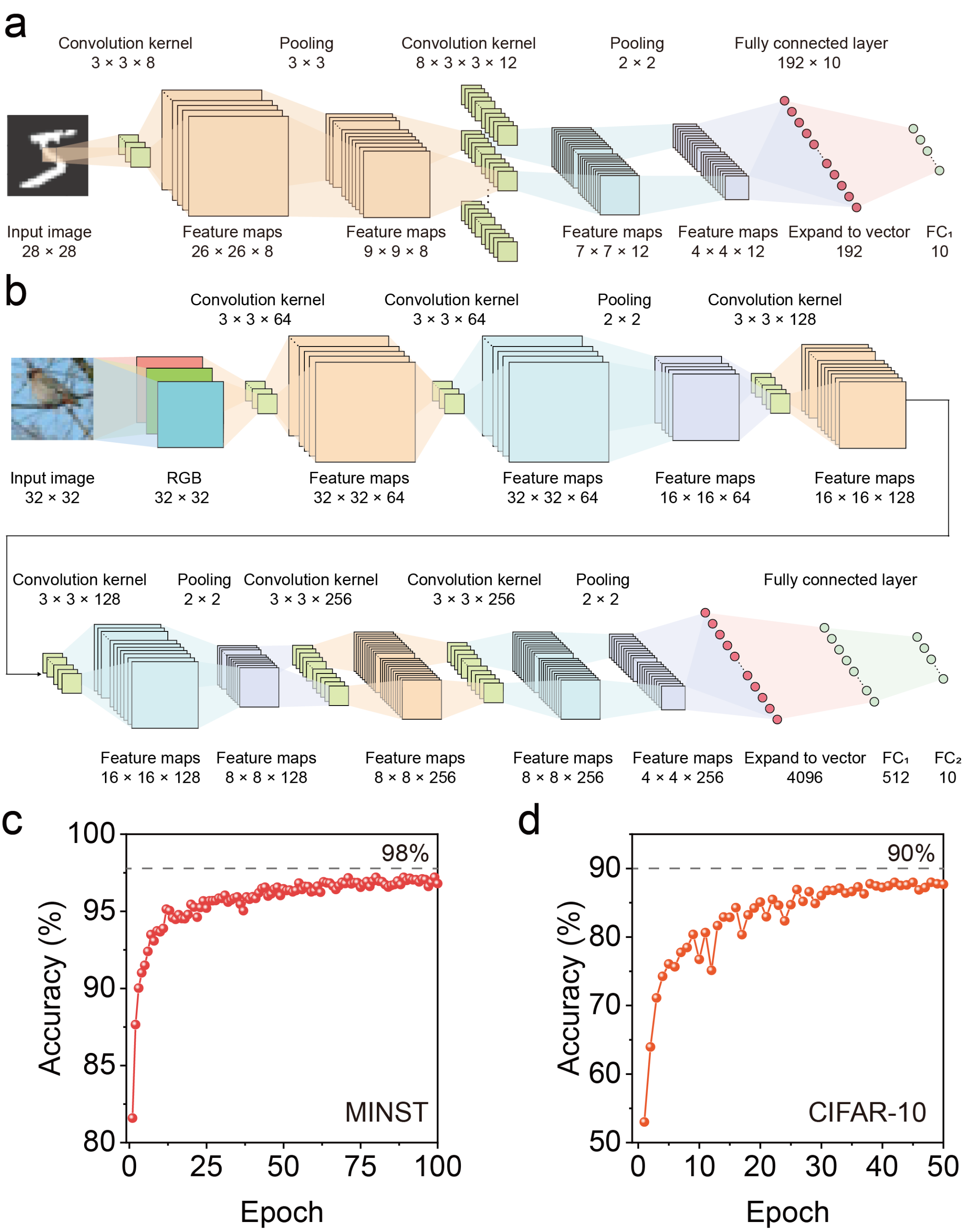

Supplementary Fig. 70| Simulation of deep CNNs for MNIST and CIFAR datasets. **a**,**b**, Network architectures used for handwritten digit recognition on the MNIST dataset (**a**) and natural image classification on the CIFAR-10 dataset (**b**). **c**,**d**, Accuracy evolution of the CNNs on MNIST (**c**) and CIFAR-10 (**d**) datasets using corresponding models. The dashed lines represent the benchmarks achieved by the networks with ideal parameters.

4.5 Challenges and solutions for scaling up the array in NMVS applications

To support the deployment of NMVS in practical scenarios, scaling up MM-SFGM device arrays beyond the current 100-device demonstration is essential. This section outlines the key challenges and corresponding strategies for large-scale implementation.

i) Device uniformity and yield

The primary challenge in scaling up lies in ensuring the uniformity of large-area 2D materials and the reliability of processing techniques for large-scale 2D devices. Current methods for producing wafer-scale 2D materials are well-established. As shown in Supplementary Fig. 49–52, large-area WSe_2 and GDYO films exhibit excellent uniformity. Moreover, significant progress has been made in the fabrication of large-scale 2D devices, enabling the processing of arrays containing millions of devices[17]. By further optimizing material synthesis and device processing techniques, we can maintain high uniformity and yield across large arrays. Additionally, modular, redundant, and fault-tolerant designs can enhance the system's tolerance to device performance variations and yield fluctuations, ensuring consistent performance across the entire array.

ii) Modular Design

Instead of relying on a monolithic array, the system can be partitioned into independently operating sub-arrays, each equipped with localized control and interconnects. These modular units can be flexibly assigned as ISC, IMC, or NAF blocks according to task-specific requirements. During training or inference, selected modules can be dynamically activated, deactivated, or reconfigured to balance computational load or bypass faulty regions. For instance, if a particular IMC module exhibits performance degradation or excessive noise, an idle module can be reassigned to perform the same function without compromising system-level operation.

iii) Fault-tolerant design

System robustness can be improved through a combination of variation-aware training and hardware redundancy. Initially, the electrical characteristics of each

device are characterized and incorporated into the training process to enable optimized weight allocation that accounts for individual device behavior. In addition, critical weights may be redundantly mapped across multiple IMC sub-arrays. During inference, outputs from mirrored modules can be cross-validated using voting mechanisms to ensure reliable outcomes even in the presence of device failures. This hybrid approach enhances fault tolerance and supports reliable operation in large-scale neuromorphic arrays.

iv) Sneak paths

Sneak paths, a common issue in IMC arrays, can lead to unwanted signal interference and cross-talk. This can be mitigated by improving isolation between devices to reduce unwanted coupling. Optimizing circuit design and signal control can also ensure that input signals are applied exclusively to the intended computational units, preventing interference with neighboring devices. Alternatively, incorporating transistors or rectifiers in series can block irrelevant sneak paths, further reducing the risk of signal leakage.

v) High line resistance

To minimize line resistance, high-conductivity metals should be used for electrode materials, with careful consideration of the optimal balance between electrode size and density. Additionally, employing modular designs and optimizing circuit layouts can shorten interconnect lengths, further reducing line resistance and enhancing overall system efficiency.

vi) Integration and interconnects

Advanced interconnection techniques, such as through-chip vias and 3D stacking[18], can reduce the need for long-distance interconnects, thus minimizing signal delay and power consumption. Another approach is to adopt modular designs, where smaller subarrays function independently and can be interconnected with minimal interference. This method not only simplifies integration but also provides flexibility for scaling up, enabling more efficient system expansion.

vii) 3D integration

To improve vertical integration density and minimize lateral interconnect delays, functional layers—such as ISC, IMC, and NAF—can be vertically stacked using 3D integration techniques. For instance, the ISC layer may occupy the uppermost tier, with its output signals routed via vertical interconnects (e.g., through-silicon vias) to underlying IMC and NAF layers. This architecture reduces horizontal wiring complexity and improves spatial efficiency. Recent advances in monolithic 3D integration of 2D materials have demonstrated the feasibility of low-temperature, tier-by-tier lamination with up to 10 vertically stacked device layers[18]. Such integration strategies are well-suited for building high-density, multifunctional neuromorphic systems.

Supplementary Note 5. System-level benchmarking and energy-performance analysis

5.1 System architecture based on reconfigurable MM-SFGM arrays

To validate the feasibility of integrating ISC, NAF, and IMC in a unified physical platform, we constructed a prototype system based on MM-SFGM arrays. The system was implemented on a printed circuit board (PCB), comprising three core functional arrays (ISC, NAF, IMC), a field-programmable gate array (FPGA) controller with onboard DAC/ADC modules, a bank of analog multiplexers (MUXs), and a set of transimpedance amplifiers (TIAs), as illustrated in Supplementary Fig. 71a,b.

Signal processing flow: As illustrated in Supplementary Fig. 72, incident optical signals are first received by the ISC array, where each device performs direct light-to-current transduction. These photocurrents are then converted to voltage signals by TIA_{ISC}, which are used to activate the ReLU-NAF array. The activated currents are again converted to voltage by TIA_{ReLU} and transmitted to the IMC array, where MVM is performed using programmed conductance states. The IMC output is further passed to the Sigmoid-NAF array via TIA_{IMC}, and the final output voltage is used for classification or reconstruction tasks. Each array can be programmed or reconfigured using precise pulses delivered through the FPGA and routed by the MUXs.

Training and weight Update: During the training process, the system performs supervised or unsupervised learning tasks by iteratively adjusting the weights of MM-SFGM devices configured as ISC or IMC units. As illustrated in Supplementary Fig. 71c, the optical input is applied to the ISC array, and the resulting photocurrents propagate through the ReLU NAF, IMC and Sigmoidal NAF arrays, producing predicted outputs. These outputs are digitized and compared with ground-truth labels or input data by the FPGA to compute the loss function. Based on the calculated error gradients, determines the appropriate weight updates via backpropagation. The update process is carried out by addressing specific MM-SFGM devices through a MUX network and delivering targeted write pulses.

Each MM-SFGM unit features two independently addressable split floating gates, which enable the dynamic modulation of either photoresponsivity (for ISC mode) or conductance (for IMC mode). During training, each floating gate is reprogrammed by applying ±1.2 V, 20 ns electrical pulses to the control gate. These programming pulses are routed through the MUX system, which sequentially selects devices to update based on the computed gradients.

In our current hardware setup, the FPGA board integrates a dual-channel DAC, enabling the simultaneous programming of both floating gates within a single device. Thus, updating one MM-SFGM device requires a single 20 ns operation. For a 100-device array (comprising 200 floating gates), sequential reprogramming completes within approximately 2 μs. Considering peripheral overhead—including DAC settling time, MUX switching delay, and FPGA address decoding—the total training update latency is estimated at 10–20 μs per full array update.

It is worth emphasizing that this training-related programming overhead is only incurred during weight updates or functional mode switching. Once trained, the system enters inference mode, during which no further updates are required, thanks to the non-volatile nature of the floating-gate memory. This ensures that no power is consumed to maintain programmed states, and the system can operate autonomously at nanosecond speed with femtojoule-level energy during inference.

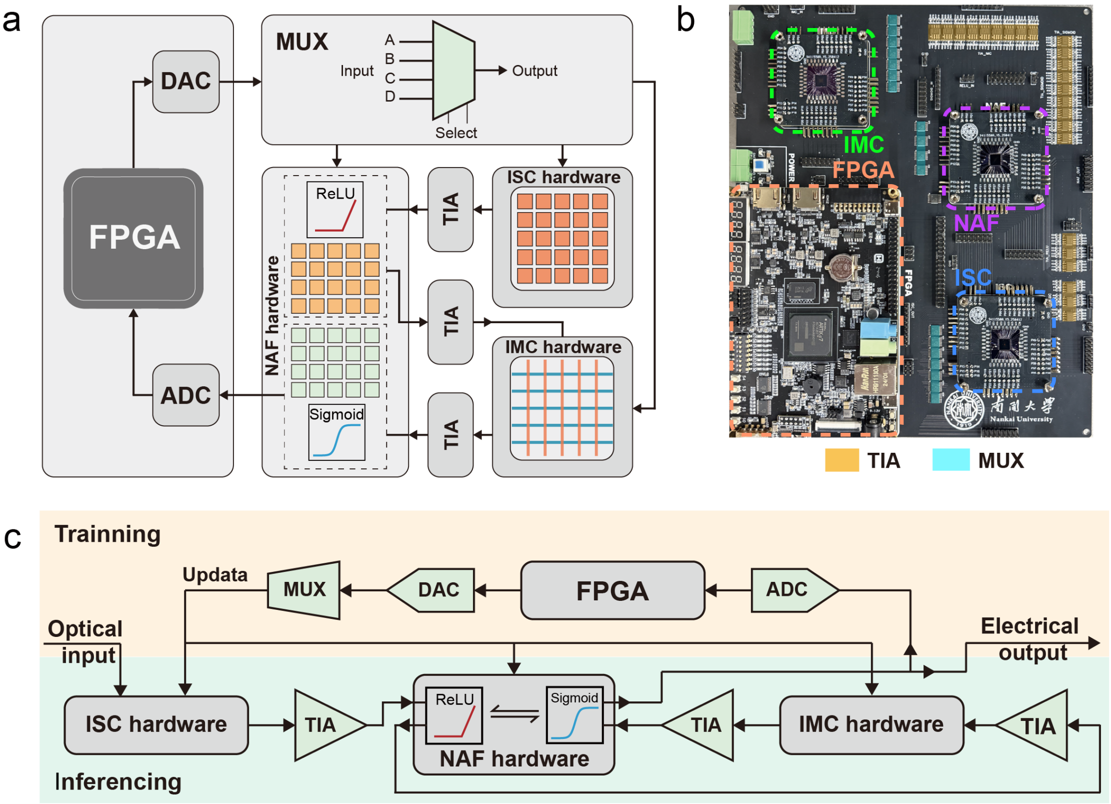

Supplementary Fig. 71| Reconfigurable neuromorphic hardware system integrating ISC, IMC, and NAF functionalities. a, Schematic diagram of the full hardware architecture, consisting of three MM-SFGM-based functional arrays for ISC, NAFs (ReLU and Sigmoid), and IMC. The arrays are coordinated by an FPGA module equipped with DAC, ADC, and a MUX network. Inter-array signal conversion is performed by TIAs. **b,** Photograph of the assembled hardware prototype. The ISC, IMC, and NAF arrays are marked by blue, green, and purple boxes, respectively, while the FPGA control unit is outlined in orange. The color-coded overlays at the bottom highlight the positions of TIAs (yellow) and MUXs (cyan) associated with the three functional arrays. **c,** Operational flow of the system. In the training phase (orange background), the FPGA computes gradients and updates the MM-SFGM states via DAC and MUX routing. In the inference phase (green background), optical signals are processed sequentially by the ISC array, ReLU and Sigmoid NAF modules, and the IMC array. TIAs are used between each stage for analog-domain signal conversion.

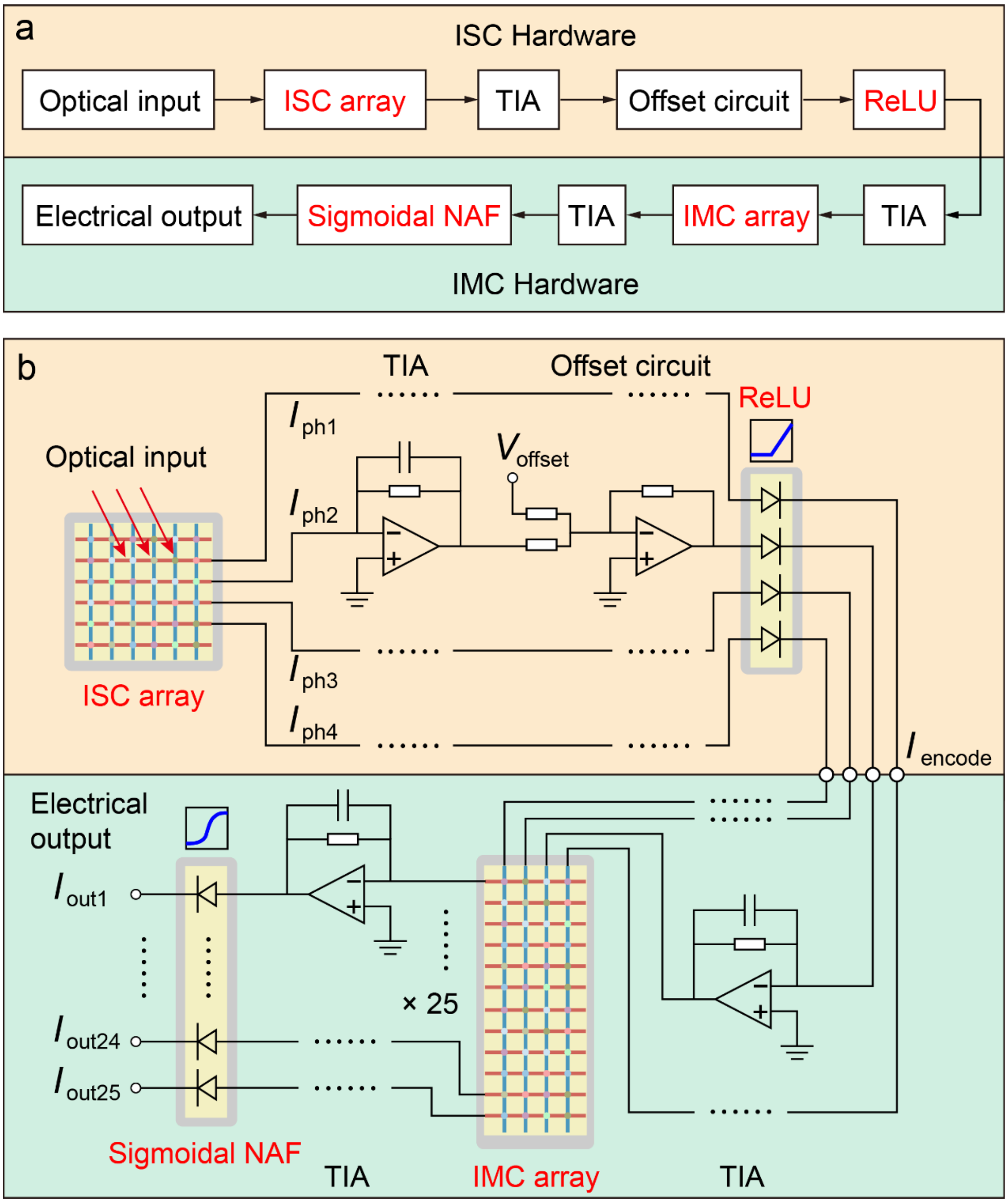

Supplementary Fig. 72| Illustration of a fully hardware NMVS based on MM-SFGM arrays. a, Process for visual information processing in the NMVS hardware. **b**, Circuit diagrams of the NMVS for visual information processing. The components marked in red are implemented using the MM-SFGM device arrays. Optical images are first sensed and processed by the ISC array. The output photocurrents are then amplified and converted into voltage signals using TIAs. An offset circuit is employed to add an offset voltage (V_{offset}) to the voltage signals. These modified signals are input into ReLU-type NAF devices. The output currents from the NAF devices are subsequently converted into voltage signals via the TIA, which are then fed into the IMC array for further processing. The output current signals from the IMC array are again converted

to voltage by the TIA and input into the Sigmoidal-type NAF devices at the output layer, yielding the final activation results.

5.2 Device-level latency and energy benchmarks

To evaluate the suitability of MM-SFGM devices for neuromorphic system deployment, we performed detailed measurements of their latency and energy consumption across different functional modes—i.e., ISC, IMC, and NAF (ReLU/Sigmoid).

Latency performance: The response time of our MM-SFGM device in all modes is primarily limited by the intrinsic carrier transport within the channel. Under optimized operation conditions, the device exhibits a response speed of approximately 8 ns, as determined by time-resolved transient current measurements (Supplementary Fig. 38). This sub-10 ns response time is among the fastest reported for analog NAF implementations and is comparable to or faster than typical CMOS-based and memristor-based systems (see Table 2 in the main text).

Energy consumption: The MM-SFGM device exhibits exceptionally low energy consumption across its various operational modes, enabling energy-efficient hardware implementation of ISC, IMC, and NAF functionalities. During reconfiguration between functional modes (e.g., switching between ISC, IMC, or different NAF behaviors), each device is programmed using ±2 V, 20 ns electrical pulses with ~100 nA current flow, corresponding to a reconfiguration energy of approximately 8 fJ per device. This operation involves updating both floating gates and is only required during functional mode switching.

For regular weight updates during training—such as adjusting the photoresponsivity in ISC or conductance in IMC—the device is programmed using ±1.2 V, 20 ns pulses, yielding a lower per-operation programming energy of ~4.8 fJ.

During inference, the MM-SFGM performs analog-domain signal processing with extremely low energy consumption. The estimated maximum dynamic energy consumption per inference operation is:

ISC: zero energy consumption (photovoltaic).

IMC: max ~64 fJ (at 1 V, ~8 μA, 8 ns).

ReLU NAF: max ~16 fJ (at 1 V, ~2 μA, 8 ns).

Sigmoid NAF: max ~ 80 aJ (at 1 V, ~10 nA, 8 ns).

These values highlight the ultra-low-power characteristics of MM-SFGM-based computing, particularly for energy-constrained edge AI scenarios. Noteworthily, the above estimates refer solely to the intrinsic energy consumption of the MM-SFGM devices. Energy contributions for the whole hardware system will be analyzed separately in the subsequent section.

5.3 System-level energy analysis

To quantify the practical energy benefits of the MM-SFGM-based neuromorphic machine vision system (NMVS), we performed a detailed analysis of the energy consumption during both the inference and training/configuration stages. The analysis considers the contributions from MM-SFGM device arrays (ISC, IMC, ReLU-NAF, and Sigmoid-NAF) as well as analog TIAs, which dominate the peripheral energy in our current setup.

Inference-stage energy consumption

During inference, the system operates as follows:

- The ISC array receives optical input and generates photocurrent.
- The four output currents are converted to voltage by 4 TIAs, then passed to the ReLU-NAF array.
- The ReLU-NAF output currents are again converted by 4 TIAs, and drive the IMC array.
- The IMC array generates 25 output currents, each processed by an individual TIA before being sent to the Sigmoid-NAF array.

Thus, a total of 33 TIAs are used during inference. Their supply power dominates the system-level inference energy. The detailed energy breakdown is as follows:

Component	Number of units	Energy per unit	Total energy
ISC computation	100 devices	0 (photovoltaic)	0
TIA (ISC→ReLU)	4	8 pJ	32 pJ
ReLU activation	4 devices	16 fJ	64 fJ
TIA (ReLU→IMC)	4	8 pJ	32 pJ
IMC computation	100 devices	64 fJ	6.4 pJ
TIA (IMC→Sigmoidal)	25	8 pJ	200 pJ
Sigmoidal activation	25 devices	80 aJ	2 fJ
**Total**			**270.4 pJ**

Thus a full inference cycle consumes approximately 270.4 pJ, achieving a quite low energy consumption despite peripheral circuit overheads.

Training-stage energy consumption

The training process involves programming the photoresponsivity of 100 ISC devices, the conductance of 100 IMC devices, and the nonlinear characteristics of 29 NAF devices (4 ReLU + 25 Sigmoid). The average programming energy is:

- ISC/IMC devices: ±1.2 V, 20 ns pulses, ~200 nA current → 4.8 fJ per device.
- NAF devices: same pulse condition → 4.8 fJ per device.

Total device-level programming energy:

- ISC: 100 × 4.8 fJ = 480 fJ
- IMC: 100 × 4.8 fJ = 480 fJ
- NAF: 29 × 4.8 fJ = 139.2 fJ
- **Total**: **1.1 pJ**

External component energy during training

Although inference is entirely self-contained, training/configuration requires additional control components, including an FPGA, analog MUXs, and DACs/ADCs. While these are excluded from Table 1 and 2 in the main text and prior literature benchmarking for consistency, we provide a conservative estimate:

- FPGA: ~50 nJ
- MUX: ~4.6 nJ (10 pJ × 460 switching)
- DAC: ~23 nJ (50 pJ × 460 operation)
- ADC: ~1.25 nJ (50 pJ × 25 operation)
- **Total**: **~79 nJ**

Note that most literature-reported energy figures for neuromorphic devices—especially those in Table 1 and 2 (main text)—do not include external control overheads. Our approach remains consistent with those practices to ensure fair comparison.

5.4 Reconfiguration overhead and reliability

A key feature of the MM-SFGM-based system is its ability to dynamically reconfigure each device into different functional modes—including ISC, IMC, and NAFs—within a unified material platform. This functional flexibility enables task-level

hardware reuse and enhances overall resource utilization. However, practical deployment requires that such reconfiguration imposes minimal time and energy overhead, and does not compromise device reliability over repeated use.

Reconfiguration latency and energy

In our hardware setup, mode reconfiguration is performed by delivering ±2 V, 20 ns pulses to the dual control gates of each MM-SFGM device. The FPGA board used in our experiments integrates a dual-channel DAC, enabling simultaneous programming of both floating gates in each device. As a result, reconfiguring a single MM-SFGM unit requires just one pulse cycle (20 ns). For a 100-device array (corresponding to 200 floating gates), the full reconfiguration can be completed sequentially within approximately 2 μs. Including the overhead from DAC settling, FPGA address decoding, and MUX switching, the total reconfiguration time per full array is estimated at 10–20 μs.

The energy associated with this reconfiguration process is also minimal. Each reprogramming event (±2 V, 20 ns, ~100 nA) consumes approximately 8 fJ per device. Thus, a full-array reconfiguration consumes only ~0.8 pJ, while excluding the energy consumed by peripheral components such as the FPGA and MUXs. Moreover, due to the non-volatile nature of the floating-gate design, once configured, the devices retain their functional roles without requiring any static power.

Reconfiguration reliability and drift tolerance

To evaluate the long-term reliability of MM-SFGM devices under repeated functional switching, we conducted comprehensive stability assessments across all four supported operation modes: ISC, IMC, and nonlinear activation with ReLU and Sigmoidal functions. The testing protocol involved cycling a single device sequentially through all four modes, with each complete cycle comprising four reconfiguration events. After every 1,000 full cycles, the corresponding output signal—photocurrent (for ISC), conductance (for IMC), and current–voltage (*I*–*V*) characteristics (for ReLU and Sigmoid)—was recorded to assess device stability across modes.

As shown in Supplementary Fig. 73, the device outputs remained highly consistent over more than 20,000 cycles of mode switching. The response levels in each mode exhibited minimal fluctuation, and no signs of drift or functional degradation were observed. This confirms that the dual-floating-gate charge storage mechanism can reliably support repeated mode reconfiguration without inducing cross-mode interference or instability.

In addition to switching stability, we also investigated long-term retention after repeated cycling. Devices were subjected to 10^6 programming cycles, after which the ON and OFF conductance states were monitored for over 10^4 seconds. As shown in Supplementary Fig. 74, both states remained stable with no evidence of current decay or charge leakage, further confirming the integrity of the non-volatile floating-gate design under extended use.

These results validate the robustness of MM-SFGM devices in dynamic computing environments. The ability to repeatedly reconfigure operational roles—while maintaining stable electrical and optoelectronic characteristics—underscores the practical applicability of this platform for reconfigurable, multifunctional neuromorphic systems.

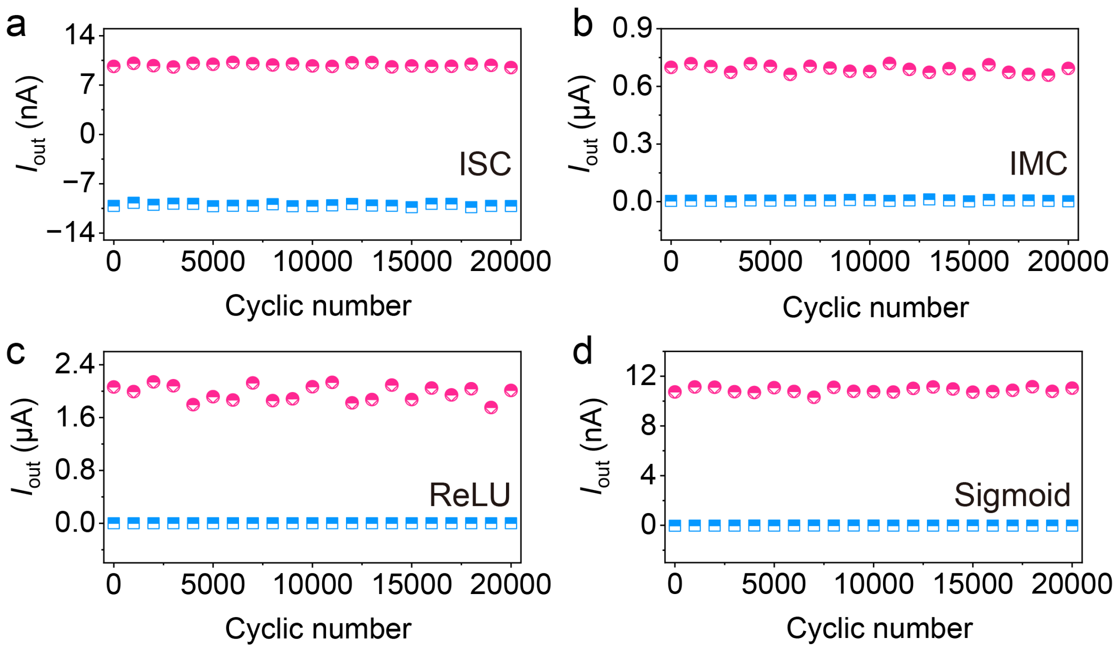

Supplementary Fig. 73| Endurance performance of MM-SFGM devices under repeated functional reconfiguration. a–d, Output signal stability of the same device under 20,000 sequential cycles in ISC (**a**), IMC (**b**), ReLU (**c**), and Sigmoidal (**d**) modes. Each full cycle involves reconfiguring the device through all four modes. After every 1,000 full cycles, representative output signals—short-circuit photocurrent (I_{SC}), drain current under 1 V bias (IMC), and nonlinear *I–V* responses (ReLU and Sigmoidal)—are recorded to evaluate functional retention. Test conditions: I_{SC} mode with 125 nW illumination and photoresponsivity of ±80 mA/W; IMC mode with 1 V input and conductance states of 10 pS and 7 µS; ReLU and Sigmoidal modes with ±1 V input. I_{out} represents the measured output currents for each mode.

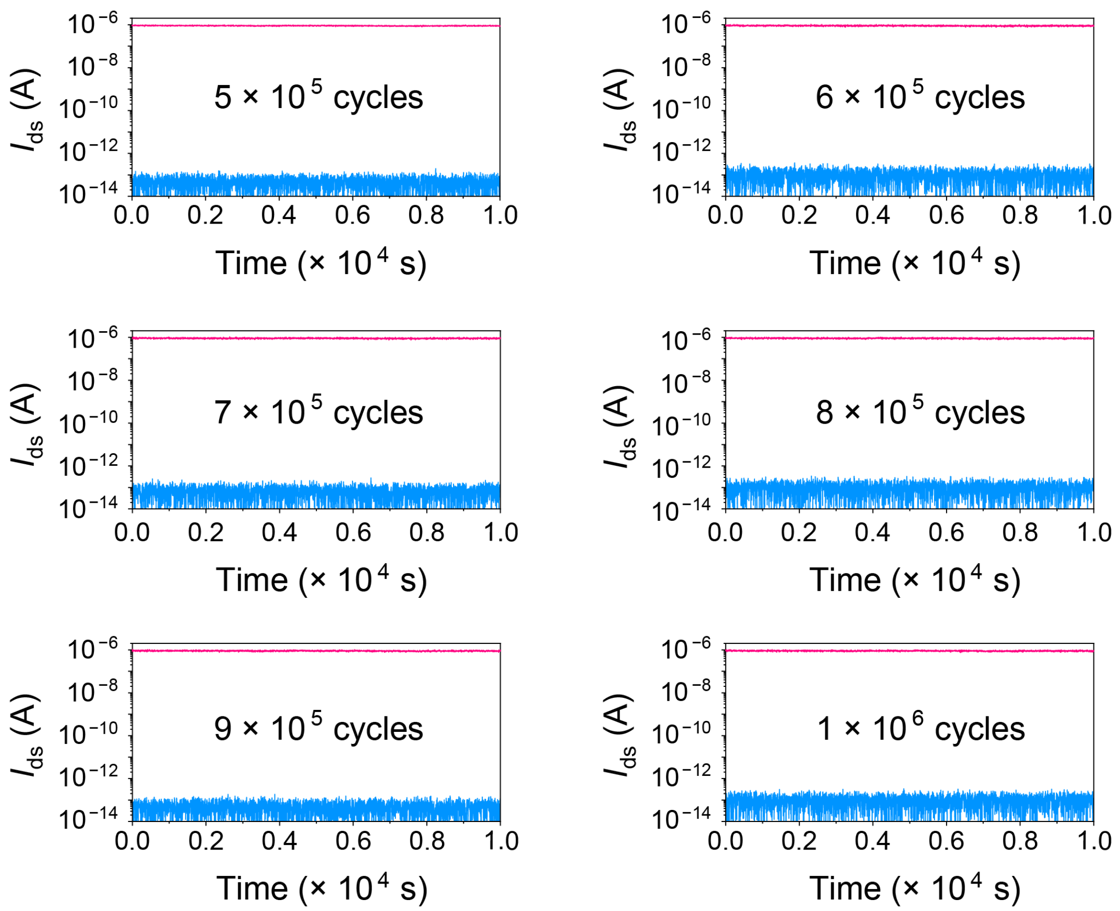

Supplementary Fig. 74| *I-t* characteristics of the MM-SFGM after repeated switching cycles. Drain current (I_{ds}) measured in the on (p–p, pink) and off (n–p, blue) states after 5×10^5 to 1×10^6 switching cycles. Measurements were conducted under a constant read voltage over 10^4 seconds. The stable current levels and low leakage confirm that the charge stored in the floating gate with no observable degradation or drift even after extensive cycling.

5.5 Quantized weight precision and impact on training accuracy

In neuromorphic hardware systems, weight precision is often limited due to constraints in analog programmability, noise tolerance, and peripheral complexity. While digital neural networks typically employ 32-bit floating-point precision, many hardware-friendly training schemes adopt quantized weights ranging from 3 to 8 bits. This precision reduction naturally raises questions regarding inference accuracy and training efficiency.

In our MM-SFGM device, both photoresponsivity (for ISC mode) and conductance (for IMC mode) can be programmed with an effective resolution of 6 bits, corresponding to 64 discrete, non-volatile states. To evaluate the impact of this quantization level on system performance, we conducted a series of simulations using experimentally extracted device parameters. As discussed in Supplementary Note 4.4, two distinct CNN architectures were tested on the MNIST and CIFAR-10 datasets, respectively. For the MNIST task, the 6-bit quantized network achieved ~97.0% accuracy, which is only 1% lower than the ideal 32-bit baseline. For the more challenging CIFAR-10 dataset, a deeper network architecture using 6-bit weights reached ~88.0% accuracy, closely approaching the software baseline of 90.0%. These results are consistent with prior studies in resistive memory networks[19], which reported quite high inference accuracy using only 4-bit precision in memristor arrays.

Importantly, the use of quantized and discretized weights simplifies hardware implementation of training procedures. During backpropagation, the gradient calculations performed on the FPGA can directly map into discrete pulse operations, reducing computational burden and eliminating the need for complex weight interpolation or floating-point arithmetic. This enables fast, energy-efficient, and hardware-friendly learning, particularly advantageous in edge computing scenarios.

Therefore, these findings confirm that 6-bit precision in MM-SFGM devices is sufficient to support accurate training and inference in representative deep learning tasks, and that weight approximation due to limited resolution introduces only negligible accuracy degradation.

Supplementary Note 6. Comparison with state-of-the-art neuromorphic platforms

6.1 Comparison with existing neuromorphic materials and devices

To contextualize the significance of our MM-SFGM device, we compare its performance with representative neuromorphic devices based on 2D heterostructures, ferroelectric materials, phase-change memory (PCM), and oxide-based ReRAMs.

Table 1 in the main text summarizes the comparison. In terms of switching energy, MM-SFGM exhibits exceptionally low energy requirements: ~4.8 fJ for weight/programming operations. outperforming ferroelectric and PCM devices (which typically operate in the pJ–nJ range). For functional versatility, most existing systems implement only one or two functions (e.g., ISC + IMC), while MM-SFGM enables full ISC + IMC + dual-mode NAF within the same reconfigurable device structure.

In terms of endurance, although the MM-SFGM has been validated up to 10^6 cycles in each functional mode, we acknowledge that certain ferroelectric and PCM devices have reported endurance exceeding 10^{11} cycles. Nonetheless, we observe no noticeable degradation in MM-SFGM behavior during repeated mode reconfigurations and long-term retention testing (see Supplementary Note 5.4), suggesting strong promise for future endurance optimization.

For scalability, MM-SFGM leverages wafer-scale CVD-grown 2D materials and ALD-deposited HfO_2 gate dielectrics, allowing high device yield and monolithic compatibility. This avoids the thermal stress and crystallinity constraints often seen in PCM or ferroelectric integration, and offers favorable prospects for large-area, high-density neuromorphic arrays.

Crucially, unlike prior works where nonlinear operations are offloaded to peripheral circuits or software processors, our MM-SFGM integrates dual-mode hardware-based ReLU and Sigmoid activation directly into the array, closing a key gap in the neuromorphic processing stack. Combined with sub-10 ns latency and femtojoule-level energy consumption across all modalities, this trifunctional, reconfigurable platform sets a new benchmark for compact, low-power, and versatile neural hardware.

6.2 Comparison with existing NAF hardware platforms

A wide range of hardware platforms have been developed to implement NAFs such as ReLU and Sigmoid, including CMOS circuits, Mott-transition devices, digital processors (e.g., FPGA/ASIC), ADCs, and emerging memory-based analog systems (e.g., MRAM, PCM). Table 2 in the main text provides a quantitative comparison of these approaches with our MM-SFGM platform, covering key performance metrics such as energy consumption, response time, area efficiency, and configurability.

CMOS-based implementations often rely on analog interpolation circuits or lookup tables to emulate nonlinear functions, offering sub-nanosecond latency but at the cost of higher energy consumption (typically in the μW to mW power costs) and larger circuit footprint due to the need for operational amplifiers, reference voltages, or flash memory arrays. More importantly, although certain CMOS architectures allow threshold tuning, they generally cannot physically reconfigure between distinct nonlinear functions such as ReLU and Sigmoid within the same circuit unit. Each function typically requires a separate hardwired design (Supplementary Fig. 75), limiting per-cell flexibility and inflating silicon area.

Digital implementations (e.g., using FPGA or ASICs) offer programmability but incur latency overhead due to serial computation and data movement. Recent studies report delays in the range of 1–60 ns per activation and typical power costs exceeding 1–100 mW when executing NAFs in stochastic computing or low-precision digital logic.

Mott-transition neurons, such as those based on VO_2, provide intrinsic nonlinearity with sharp switching transitions, effectively mimicking ReLU-like behavior. However, they are constrained by relatively high latency (~60 ns) and substantial energy consumption (~200 pJ) due to their thermal switching mechanism. Moreover, only ReLU activation has been demonstrated on such platforms to date, further limiting functional versatility.

Our MM-SFGM device, in contrast, integrates both ReLU and Sigmoid-type NAFs in a single physical unit through programmable split-gate structures. The activation mode is selected via a one-time electrical pulse (±2 V, 20 ns), offering non-volatile and

per-device reconfigurability without standby power consumption. The device exhibits ultralow activation energy (<16 fJ for ReLU, ~80 aJ for Sigmoid) and nanosecond-scale latency (<8 ns), combining high speed, energy efficiency, and compactness in a single monolithic structure.

These capabilities make MM-SFGM-based NAF arrays highly attractive for large-scale neuromorphic systems, where layer-wise heterogeneity, functional reassignment, and system-level adaptability are increasingly essential.

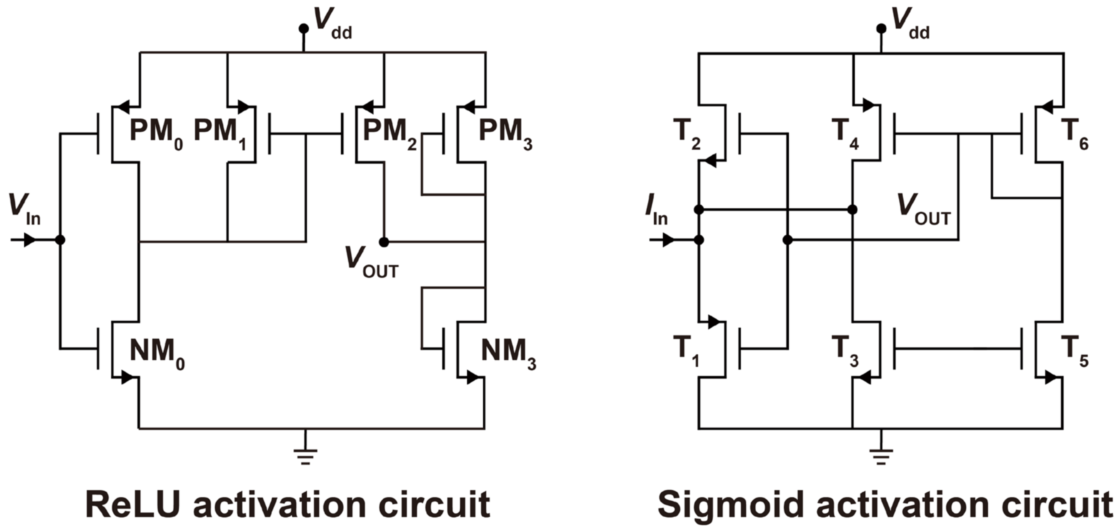

Supplementary Fig. 75| The diagrams of CMOS-based ReLU (left) and Sigmoid (right) activation circuits.

6.3 Advantages of monolithic and single-device integration

Traditional neuromorphic architectures typically rely on a heterogeneous combination of sensors, memory units, and nonlinear activation circuits. This fragmented approach results in increased interconnect overhead, higher latency, and greater power consumption due to frequent signal conversions and data transfers between discrete components. Additionally, most activation functions are implemented in separate digital or analog circuits, requiring significant area and circuit complexity to emulate nonlinear transformations such as ReLU or Sigmoid.

In contrast, the MM-SFGM introduces a monolithic integration paradigm in which sensing (ISC), weighted computing (IMC), and nonlinear activation (NAF) are all realized within a single reconfigurable device structure based on a unified material system. This approach offers several key advantages:

Physical co-location of functions: All signal transformations, from sensing to final activation, are executed within the same material and structural stack, drastically reducing the need for inter-module signal routing and peripheral interfacing.

Reduced latency and energy overhead: By eliminating the need to transmit intermediate results between discrete processing blocks, the MM-SFGM system avoids repeated I/O conversions and clock synchronization. This results in a significantly shortened signal path and nanosecond delay across the entire processing pipeline.

Non-volatile configurability: Each MM-SFGM unit can be electrically reconfigured to serve as a sensor, synapse, or nonlinear activation node by modulating the charge states in its split floating gates. Crucially, the reconfigured state is non-volatile, enabling the system to retain its functional configuration without standby power consumption. This architecture allows flexible scheduling and hardware reuse across different computational tasks.

Importantly, the mode reassignment can be performed dynamically via FPGA-controlled addressing and multiplexing, enabling flexible reconfiguration during task switching or between inference cycles. For instance, a subarray initially used for sensing can be reassigned as a hidden-layer computing unit, or ReLU-mode devices can

be reprogrammed as Sigmoid activators, based on evolving algorithmic demands. Although the current demonstration is based on sequential programming with microsecond-level latency, the underlying hardware supports dynamic functional reassignment, laying the groundwork for adaptive sensor-processor pipelines in edge-AI and real-time robotics.

Scalable and layout-efficient design: Compared with circuit-based NAF implementations (e.g., CMOS activation modules or FPGA-embedded nonlinear logic), our single-device approach offers much higher integration density and layout flexibility. It enables localized function assignment within the array, paving the way for dynamically reconfigurable computing fabrics.

The convergence of these advantages makes the MM-SFGM device architecture particularly well-suited for deployment in energy- and space-constrained edge-AI systems, where conventional silicon-based platforms are often limited by size, power, and complexity. Application domains such as robotic vision, event-driven sensing, and autonomous navigation can benefit directly from this trifunctional integration, where real-time processing, low-latency decision-making, and in-situ learning are essential. Moreover, the ability to reconfigure device functions (e.g., switching from a sensing node to a nonlinear activation unit) within microseconds offers a practical route toward adaptive sensor-processor pipelines, capable of reallocating resources or reprogramming functionalities on demand in response to task dynamics or environmental feedback.

Altogether, these features demonstrate the feasibility and benefit of collapsing multiple computing primitives into a single physical unit, enabling a new class of low-power, high-speed, and highly integrable neuromorphic computing systems engineered for next-generation intelligent machines.

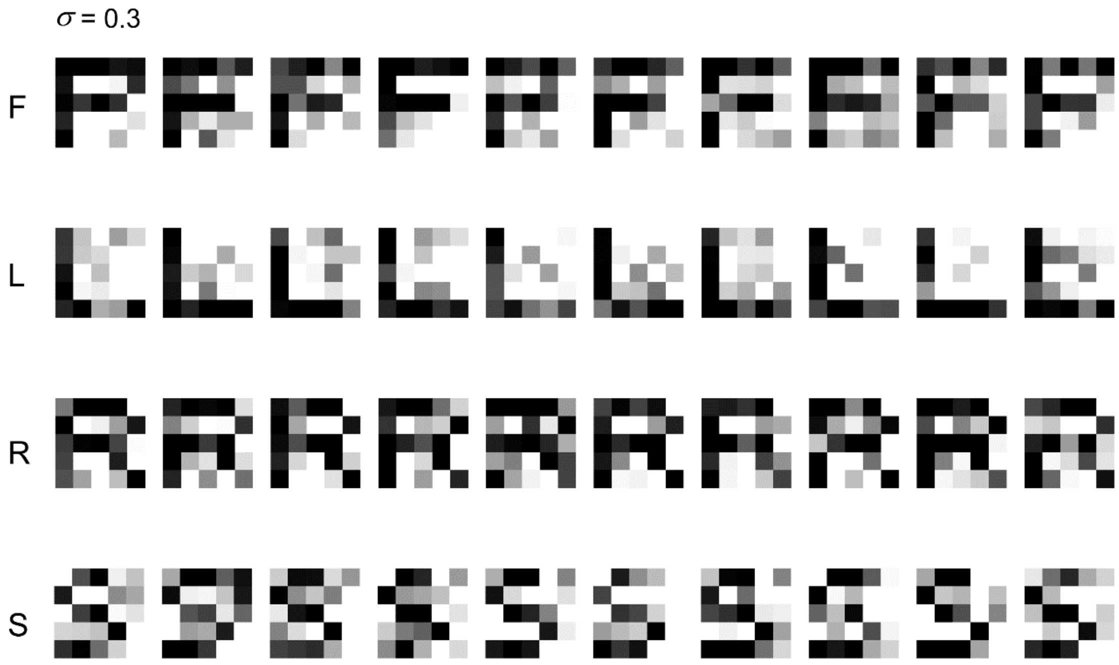

Supplementary Fig. 76| Datasets of letters "F", "L", "R", and "S". 5 × 5-pixel images of letters "F", "L", "R", and "S" with randomly added noises (σ = 0.3) are generated as the training and test datasets for classification task. The training and test datasets involve 1000 and 200 randomly generated images, respectively. Notably, this dataset is not derived from the standard MNIST dataset, nor is it a downsampled subset.

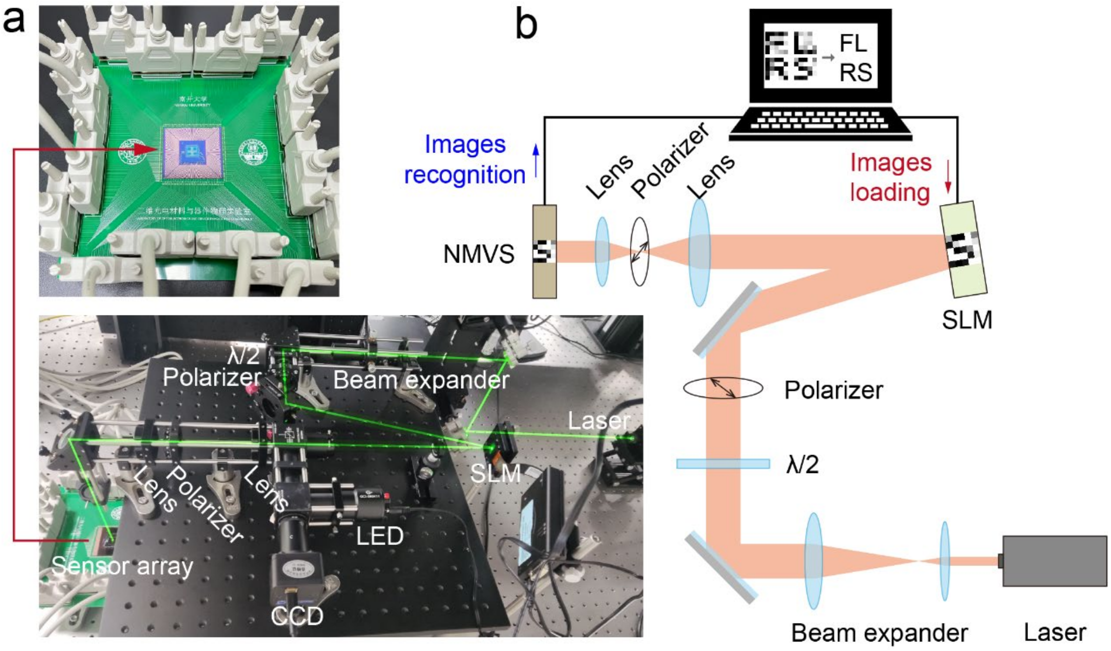

Supplementary Fig. 77| Experimental set-up for ISC operation. a,**b**, Photographs (**a**) and schematic illustration (**b**) of the experimental set-up. A 532 nm laser is used as the input light, which is expanded and linearly polarized, and 5 × 5-pixel images are loaded to the laser beam via a spatial light modulator (SLM) operated in intensity-modulation mode. Then the input images are adjusted to 1 mm × 1 mm (the size of the sensor array) via optical lenses, and projected to the 5 × 5 × 4 sensor array. The 5 × 5 × 4 device array is directly connected with a 400-pins probe card, which can communicate with PCB testing system. The output currents of the sensor array are further activated by the ReLU or Sigmoidal NAFs executed in the neuron-mode device array, outputting encoded data to the following IMC hardware.

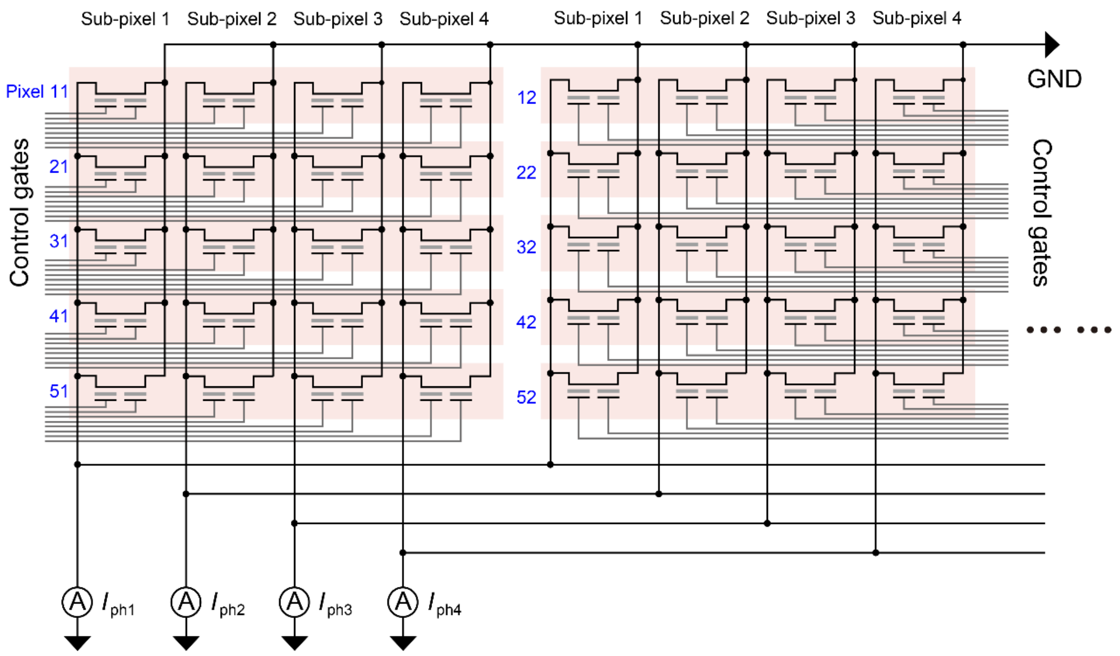

Supplementary Fig. 78| Circuit of the 5 × 5 × 4 sensor array to perform ISC. Each pink box represents one pixel (5 × 5 pixels), and each pixel consists of 4 subpixels. The MM-SFGMs with the same subpixel index are connected in parallel. While 5 × 5-pixel images are illuminated on the sensor array, the photocurrents generated by all the 100 devices via photovoltaic effect are accumulated following Kirchhoff's current law, outputting four photocurrent signals (I_{ph1}, I_{ph2}, I_{ph3} and I_{ph4}). The weight distribution of the sensory network is programmed by applying paired V_{CG} pulses to the control gates.

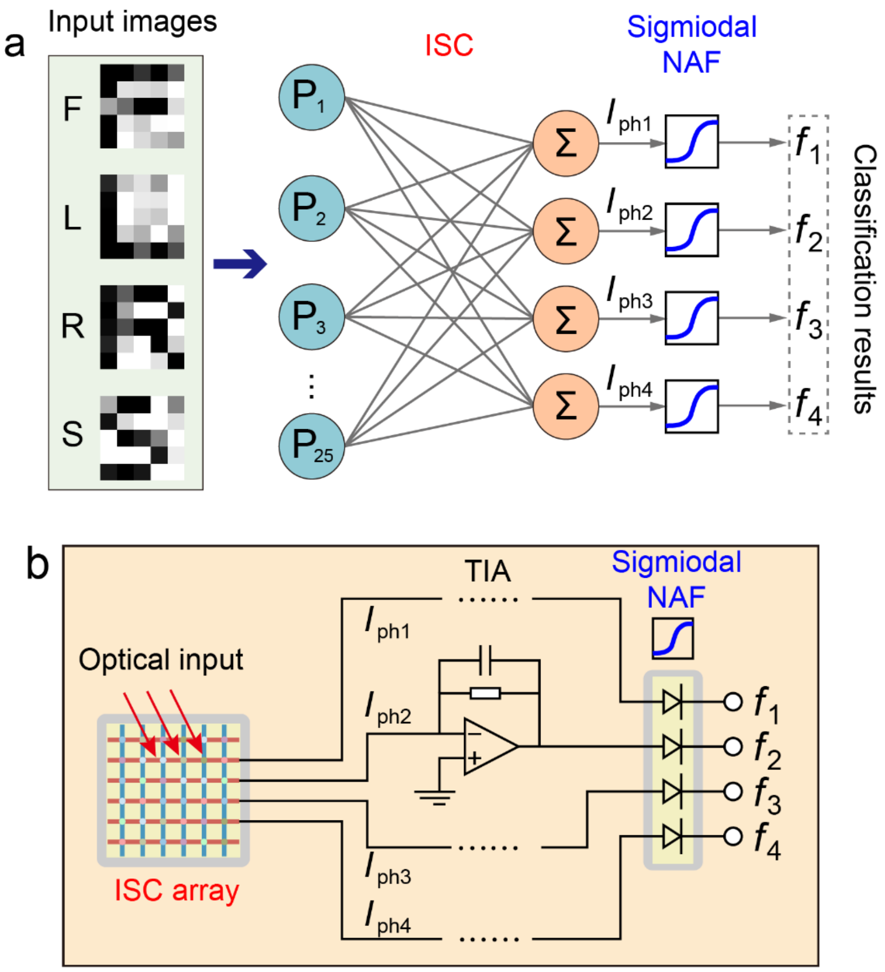

Supplementary Fig. 79| Structure of a NMVS for classification task. a, Schematic of a NMVS consisting of a 25 × 4 ISC device array and four Sigmoidal-type NAF devices. **b**, Circuit diagram of the NMVS for classification task. 5 × 5-pixel images are projected into the 25 × 4 ISC array, with the output four photocurrent signals (I_{ph1}, I_{ph2}, I_{ph3}, and I_{ph4}) being activated by four Sigmoidal-type NAF devices, thereby outputting probabilities for letters “F”, “L”, “R”, and “S” (f_1, f_2, f_3, and f_4).

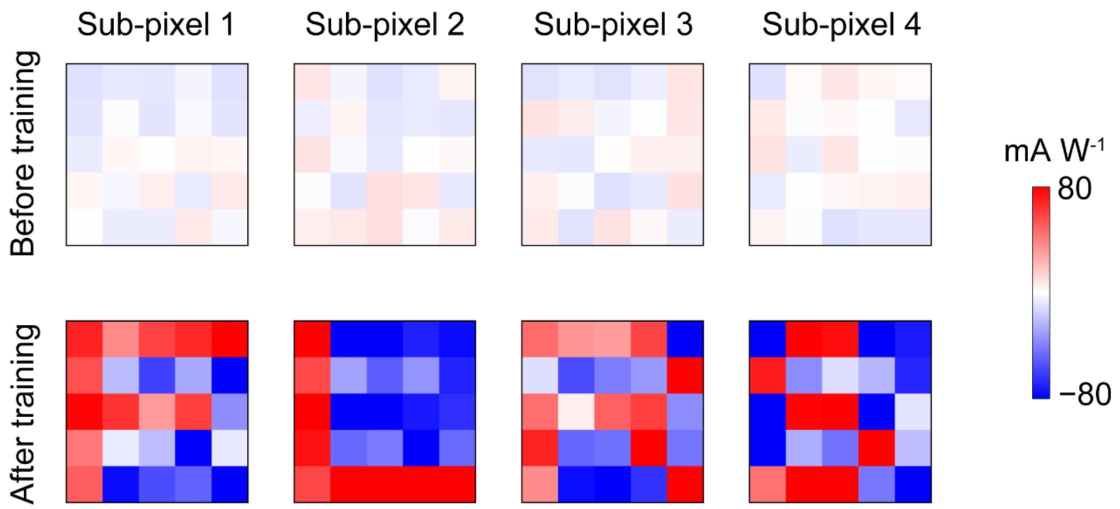

Supplementary Fig. 80| Photoresponsivity configurations of the sensory network before and after 30 epoch training. Sub-pixel 1, 2, 3, and 4 represent the sensors with the same subpixel index n (n = 1, 2, 3, 4) in each pixel (5 × 5), which are connected in parallel.

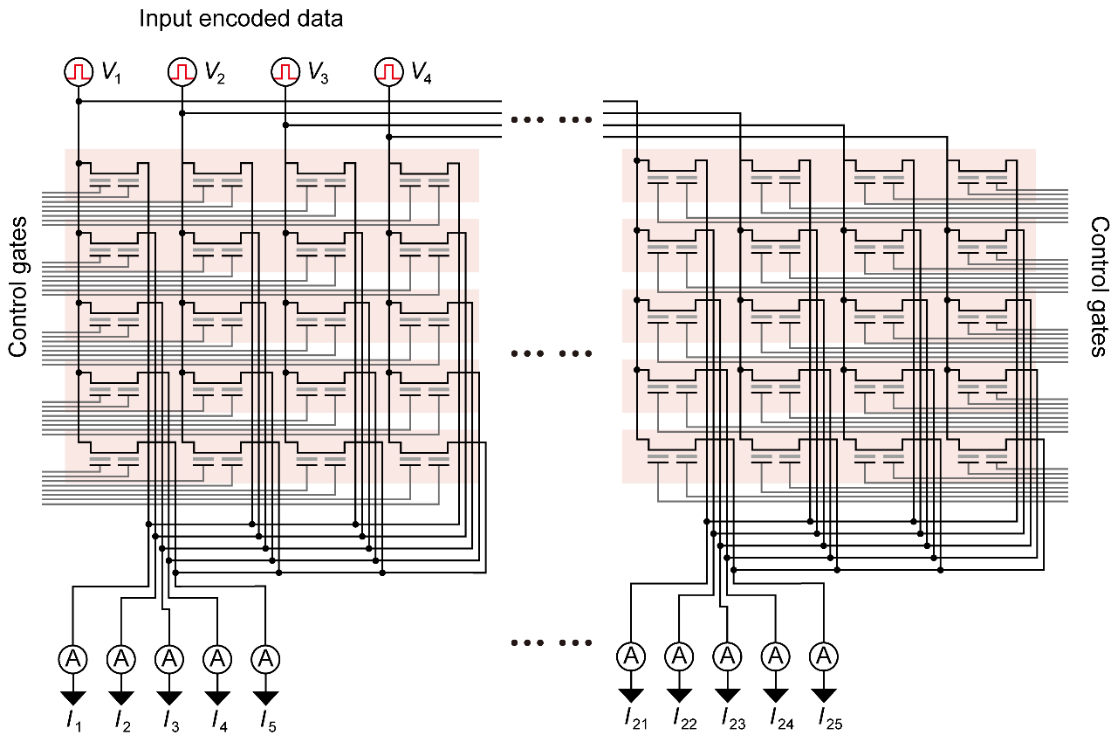

Supplementary Fig. 81| Circuit of the 4 × 25 MM-SFGM array in synapse mode to perform IMC. Four voltage pulses are applied as the input signals, and each voltage is applied on 25 devices with the same subpixel index (m = 1, 2, 3, 4). The generated currents by the 4 devices in each pixel (n = 1, 2, …, 25) are accumulated following Kirchhoff's current law and output a current signal I_n. The weight (conductance) of each device is modulated by applying paired V_G pulses to the control gates (CG1 and CG2).

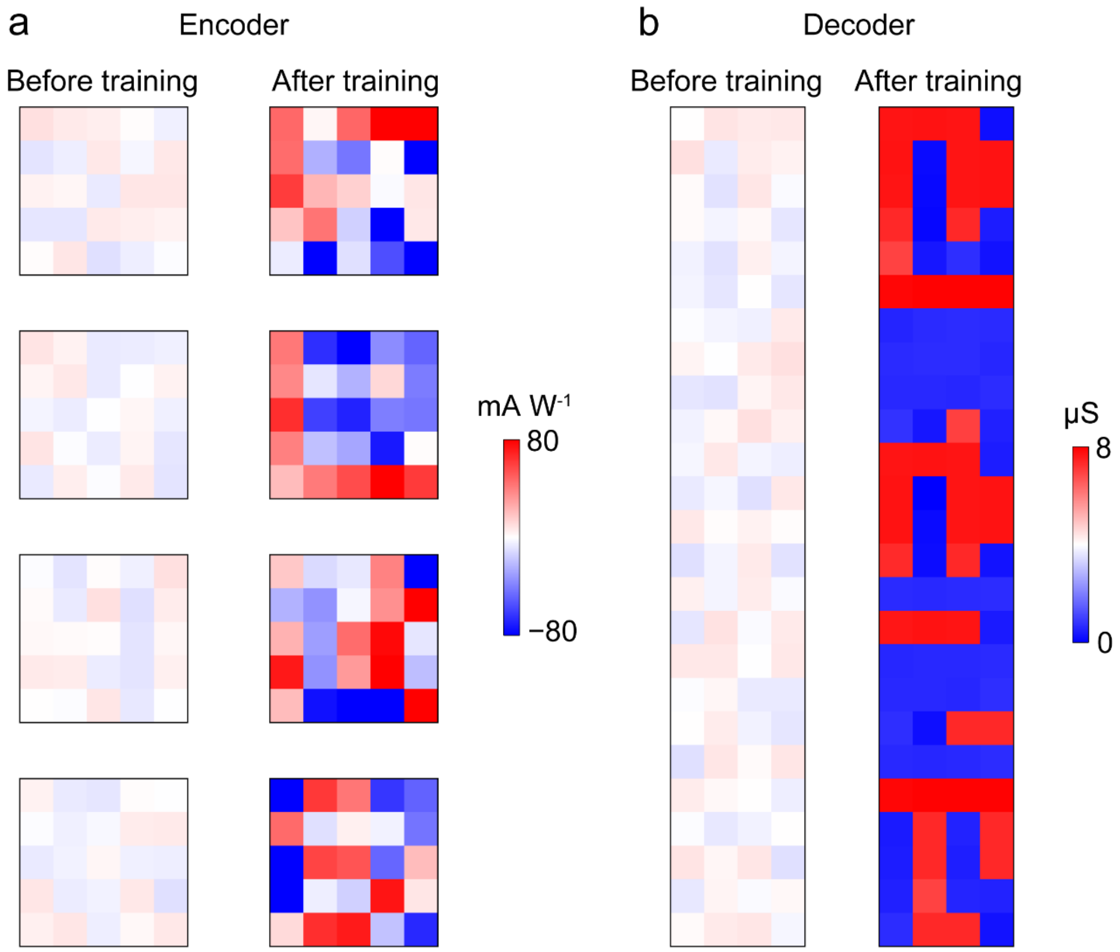

Supplementary Fig. 82| Weight configurations of the autoencoder before and after training. a, Weight configurations of the sensor-mode MM-SFGM array before and after training, as an encoder to perform ISC. **b**, Weight configurations of the synapse-mode MM-SFGM array before and after training, as a decoder to perform IMC.

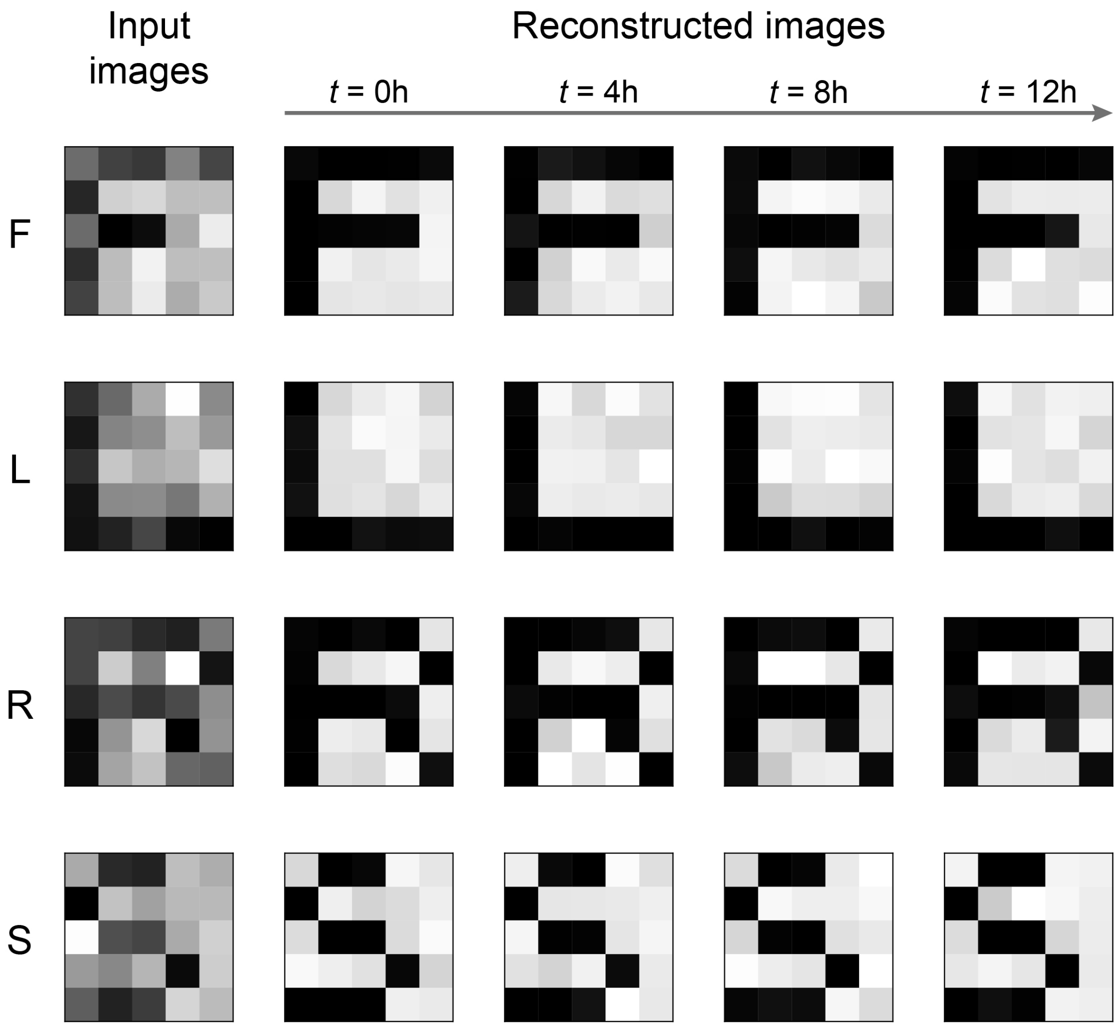

Image	PSNR			SSIM		
	t = 4 h	*t* = 8 h	*t* = 12 h	*t* = 4 h	*t* = 8 h	*t* = 12 h
**F**	47.41181	23.18784	42.9504	0.95117	0.95278	0.95405
**L**	47.17052	34.1904	38.14559	0.94827	0.95102	0.94927
**R**	25.05176	29.09386	28.57048	0.95361	0.95178	0.9505
**S**	38.11757	27.97619	33.55561	0.95292	0.95327	0.95087

Supplementary Fig. 83| Time-dependent stability of image reconstruction using pre-trained NMVS with locally stored weights. Input grayscale patterns (“F”, “L”, “R”, “S”) were reconstructed using the autoencoder implemented with MM-SFGM arrays. Reconstruction was evaluated at *t* = 0, 4, 8, and 12 hours after initial training, demonstrating stable performance over extended periods. The table summarizes the

corresponding peak signal-to-noise ratio (PSNR) and structural similarity index (SSIM) for each reconstruction time point. All reconstructed outputs maintained high visual fidelity (SSIM > 0.94) and minimal distortion, confirming robust retention of learned weights in the non-volatile MM-SFGM hardware.

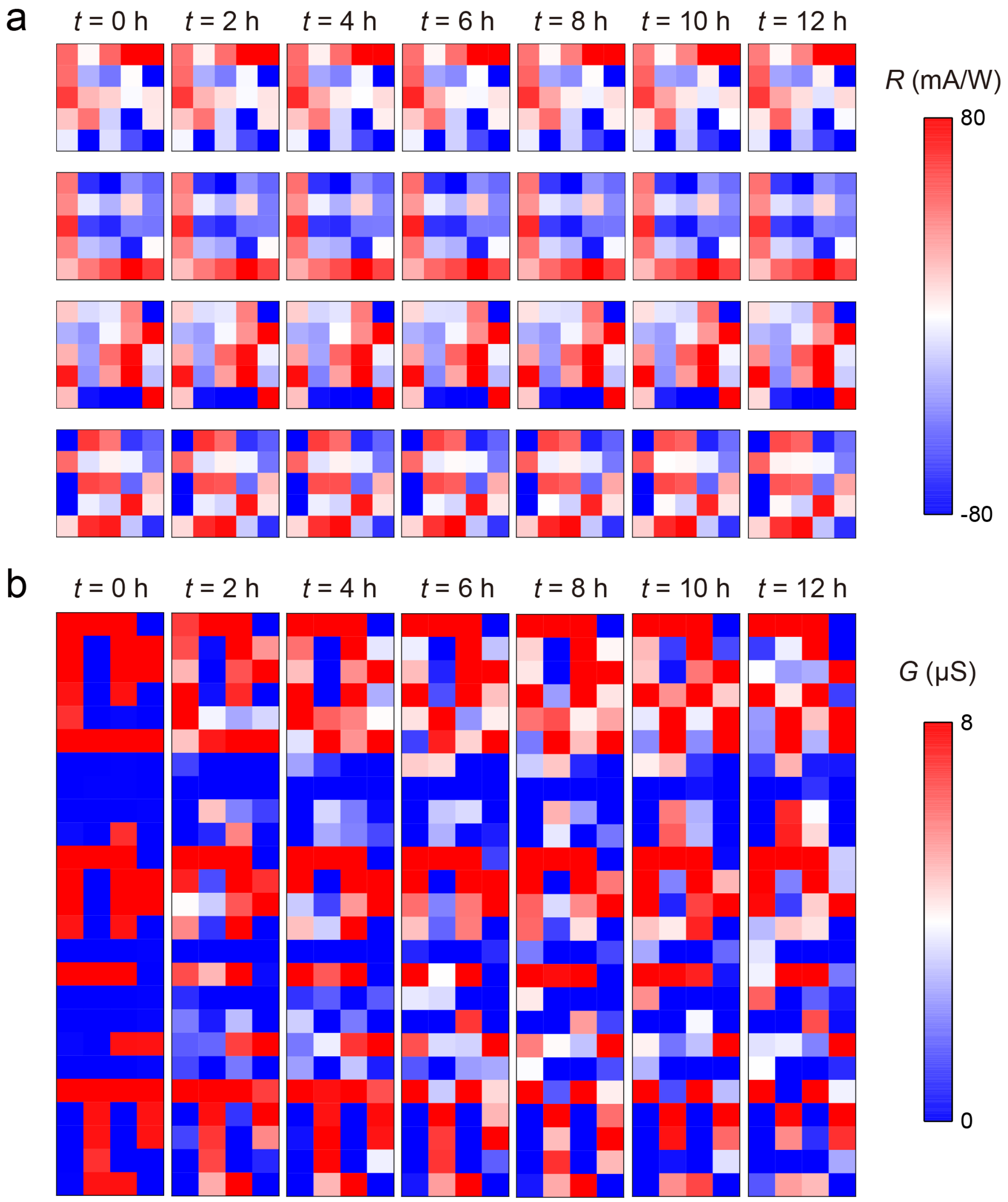

Supplementary Fig. 84| Retention of trained weights in MM-SFGM arrays over time. a, Evolution of stored photoresponsivity (R, in mA W^{-1}) across the array for encoding, measured at multiple time points from t = 0 to 12 hours. **b,** Evolution of stored conductance (G, in μS) across the array for decoding, measured over the same time period. The stable spatial patterns observed in both R and G matrices confirm excellent retention of non-volatile analog states, ensuring the long-term stability of weights stored in the photoresponsivity and conductance domains.

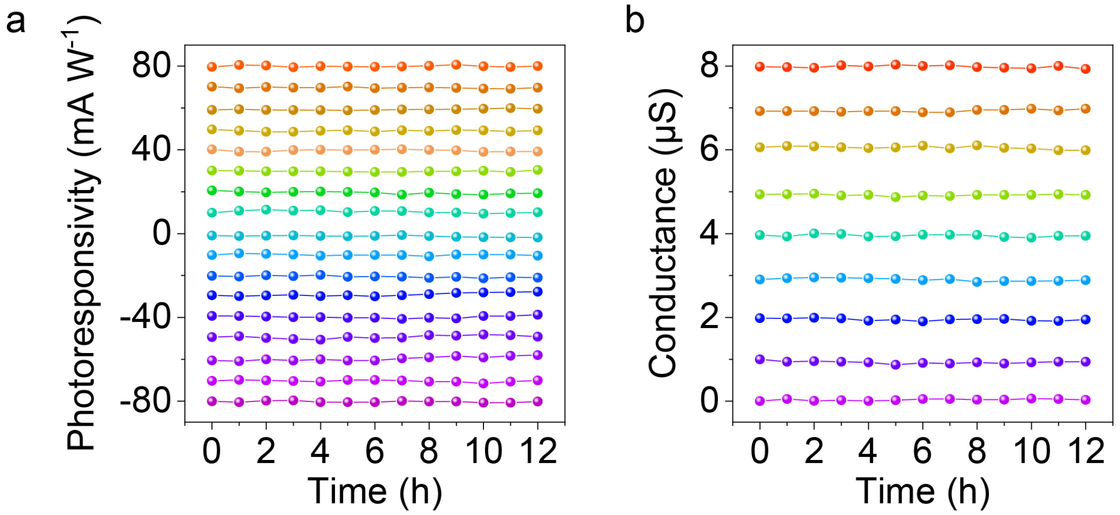

Supplementary Fig. 85| Retention performance of photoresponsivity and conductance states over 12 hours. a, Time-dependent stability of multiple photoresponsivity states ranging from –80 to +80 mA/W, representative of programmable weights in the ISC array. **b**, Retention of conductance states between 0 and 8 μS for IMC operation. Each trace corresponds to one initially programmed level, monitored over a 12-hour period at room temperature. Both photoresponsivity and conductance values remain highly stable, with negligible drift, confirming the excellent non-volatility of the floating-gate storage mechanism.

Supplementary references

1. Li, J., et al. Synthesis of wafer-scale ultrathin graphdiyne for flexible optoelectronic memory with over 256 storage levels. *Chem* **7**, 1284-1296 (2021).
2. Kresse, G., Furthmüller, J. Efficient iterative schemes for ab initio total-energy calculations using a plane-wave basis set. *Phys. Rev. B* **54**, 11169 (1996).
3. Hammer, B., Hansen, L.B., Nørskov, J.K. Improved adsorption energetics within density-functional theory using revised Perdew-Burke-Ernzerhof functionals. *Phys. Rev. B* **59**, 7413 (1999).
4. Perdew, J.P., Burke, K., Ernzerhof, M. Generalized gradient approximation made simple. *Phys. Rev. Lett.* **77**, 3865 (1996).
5. Grimme, S. Semiempirical GGA-type density functional constructed with a long-range dispersion correction. *J. Comput. Chem.* **27**, 1787-1799 (2006).
6. Henkelman, G., Uberuaga, B.P., Jónsson, H. A climbing image nudged elastic band method for finding saddle points and minimum energy paths. *J. Chem. Phys.* **113**, 9901-9904 (2000).
7. Zou, J., et al. An artificial L-ReLU neuron with asymmetric diffusive memristor for high-accuracy neuromorphic systems. *Adv. Funct. Mater.* **35**, 2423267 (2025).
8. Kim, D., Lee, B.-g. Energy-and area-efficient CMOS neuron and max pooling circuit for RRAM-based CNN accelerators. *IEEE Access* **13**, 84329-84340 (2025).
9. Hsieh, Y.-T., Anjum, K., Huang, S., Kulkarni, I., Pompili, D. Neural network design via voltage-based resistive processing unit and diode activation function-a new architecture. *2021 IEEE International Midwest Symposium on Circuits and Systems (MWSCAS)*, 59-62 (2021).
10. Van Pham, K., Min, K.-S. Non-ideal effects of memristor-CMOS hybrid circuits for realizing multiple-layer neural networks. *2019 IEEE International Symposium on Circuits and Systems (ISCAS)*, 1-5 (2019).
11. Zhu, J., Huang, Y., Yang, Z., Tang, X., Ye, T.T. Analog implementation of reconfigurable convolutional neural network kernels. *2019 IEEE Asia Pacific Conference on Circuits and Systems (APCCAS)*, 265-268 (2019).
12. Hanamashetti, S., Vadde, V., Muralidharan, B. A comparative study on ReLU Implementation using TMDFETs. *Phys. Scr.* **99**, 075923 (2024).
13. Surekcigil Pesch, I., Bestelink, E., de Sagazan, O., Mehonic, A., Sporea, R.A. Multimodal transistors as ReLU activation functions in physical neural network classifiers. *Sci. Rep.* **12**, 670 (2022).
14. Ahn, J.-H., et al. On-chip adaptive matching learning with charge-trap synapse device and ReLU activation circuit. *Solid-State Electron.* **186**, 108177 (2021).
15. Lee, K.-H., et al. Highly linear analog spike processing block integrated with an AND-type flash array and CMOS neuron circuits. *IEEE Trans. Electron Devices* **69**, 6065-6071 (2022).
16. Li, G.H., et al. All-optical ultrafast ReLU function for energy-efficient nanophotonic deep learning. *Nanophotonics* **12**, 847-855 (2023).
17. Liu, L., et al. A mass transfer technology for high-density two-dimensional device integration. *Nat. Electron.* **8**, 135-146 (2025).
18. Lu, D., et al. Monolithic three-dimensional tier-by-tier integration via van der

Waals lamination. *Nature* **630**, 340-345 (2024).
19. Yao, P., et al. Fully hardware-implemented memristor convolutional neural network. *Nature* **577**, 641-646 (2020).
